# Neutralizing antibody responses over time in demographically and clinically diverse individuals recovered from SARS-CoV-2 infection in the United States and Peru: A cohort study

**DOI:** 10.1371/journal.pmed.1003868

**Published:** 2021-12-06

**Authors:** Shelly Karuna, Shuying Sue Li, Shannon Grant, Stephen R. Walsh, Ian Frank, Martin Casapia, Meg Trahey, Ollivier Hyrien, Leigh Fisher, Maurine D. Miner, April K. Randhawa, Laura Polakowski, James G. Kublin, Lawrence Corey, David Montefiori, for the HVTN 405/HPTN 1901 Study Team

**Affiliations:** 1 Vaccine and Infectious Disease Division, Fred Hutchinson Cancer Research Center, Seattle, Washington, United States of America; 2 Brigham & Women’s Hospital, Harvard Medical School, Boston, Massachusetts, United States of America; 3 Department of Medicine, Division of Infectious Diseases, University of Pennsylvania, Philadelphia, Pennsylvania, United States of America; 4 Asociación Civil Selva Amazónica, Iquitos, Peru; 5 Division of AIDS, NIAID, NIH, Bethesda, Maryland, United States of America; 6 Department of Surgery and Duke Human Vaccine Institute, Duke University Medical Center, Durham, North Carolina, United States of America; Burnet Institute, AUSTRALIA

## Abstract

**Background:**

People infected with Severe Acute Respiratory Syndrome Coronavirus 2 (SARS-CoV-2) experience a wide range of clinical manifestations, from asymptomatic and mild illness to severe illness and death, influenced by age and a variety of comorbidities. Neutralizing antibodies (nAbs) are thought to be a primary immune defense against the virus. Large, diverse, well-characterized cohorts of convalescent individuals provide standardized values to benchmark nAb responses to past SARS-CoV-2 infection and define potentially protective levels of immunity.

**Methods and findings:**

This analysis comprises an observational cohort of 329 HIV–seronegative adults in the United States (*n* = 167) and Peru (*n* = 162) convalescing from SARS-CoV-2 infection from May through October 2020. The mean age was 48 years (range 18 to 86), 54% of the cohort overall was Hispanic, and 34% identified as White. nAb titers were measured in serum by SARS-CoV-2.D614G Spike-pseudotyped virus infection of 293T/ACE2 cells. Multiple linear regression was applied to define associations between nAb titers and demographic variables, disease severity and time from infection or disease onset, and comorbidities within and across US and Peruvian cohorts over time. nAb titers peaked 28 to 42 days post-diagnosis and were higher in participants with a history of severe Coronavirus Disease 2019 (COVID-19) (*p* < 0.001). Diabetes, age >55 years, male sex assigned at birth, and, in some cases, body mass index were also independently associated with higher nAb titers, whereas hypertension was independently associated with lower nAb titers. nAb titers did not differ by race, underlying pulmonary disease or smoking. Two months post-enrollment, nAb ID50 (ID80) titers declined 3.5 (2.8)-fold overall. Study limitations in this observational, convalescent cohort include survivorship bias and missing early viral loads and acute immune responses to correlate with the convalescent responses we observed.

**Conclusions:**

In summary, in our cohort, nAb titers after SARS-CoV-2 infection peaked approximately 1 month post-diagnosis and varied by age, sex assigned at birth, disease severity, and underlying comorbidities. Our data show great heterogeneity in nAb responses among people with recent COVID-19, highlighting the challenges of interpreting natural history studies and gauging responses to vaccines and therapeutics among people with recent infection. Our observations illuminate potential correlations of demographic and clinical characteristics with nAb responses, a key element for protection from COVID-19, thus informing development and implementation of preventative and therapeutic strategies globally.

**Trial registration:**

ClinicalTrials.gov NCT04403880.

## Abstract 2

Shelly Karuna and co-workers study variations in neutralizing antibody responses after SARS-CoV-2 infection.

## Author summary

**Why was this study done?:**

- To define the immune responses to natural SARS-CoV-2 infection.
- To identify demographic and clinical characteristics associated with patterns of anti-Severe Acute Respiratory Syndrome Coronavirus 2 (SARS-CoV-2) neutralizing antibody (nAb) responses over time.

**What did the researchers do and find?:**

- We enrolled a cohort of individuals in the United States and Peru recovering from SARS-CoV-2 infection, completed questionnaires, and collected blood and nasal samples.
- We documented their demographics, medical history, and SARS-CoV-2–related history and measured levels of nAbs to SARS-CoV-2 spike protein.
- We found that nAb levels were higher in individuals recovering from severe Coronavirus Disease 2019 (COVID-19), >55 years old, with diabetes, and assigned male sex at birth, and lower in people with hypertension.

**What do these findings mean?:**

- nAb responses varied over time by demographic (e.g., age and sex assigned at birth) and clinical (e.g., diabetes and hypertension) factors, which should be considered in future SARS-CoV-2 immunologic analyses.
- Correlating SARS-CoV-2 antibody responses with clinical outcomes will help inform future treatment and prevention strategies.

## Introduction

Since its emergence in late 2019, Severe Acute Respiratory Syndrome Coronavirus 2 (SARS-CoV-2), the etiologic agent of Coronavirus Disease 2019 (COVID-19) [1–4], has claimed millions of lives and persists at pandemic proportions. This global threat has propelled efforts to rapidly develop, assess, and implement effective vaccines and antiviral drugs, including monoclonal antibodies (mAbs), for treatment and prevention. Central to these efforts is an understanding of the neutralizing antibody (nAb) response to SARS-CoV-2 and the determinants of that response. nAbs are of intense interest because their passive transfer protects against SARS-CoV-2 in various animal models [5–14], and their elicitation by vaccines correlates with protection in nonhuman primates [15]. Recently, an analysis of correlates of COVID-19 protection in the Phase 3 COVE Moderna mRNA-1273 vaccine trial confirmed preliminary analyses suggesting that nAbs correlate with vaccine efficacy in humans as well [16–18]. Additionally, early treatment with neutralizing mAbs has been shown to reduce viral loads and decrease disease severity in SARS-CoV-2–infected individuals [19,20]. This early progress may be attenuated by emerging variants’ potential escape from neutralization-mediated vaccine and mAb efficacy [21,22]. Thus, profiling nAb responses following natural SARS-CoV-2 infection of varying severity and host characteristics can inform efforts to characterize immunologic profiles associated with favorable clinical outcomes, as well as those correlated with vaccine-induced protection, the escape potential of emerging variants, the susceptibility of individuals to reinfection, and the potential of transmission of infection.

nAbs target the SARS-CoV-2 spike (S) protein that coats the virus surface and binds angiotensin-converting enzyme 2 (ACE2) as a receptor to gain entry into host cells [23,24]. Studies of isolated mAbs show that nAbs from COVID-19–convalescent individuals mainly target nonoverlapping epitopes in the S receptor binding domain (RBD) [1,14,25–29]. Similar RBD nAb specificities are induced in people who receive mRNA vaccines against COVID-19 [30]. Other, less abundant nAbs target a single supersite in the S N-terminal domain (NTD) [31–33].

The serum nAb response in COVID-19–convalescent persons is highly variable, exhibiting approximately 1,000-fold range of titers among samples collected from recovering patients, and with approximately 20% to 30% of people mounting very weak or undetectable responses [34–43]. When detected, nAbs are often present within approximately 8 days of symptom onset [37,39,43] and peak approximately 20 to 40 days later [37,41–46]. Following the peak, nAb titers decline with a half-life of approximately 8 weeks [34,40,47–53] and often stabilize at low levels after several months [44]. Notably, anti-S-specific memory B-cell frequency and somatic hypermutation increase for up to 6 months despite declining nAb titers [49,50,54–56]. Most studies demonstrate that stronger nAb responses are associated with greater disease severity [35,37,38,41,43,44,47,48,51,52,57–59]. nAb responses also appear to be modestly stronger in individuals assigned male sex at birth [35,38,47,53] and in older individuals [35,38,41,42,47].

A number of underlying medical conditions are associated with a greater risk of severe disease and death from COVID-19 [60–62]; however, little is known about the potential impact of these comorbidities on SARS-CoV-2–specific nAb responses. We investigated nAb profiles and associations with demographic and clinical variables in a globally diverse cohort of several hundred individuals from a median of 2 months after the onset of their infection or illness.

## Materials and methods

### Study cohort

From May through October 2020, 329 HIV-uninfected participants from the United States (*n* = 167) and Peru (*n* = 162) with a history of SARS-CoV-2 infection provided written informed consent and were enrolled into the HVTN 405/HPTN 1901 observational cohort study (NCT04403880) led by the COVID-19 Prevention Network (CoVPN). Participants were enrolled at 26 clinical research sites (CRS) in Peru and the US (**S1 Text**). Complete eligibility criteria are listed in **S1 Text**.

Participants were stratified by symptomatic and asymptomatic disease, inpatient or outpatient care requirement, and age (18 to 55 or >55 years of age). Asymptomatic individuals (*n* = 65, 19.8%) were eligible to enroll 2 to 10 weeks after the most recent test at which SARS-CoV-2 was detected; symptomatic individuals (*n* = 264, 80.2%) were eligible to enroll 1 to 8 weeks after resolution of COVID-19.

Diagnosis was defined for asymptomatic individuals as the first test (direct viral detection, i.e., PCR or antigen) at which SARS-CoV-2 was detected and for symptomatic individuals as the first test at which SARS-CoV-2 was detected or the first day of symptoms, whichever occurred first. These 329 participants were included in analyses of nAb responses at the enrollment visit (V1). Of these 329 participants, 186 (57%) (94 in Peru [58%] and 92 in the US [55%]) also provided serum for nAb assessment as of the data cutoff in October 2020 at a follow-up visit (V2) conducted approximately 2 months post-enrollment.

The study consisted of a single required visit, with optional and ongoing follow-up at 2, 4, and 12 months post-enrollment. Participants provided nasopharyngeal and blood samples and demographic and medical information, including the date of confirmation of SARS-CoV-2 infection by direct viral detection testing (i.e., molecular or antigen test) and, if applicable, details regarding COVID-19 symptoms. Institutional Review Board (IRB) approval was granted by a Central IRB and, as applicable, by individual CRS’ IRBs.

### Laboratory methods

nAbs against SARS-CoV-2 were measured in a formally validated assay as a function of reductions in luciferase (Luc) reporter gene expression after a single round of infection with SARS-CoV-2.D614G Spike-pseudotyped virus in 293T/ACE2 cells as described [63]. Briefly, Spike-pseudotyped virus was prepared by transfection in 293T cells using a lentivirus backbone vector and a firefly Luc reporter plasmid. A pretitrated dose of pseudovirus was incubated with 8 serial 5-fold dilutions of serum samples in duplicate in 96-well flat-bottom poly-L-lysine–coated culture plates for 1 hour at 37°C prior to adding 293T/ACE2 cells. One set of 8 wells received cells + virus (virus control) and another set of 8 wells received cells only (background control). Luminescence was measured after 66 to 72 hours of incubation using Promega 1X lysis buffer and Bright-Glo Luc reagent. Neutralization titers are the inhibitory dilution of serum samples at which relative Luc units (RLUs) were reduced by either 50% (ID50) or 80% (ID80) compared to virus control wells after subtraction of background RLUs. Serum samples were heat inactivated for 30 minutes at 56°C prior to assay. The ID50 represents a 2-fold reduction in viral infectivity, and the more stringent ID80 represents a 5-fold reduction in viral infectivity. Both ID50 and ID80 are reported for this cohort, as it remains unknown which titer may be more clinically meaningful or what nAb titer threshold may be necessary to provide protection from SARS-CoV-2 infection or COVID-19.

### Statistical methods

#### Demographics and preexisting medical history, associations with nAb responses

Participants’ characteristics at enrollment were summarized in total and by region (the US and Peru) and compared between the US and Peru using chi-squared tests for categorical variables and *t* tests for continuous variables. Multivariable regressions were used to study noncausal associations between nAb responses at enrollment (V1) with COVID-19 severity, medical history, demographics [age (>55 versus 18 to 55), sex assigned at birth, race/ethnicity (non-Hispanic Black, Hispanic/Latino, Others versus non-Hispanic White)], and body mass index (BMI) (≥30 versus <30) adjusting for region (Peru versus the US) and days post-SARS-CoV-2 diagnosis, simultaneously in the same model to control for any potential confounding factors. The multivariable regressions adjusting for all potential confounders in the same model were conducted as the primary analyses to avoid “underadjustment.” However, to address the possibility of “overadjustment” for some associations, and appreciating the knowledge gaps regarding the myriad mechanisms through which the factors we studied may directly and/or indirectly (e.g., through mediators) impact the nAb response, we also evaluated associations adjusting for a minimum set of confounders related only to the cohort study design [64,65]. These included the following: disease severity and age, because enrollment was stratified by these factors; region, because enrollment was allocated by region; and days since SARS-CoV-2 diagnosis, as eligibility criteria specified a window of post-diagnosis eligibility for participants convalescing from asymptomatic and symptomatic illness.

COVID-19 severity was defined by asymptomatic illness, symptomatic outpatient illness, or illness requiring hospitalization; in some supplemental models, as noted, illness requiring hospitalization was further subdivided by history of supplemental oxygen or intubation requirement. In the more adjusted, primary multivariable model, the associations of age and sex assigned at birth with nAb, stratified by COVID-19 severity, and the association of COVID-19 severity, stratified by age and sex assigned at birth, were also assessed, including the interaction of COVID-19 severity with age groups and sex assigned at birth, to test those stratified associations.

Logistic regressions were used for associations with response rates and log-linear regressions were used for associations with titers. Adjusted *p*-values were calculated for multiple testing correction using the Benjamini and Hochberg method [66] to control for a false discovery rate. Adjusted *p*-values were called *q*-values.

Odds ratios (ORs), 95% confidence intervals (CIs), *p*-values, and *q*-values were reported for response rate associations. Geometric mean ratios (GMRs), 95% CIs, *p*-values, and *q*-values were reported for response titer associations. A detectable nAb response is defined as an ID50 titer >20, the limit of detection (LOD of 20); any titers below this were set to be half of LOD [66].

As a primary aim of this study was to characterize the anti-Spike nAb responses in this observational, convalescent cohort, all analyses focus on estimating noncausal associations, thus generating hypotheses about potential drivers of nAb responses.

#### Corticosteroids

nAb response rates and titers were compared between participants taking and not taking corticosteroids stratified by COVID-19 severity and adjusting for age, sex assigned at birth, BMI, diabetes, hypertension, and days since SARS-CoV-2 diagnosis using the multivariable model. Sixty participants in this cohort reported using corticosteroids during their illness; one was asymptomatic. Therefore, we restricted the association of corticosteroids with nAb response to a subset of participants who were symptomatic (outpatients or hospitalized). As all symptomatic outpatients who reported having taken corticosteroids had detectable nAb titers, Firth logistic regression [67] was used for the association with nAb response rate.

#### Antihypertensive medications

nAb response rates and titers were compared between participants with hypertension taking and not taking ACE inhibitors and/or angiotensin-receptor blocker (ARB) medications, adjusting for COVID-19 severity, age, sex assigned at birth, diabetes, and days since SARS-CoV-2 diagnosis using the multivariable model.

#### Nonresponders

The characteristics of nonresponders (i.e., individuals with no detectable ID50 or ID80 nAb titer) were tabulated and compared with the characteristics of responders using a similar multivariable model that was used assessing demographic and preexisting medical conditions’ associations with nAb response rate, but with nonresponse as the outcome.

#### Prolonged viral shedding

Prolonged viral shedding was defined as a report of 2 tests detecting virus at least 21 days apart. Thirty-four participants in this cohort exhibited prolonged viral shedding; one was asymptomatic. Therefore, we restricted the association of prolonged viral shedding with nAb response to a subset of participants who were symptomatic (outpatients or hospitalized) to reduce potential confounding by including asymptomatic participants who very rarely exhibited prolonged viral shedding. The association was evaluated adjusting for COVID-19 severity, age, sex assigned at birth, BMI, region, and days since SARS-CoV-2 diagnosis using the multivariable model. This analysis was the only one not prespecified.

#### nAb responses over time

For participants with nAb data at V1 and V2, we assessed associations of COVID-19 severity, medical and smoking history, demographics, and days since SARS-CoV-2 diagnosis at V1 with nAb titer at V2 (adjusting for titer at V1), or with fold-change from V1 to V2 simultaneously in a multivariable log-linear regression.

Generalized additive models (GAMs) [68] were also applied to nonparametrically assess the association of several continuous variables (such as age, BMI, and days since SARS-CoV-2 diagnosis) on nAb titer at V1, adjusting for COVID-19 severity, sex assigned at birth, race/ethnicity, region, and medical and smoking history. Cubic smoothing splines were used for each continuous variable, stratified by COVID-19 severity. Separate models were fit for each continuous variable. The estimated cubic spline smoothing functions and pointwise 95% confidence bands were graphically displayed.

All available nAb titers from both V1 and V2 were used to examine a trajectory of nAb titers across days post-SARS-CoV-2 diagnosis by COVID-19 severity. A generalized additive mixed model (GAMM) was used to fit cubic smoothing splines of days post-SARS-CoV-2 diagnosis accounting for the correlations of nAb responses between 2 visits within participants [69].

All the tests were two-sided. *p*-values ≤ 0.05 and *q*-values ≤ 0.2 were considered statistically significant. A STROBE checklist (**S1 File)** and prespecified analysis plan (**S2 Text)** are found in the supporting information.

## Results

### Race, ethnicity and region, and associations with nAb responses

Overall, 54% of the cohort was Hispanic; 9% of the US cohort identified as Hispanic. About 52% of participants identified as Other race (98.8% of Peru participants and 6% of the US participants); 34% White (1.2% of Peru participants, 65.3% of the US participants); 11% Black (21.6% of the US participants), and 3.6% Asian (7.2% of the US participants) (**Table 1**). A greater proportion of participants from Peru than from the US had been hospitalized for COVID-19 (48.8% versus 31.7%); fewer participants from Peru were asymptomatic (17.3 versus 22.2%) (**Table 1**). SARS-CoV-2 testing was not available to asymptomatic individuals in Peru during the time of this study’s conduct; thus, most Peruvian participants who met the eligibility requirement of having had a prior test detecting SARS-CoV-2 also had a history of symptomatic infection. There were no significant differences in nAb response rates or titers by race or ethnicity or between the 2 regions after adjusting for other factors in the US and Peru combined cohort (**Table 2**) or after adjusting for only study design–related variables (disease severity, age, region, and days since SARS-CoV-2 diagnosis) (**Table A in S1 Data**). Similar associations were observed in the US and Peru cohorts separately (**Tables A and B in S1 Data**).

**Table 1:** Summary of participants characteristics at enrollment.

Characteristic	Total (*n* = 329)	Peru (*n* = 162)	US (*n* = 167)	*p*-value
Age				
Mean (SD)	47.8 (15.16)	47.7 (14.2)	48 (16.07)	0.865
Median (IQR)	48 (35, 60)	46.5 (36, 60)	50 (34, 60)	
Min–Max	18–86	18–80	18–86	
18–55	201 (61.1%)	104 (64.2%)	97 (58.1%)	0.306
>55	128 (38.9%)	58 (35.8%)	70 (41.9%)	
Assigned sex at birth				
Female	163 (49.5%)	74 (45.7%)	89 (53.3%)	0.204
Male	166 (50.5%)	88 (54.3%)	78 (46.7%)	
Race				
White	111 (33.7%)	2 (1.2%)	109 (65.3%)	<0.001
Black	36 (10.9%)	0 (0%)	36 (21.6%)	
Asian	12 (3.6%)	0 (0%)	12 (7.2%)	
Others	170 (51.7%)	160 (98.8%)	10 (6.0%)	
Ethnicity				
Hispanic or Latino	177 (53.8%)	162 (100%)	15 (9%)	<0.001
Not Hispanic or Latino	152 (46.2%)	0	152 (91%)	
BMI				
Mean (SD)	29 (6.11)	28.1 (4.49)	29.9 (7.27)	0.010
Median (IQR)	27.7 (24.6, 31.6)	27.3 (25, 30.9)	28.9 (24.4, 33.5)	
Min–Max	15.6–55	15.6–41.4	19.3–55	
<30	206 (62.6%)	112 (69.1%)	94 (56.3%)	0.022
≥30	123 (37.4%)	50 (30.9%)	73 (43.7%)	
COVID-19 severity at enrollment				
Asymptomatic	65 (19.8%)	28 (17.3%)	37 (22.2%)	0.007*
Symptomatic outpatient	132 (40.1%)	55 (34%)	77 (46.1%)	
Hospitalized	132 (40.1%)	79 (48.8%)	53 (31.7%)	
Hospitalized, no supp oxygen	23 (7%)	18 (11.1%)	5 (3%)	
Hospitalized, oxygen, no intubation/ICU	62 (18.8%)	33 (20.4%)	29 (17.4%)	
Hospitalized, intubation/ICU	47 (14.3%)	28 (17.3%)	19 (11.4%)	
Hypertension	75 (22.8%)	24 (14.8%)	51 (30.5%)	0.001
COPD/emphysema/asthma	35 (10.6%)	9 (5.6%)	26 (15.6%)	0.006
Diabetes	42 (12.8%)	17 (10.5%)	25 (15%)	0.293
Currently smoke cigarettes or marijuana	27 (8.2%)	7 (4.3%)	20 (12%)	0.020
Ever smoked cigarettes or marijuana	141 (42.9%)	49 (30.2%)	92 (55.1%)	<0.001
Prolonged viral shedding	34 (10.3%)	11 (6.8%)	23 (13.8%)	0.058
Days since SARS-CoV-2 diagnosis				
Mean (SD)	52.2 (18.61)	45.6 (16.05)	58.7 (18.69)	<0.001
Median (IQR)	53 (38, 67)	44.5 (35, 57)	60 (47, 71)	
Min–Max	13–120	13–89	13–120	
<28	28 (8.5%)	21 (13%)	7 (4.2%)	<0.001
29–41	70 (21.3%)	46 (28.4%)	24 (14.4%)	
42–55	86 (26.1%)	49 (30.2%)	37 (22.2%)	
56+	145 (44.1%)	46 (28.4%)	99 (59.3%)	

*p-value for the comparison of 3 severity groups, not including subgroups of hospitalized individuals.

BMI, body mass index; COPD, chronic obstructive pulmonary disease; COVID-19, Coronavirus Disease 2019; ICU, intensive care unit; SARS-CoV-2, Severe Acute Respiratory Syndrome Coronavirus 2.

**Table 2:** Associations of COVID-19 severity, medical history, demographics, and days since SARS-CoV-2 diagnosis with nAb responses among the convalescent participants in Americas (*n* = 329).

	Response Rate				Titer			
	OR^+^	95% CI	*p*-value	*q*-value	GMR*	95% CI	*p*-value	*q*-value
**ID50**								
COVID-19 Severity (Overall)	**-**	**-**	**<0.001**		**-**	**-**	**<0.001**	
Symp outpatient vs Asymp	**3.74**	**[1.45, 9.99]**	**0.007**	**0.031**	**5.66**	**[3.17, 10.11]**	**<0.001**	**<0.001**
Hospital vs Symp outpatient	**4.39**	**[1.5, 14.4]**	**0.009**	**0.031**	**3.95**	**[2.48, 6.29]**	**<0.001**	**<0.001**
Diabetes	3.98	[1.08, 19.05]	0.056	0.145	**2.36**	**[1.3, 4.28]**	**0.005**	**0.015**
Hypertension	**0.16**	**[0.06, 0.42]**	**<0.001**	**0.003**	**0.5**	**[0.31, 0.82]**	**0.006**	**0.015**
Age (>55 vs 18–55)	**3.46**	**[1.42, 9.33]**	**0.009**	**0.031**	**2.33**	**[1.54, 3.52]**	**<0.001**	**<0.001**
Sex at Birth (Male vs Female)	1.23	[0.56, 2.72]	0.61	0.802	**1.52**	**[1.02, 2.27]**	**0.042**	**0.092**
COPD/Emphysema/Asthma	1.17	[0.37, 4.25]	0.802	0.802	0.87	[0.46, 1.63]	0.663	0.784
Current Cigarettes/Mari Smoker	1.21	[0.38, 4.17]	0.754	0.802	0.97	[0.46, 2.04]	0.94	0.94
Ever Cigarettes/Mari Smoker	0.57	[0.25, 1.3]	0.182	0.338	0.81	[0.53, 1.24]	0.33	0.536
Non-His Black vs White	0.63	[0.2, 1.99]	0.426	0.693	0.92	[0.45, 1.88]	0.824	0.892
His/Latino vs Non-His White	4.51	[0.73, 89.28]	0.178	0.338	1.45	[0.57, 3.71]	0.438	0.62
Others vs Non-His White	1.39	[0.27, 10.85]	0.716	0.802	1.44	[0.53, 3.97]	0.477	0.62
BMI (≥30 vs <30)	1.21	[0.56, 2.68]	0.638	0.802	1.37	[0.92, 2.04]	0.123	0.229
**ID80**								
COVID-19 Severity (Overall)	**-**	**-**	**<0.001**		**-**	**-**	**<0.001**	
Symp outpatient vs Asymp	**6.72**	**[2.91, 16.32]**	**<0.001**	**<0.001**	**4.14**	**[2.68, 6.39]**	**<0.001**	**<0.001**
Hospital vs Symp outpatient	**5.06**	**[2.09, 13.28]**	**0.001**	**0.003**	**3.79**	**[2.68, 5.38]**	**<0.001**	**<0.001**
Diabetes	**3.35**	**[1.09, 12.05]**	**0.046**	**0.117**	**1.81**	**[1.16, 2.82]**	**0.01**	**0.025**
Hypertension	**0.32**	**[0.14, 0.75]**	**0.01**	**0.031**	0.75	[0.52, 1.08]	0.123	0.229
Age (>55 vs 18–55)	**3.13**	**[1.5, 6.89]**	**0.003**	**0.014**	**1.98**	**[1.45, 2.69]**	**<0.001**	**<0.001**
Sex at Birth (Male vs Female)	1.48	[0.76, 2.93]	0.249	0.36	**1.55**	**[1.15, 2.1]**	**0.004**	**0.014**
COPD/Emphysema/Asthma	0.86	[0.33, 2.32]	0.754	0.891	1.03	[0.64, 1.65]	0.898	0.9
Current Cigarettes/Mari Smoker	2.2	[0.72, 7.26]	0.179	0.29	1.04	[0.59, 1.81]	0.9	0.9
Ever Cigarettes/Mari Smoker	0.62	[0.31, 1.24]	0.176	0.29	0.95	[0.69, 1.3]	0.75	0.9
Non-His Black vs White	0.96	[0.34, 2.78]	0.94	0.988	1.19	[0.7, 2.03]	0.522	0.849
His/Latino vs Non-His White	1.46	[0.41, 5.73]	0.568	0.739	1.12	[0.55, 2.26]	0.757	0.9
Others vs Non-His White	1.01	[0.23, 4.87]	0.988	0.988	1.11	[0.52, 2.36]	0.794	0.9
BMI (≥30 vs <30)	**1.97**	**[1, 4]**	**0.054**	**0.117**	**1.44**	**[1.07, 1.94]**	**0.018**	**0.039**

^+^Odds ratio.

*Geometric mean ratio.

asymp, asymptomatic; BMI, body mass index; CI, confidence interval; COPD, chronic obstructive pulmonary disease; COVID-19, Coronavirus Disease 2019; His, Hispanic; hospital, hospitalized; mari, marijuana; nAb, neutralizing antibody; SARS-CoV-2, Severe Acute Respiratory Syndrome Coronavirus 2; Symp, symptomatic.

Region and days since SARS-CoV-2 diagnosis were adjusted in the model.

Bold = significant.

### Age and associations with nAb responses

The mean age of the cohort was 48 years (range 18 to 86; 39% age >55 years old [yo]) and did not differ significantly between the US and Peru regional cohorts (mean 48 versus 48, *p* = 0.87; 42% versus 36% age >55, *p* = 0.31) (**Table 1**).

**Fig 1:**
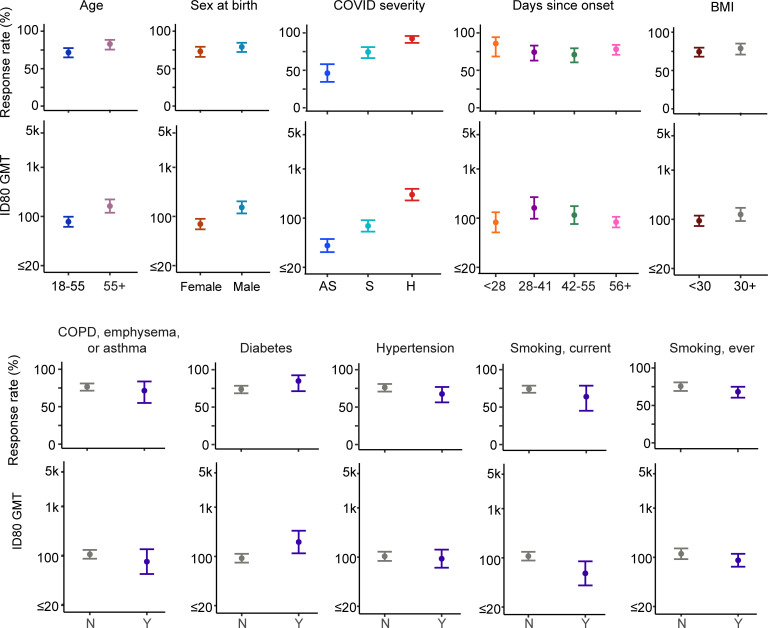
ID50 GMT at enrollment visit and 95% CI by enrollment group, severity, medical history, and days since SARS-CoV-2 diagnosis. CI, confidence interval; COPD, chronic obstructive pulmonary disease; GMT, geometric mean titer; SARS-CoV-2, Severe Acute Respiratory Syndrome Coronavirus 2.

In unadjusted analyses, older adults, defined as those >55 yo, exhibited higher nAb titers than adults ≤55 yo, with geometric mean titers (GMTs) of 829 versus 362 for ID50 and 162 versus 78 for ID80, respectively **(Fig 1, Fig A in S2 Data, Table C in S1 Data)**. After adjusting for other potential contributing factors, including disease severity, medical and smoking history, sex assigned at birth, race/ethnicity, BMI, region, and days since SARS-CoV-2 diagnosis in one model or adjusting for other design-specific variables only (i.e., disease severity, region, and days since SARS-CoV-2 diagnosis) in another model, nAb GMT remained at least twice as high among adults >55 yo compared to those ≤55 yo (ID50, *p* < 0.001; ID80, *p* < 0.001) (**Table 2, Table A in S1 Data**). Similarly, older adults were more likely to exhibit a nAb response than younger adults: 89% versus 83% for ID50 and 83% versus 72% for ID80 (**Fig 1, Fig A in S2 Data, Table D in S1 Data**). The odds of a detectable nAb response was over 3 times higher among older compared to younger adults after adjusting for the other potential contributing factors noted above (ID50, *p* = 0.009, *q* = 0.031; ID80, *p* = 0.003, *q* = 0.014) (**Table 2**); this difference remained but was somewhat diminished in the model adjusting only for the study design-specific factors of disease severity, region, and days since SARS-CoV-2 diagnosis (ID50, OR = 1.81, *p* = 0.109; ID80, OR = 2.29, *p* = 0.011, *q* = 0.049) (**Table A in S1 Data**). Similar trends between age and nAb responses were also observed in the US and Peru cohorts independently (**Tables A and B in S1 Data**).

There were no differences in nAb response rates (ID50: OR = 1.35, *p* = 0.71; ID80: OR = 0.85, *p* = 0.82) or titers (ID50: GMR = 1.16, *p* = 0.76; and ID80: GMR = 1.01, *p* = 0.98) by age within the asymptomatic subgroup. However, compared to younger adults in the same severity category, nAb response rates were higher in symptomatic outpatient older adults (ID50: OR = 5.96, *p* = 0.01, *q* = 0.05; ID80: OR = 4.74, *p* = 0.004, *q* = 0.011), as were nAb titers (ID50: GMR = 3.94, *p* < 0.001, *q* < 0.001; ID80: GMR = 2.74, *p* < 0.001, *q* < 0.001), and the same pattern was observed in hospitalized older adults compared to hospitalized younger adults (ID50: OR = 2.98, *p* = 0.26 and GMR = 1.72, *p* = 0.08; ID80: OR = 6.32, *p* = 0.041, *q* = 0.057 and GMR = 1.8, *p* = 0.012, *q* = 0.015; **Table E in S1 Data**). The associations of nAb responses with age (in years) stratified by COVID-19 severity were estimated using the GAM, adjusting for other potential contributing factors, and support these observations (**Fig B in S2 Data**). Among asymptomatic participants, nAb titers did not significantly differ by age, while nAb titers among symptomatic outpatients and hospitalized participants increased with age after approximately 50 yo (**Fig B in S2 Data**).

### Sex assigned at birth and associations with nAb responses

About 50% of the cohort were assigned female sex at birth, and this proportion did not differ significantly between the 2 regional cohorts (53% in the US versus 46% in Peru, *p* = 0.2) (**Table 1**). Participants assigned male sex at birth exhibited 2-fold higher nAb titers than those assigned female sex at birth with GMT of 701 versus 354 for ID50 and 152 versus 70 for ID80 (**Table C in S1 Data**); titers were over 1.5 times higher after adjusting for other factors (ID50, *p* = 0.042, *q* = 0.092; ID80, *p* = 0.004, *q* = 0.014; **Table 2**). Similar increases were observed in the model adjusting for study design–specific variables only, though were not significant at ID50 (ID50 GMR: 1.36, *p* = 0.121, *q* = 0.225; ID80 GMR: 1.51, *p* = 0.006, *q* = 0.016) (**Table A in S1 Data**). However, those assigned male sex at birth did not exhibit significantly higher rates of nAb responses than those assigned female sex at birth (ID50: 85% versus 86%; ID80: 79% versus 73%) (**Table 2, Tables A and D in S1 Data**).

As observed with nAb response differences by age, the differences in nAb titers between those assigned male and those assigned female sex at birth were not evident among asymptomatic individuals (ID50: GMR = 0.53, *p* = 0.14; ID80: GMR = 0.84, *p* = 0.6); they were also not evident in symptomatic outpatients (ID50: GMR = 1.53, *p* = 0.18; ID80: GMR = 1.56, *p* = 0.06) but were evident in those who were hospitalized (ID50: GMR = 2.41, *p* = 0.005, *q* = 0.008; ID80: GMR = 2.02, *p* = 0.003, *q* = 0.004) (**Table E in S1 Data)**.

In unadjusted analyses, nAb titers among those assigned male sex at birth peaked later than among those assigned female sex at birth (**Fig C in S2 Data**). Among those who contributed samples at both V1 and, about 2 months later, at V2, titers also declined more quickly among males compared to females (ID50: GMR = 1.4, *p* = 0.051, *q* = 0.24; ID80: GMR = 1.46, *p* = 0.012, *q* = 0.056) (**Table F in S1 Data**).

### Severity of SARS-CoV-2 infection and associations with nAb responses

In this cohort overall, 19.8% (*n* = 65) of participants were asymptomatic (17.3% in Peru; 22% in the US); 40.1% (*n* = 132) were symptomatic but did not require hospitalization (34% in Peru, 46.1% in the US); and 40% (*n* = 132) had been hospitalized (48.8% in Peru, 31.8% in the US). The distribution of disease severity differed significantly across the 2 regions (*p* = 0.007; **Table 1**).

nAb titers were higher among participants with a history of severe illness (*p* < 0.001; **Table 2**); the ID50 and ID80 GMT, respectively, at enrollment were 101 and 28 among asymptomatic individuals, 356 and 68 for symptomatic outpatient individuals, and 1,544 and 301 for hospitalized individuals (**Table C in S1 Data**). The ID50 and ID80 GMT were higher among symptomatic outpatients than asymptomatic participants (ID50: GMR = 5.66, *p* < 0.001, *q* < 0.001; ID80: GMR = 4.14, *p* < 0.001, *q* < 0.001) and higher among hospitalized participants than symptomatic outpatients (ID50: GMR = 3.95, *p* < 0.001, *q* < 0.001; ID80: GMR = 3.79, *p* < 0.001, *q* < 0.001) after adjusting for medical and smoking history, demographics, and days since SARS-CoV-2 diagnosis (**Table 2**). Similar associations were observed in the models adjusting for only other design-specific variables (age, region, and days since SARS-CoV-2 diagnosis) (**Table A in S1 Data**). While the highest titers were observed in participants who required hospitalization, titers did not vary significantly among those hospitalized when compared by oxygen requirement (e.g., no supplemental O_2_, supplemental O_2_ but no intubation, or intubation) (**Table G in S1 Data**).

nAb responses were detected at enrollment in 66% of asymptomatic individuals (46% for ID80), 86% of symptomatic outpatients (74% for ID80), and 95% of hospitalized individuals (92% for ID80) (**Fig 1, Fig A in S2 Data, Table D in S1 Data**). The odds of having a detectable ID50 titer after adjusting for other factors were 3.7 times higher for symptomatic outpatients compared to asymptomatic individuals (*p* = 0.007, *q* = 0.031) and 6.7 times higher for ID80 titers (*p* < 0.001, *q* < 0.001). Similarly, the odds of having an ID50 titer were 4.4 times higher for hospitalized individuals compared with symptomatic outpatients (*p* = 0.009, *q* = 0.031) and 5 times higher for ID80 titers (*p* = 0.001, *q* = 0.003) (**Table 2**). Similar associations of disease severity with nAb responses were observed in the less adjusted model (**Table A in S1 Data**) and in the US and Peru cohorts separately (**Tables A and B in S1 Data**). The differences between severity groups were observed within both age groups and within both sex assignments, but the differences between symptomatic outpatient and asymptomatic individuals were much more evident in older adults and those assigned male sex at birth (**Table E in S1 Data**).

More severely ill individuals also differed from asymptomatic individuals with respect to the COVID-related treatments they received. Most notably, 60 participants reported receiving corticosteroid treatment, of whom 1 had been asymptomatic, 10 had been symptomatic outpatients, and 49 had been hospitalized with COVID-19. A subset analysis within the symptomatic outpatient and hospitalized severity groups identified no statistically significant association of corticosteroid use with nAb response rates in either severity group, and no statistically significant association of nAb titers with corticosteroid use among individuals who were hospitalized with COVID-19. Symptomatic outpatients who reported taking corticosteroids exhibited higher ID80 titers (ID80: GMR = 2.28, *p* = 0.054; ID50 GMR = 2.65, *p* = 0.079; **Table H in S1 Data**).

### nAb responses over time

The mean (median) time from SARS-CoV-2 diagnosis to enrollment was 52 (53) days, with a range of 13 to 120 days. Participants in the US enrolled later post-diagnosis on average than those in Peru (59 versus 46 days, *p* < 0.001), with 82% in the US versus 59% in Peru enrolling 42 or more days from diagnosis (*p* < 0.001; **Table 1**).

**Fig 2:**
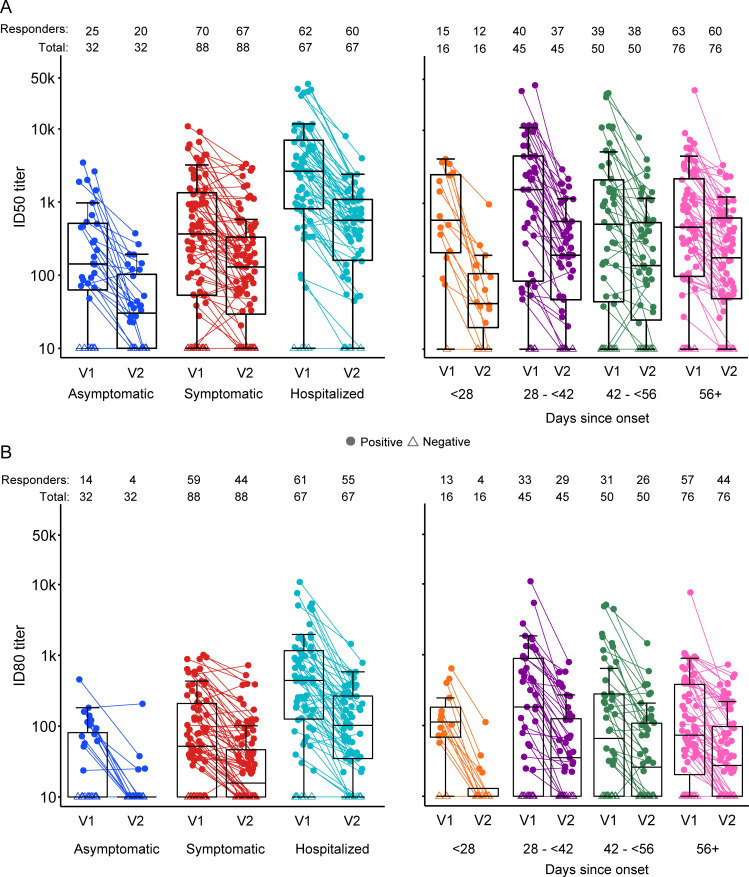
nAb response titers (ID50/ID80) at V1 (enrollment visit) and V2 (2 months after V1) by COVID-19 severity and days since SARS-CoV-2 diagnosis at V1 among participants (*n* = 186) with data at both time points. Gray lines connect the nAb titer at V1 and V2 within participants. A. ID50. B. ID80. COVID-19, Coronavirus Disease 2019; nAb, neutralizing antibody; SARS-CoV-2, Severe Acute Respiratory Syndrome Coronavirus 2.

Differences in nAb titers and response rates by disease severity persisted 2 months post-enrollment, even as ID50 (ID80) GMT among asymptomatic, symptomatic outpatient, and hospitalized individuals declined 4.4 (2.2) times, 2.4 (2.3) times, and 5 (4) times, respectively (**Fig 2, Table I in S1 Data**); these differences in nAb titers at V2 by disease severity were not statistically significant after adjusting for nAb titers at the enrollment visit (V1) and other factors, except for the difference in ID50 titers between symptomatic outpatient and asymptomatic individuals (GMR = 1.67, *p* = 0.012, *q* = 0.087) (**Table 3**). Of note, the nAb titer at V1 among hospitalized participants was particularly high, resulting in a faster decline in titers from V1 to V2 for hospitalized compared to asymptomatic (ID50: GMR = 1.41, *p* = 0.20; ID80: GMR = 2.19, *p* = 0.001, *q* = 0.014) and symptomatic outpatients (ID50: GMR = 1.57, *p* = 0.024, *q* = 0.168; ID80: GMR = 1.66, *p* = 0.004, *q* = 0.028) (**Table F in S1 Data**).

**Table 3:** Associations of COVID-19 severity, medical history, demographics, days since the first SARS-CoV-2 infection evidence, and ID50/ID80 titer at V1 with titer at V2 among participants with data available at both time points.

	ID50				ID80			
	GMR	95% CI	*p*-value	*q*-value	GMR	95% CI	*p*-value	*q*-value
COVID-19 Severity (Overall)	-	-	**0.029**		-	-	0.059	
Symp outpatient vs Asymp	**1.67**	**[1.12, 2.49]**	**0.012**	**0.087**	1.3	[0.91, 1.86]	0.154	0.266
Hospital vs Symp outpatient	1.07	[0.77, 1.49]	0.673	0.857	1.1	[0.81, 1.48]	0.54	0.581
Diabetes	1.13	[0.71, 1.81]	0.598	0.838	1.33	[0.88, 2.01]	0.171	0.266
Hypertension	1.28	[0.92, 1.77]	0.143	0.502	1.32	[1, 1.76]	0.055	0.245
Age (>55 vs 18–55)	1.32	[0.98, 1.76]	0.068	0.319	1.07	[0.82, 1.38]	0.625	0.625
Sex at Birth (Male vs Female)	0.86	[0.66, 1.13]	0.273	0.637	0.82	[0.65, 1.05]	0.114	0.266
COPD/Emphysema/Asthma	0.98	[0.64, 1.52]	0.939	0.939	0.82	[0.56, 1.21]	0.319	0.372
Current Cigarettes/Mari Smoker	1.05	[0.65, 1.71]	0.842	0.939	1.35	[0.88, 2.07]	0.169	0.266
Ever Cigarettes/Mari Smoker	0.89	[0.68, 1.17]	0.413	0.722	0.84	[0.66, 1.07]	0.157	0.266
Non-His Black vs White	0.97	[0.51, 1.87]	0.936	0.939	1.71	[0.96, 3.03]	0.07	0.245
His/Latino vs Non-His White	1.31	[0.72, 2.38]	0.383	0.722	1.64	[0.97, 2.77]	0.068	0.245
Others vs Non-His White	0.61	[0.28, 1.34]	0.22	0.617	1.55	[0.77, 3.1]	0.221	0.309
BMI (≥30 vs <30)	1.08	[0.83, 1.4]	0.58	0.838	1.14	[0.91, 1.44]	0.261	0.332
log10 (Titer at V1)	**4.6**	**[3.94, 5.38]**	**<0.001**	**<0.001**	**3.88**	**[3.21, 4.69]**	**<0.001**	**<0.001**

asymp, asymptomatic; BMI, body mass index; CI, confidence interval; COPD, chronic obstructive pulmonary disease; COVID-19, Coronavirus Disease 2019; GMR, geometric mean ratio; His, Hispanic; hospital, hospitalized; mari, marijuana; SARS-CoV-2, Severe Acute Respiratory Syndrome Coronavirus 2; Symp, symptomatic.

Bold = significant.

**Fig 3:**
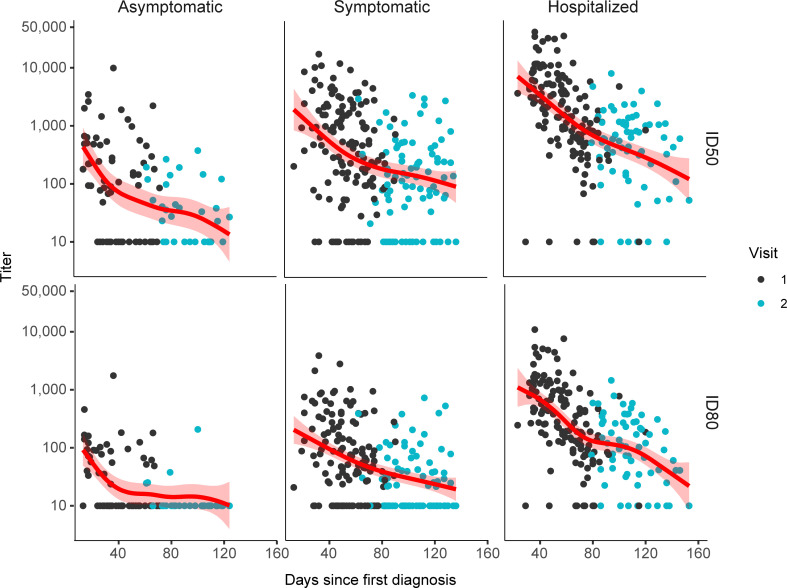
Observations of nAb ID50/ID80 titers against days since SARS-CoV-2 diagnosis from V1 (*n* = 329) and V2 (*n* = 186) stratified by COVID-19 severity (*n* = 65 for V1 and *n* = 32 for V2 in asymptomatic, *n* = 132 for V1 and *n* = 87 for V2 in symptomatic outpatient, and *n* = 132 for V1 and *n* = 67 for V2 in hospitalized). Geometric means (red lines) and 95% confidence bands (pink ribbons) stratified by COVID-19 severity were estimated using GAM with mixed effect to account for the correlations between visits within participants. COVID-19, Coronavirus Disease 2019; GAM, generalized additive model; nAb, neutralizing antibody; SARS-CoV-2, Severe Acute Respiratory Syndrome Coronavirus 2.

ID50 nAb titers declined rapidly early on, before approximately 80 days from SARS-CoV-2 diagnosis, and more slowly after approximately day 80 in all 3 severity groups, as indicated by the slopes of the smoothed estimated GMTs over time since diagnosis (red lines) **(Fig 3)**. Hospitalized individuals had the sharpest decline in ID50 nAb titer before day 80 post-diagnosis. Before day 80, 69% (43%), 86% (73%), and 97% (94%) of ID50 (ID80) nAb titers were detectable in asymptomatic, symptomatic outpatients, and hospitalized individuals, respectively. Rates of detectable nAb ID50 (and ID80) titers dropped to 50% (5%) in asymptomatic individuals, 76% (51%) in symptomatic outpatients, and 88% (81%) in hospitalized patients more than 80 days post-diagnosis. Further investigating the association of days post-SARS-CoV-2 diagnosis on ID50 (ID80) titers at V1 after adjusting all potential contributing factors, we observed that between 20 and 80 days post-diagnosis, ID50 and ID80 titers declined exponentially within each disease severity group (linearly in log-scale) (**Fig D in S2 Data**).

Highlighting the individual variability in nAb response trajectory, 3 participants who had undetectable nAb responses at V1 (42, 45, and 55 days post-diagnosis) had detectable nAb responses at V2. All 3 participants were symptomatic outpatients, assigned female sex at birth, non-Hispanic White from the US, and ages 31, 34, and 48 yo.

### Preexisting medical history and associations with nAb responses

The most commonly reported comorbidities in our cohort were hypertension, diabetes, and chronic lung disease. Overall, 22.8% of participants reported a history of hypertension (14.8% in Peru, 30.5% in the US; *p* = 0.001; **Table 1**); 12.8% reported a history of diabetes (10.5% in Peru, 15% in the US; *p* = 0.29); 10.6% reported a history of chronic lung disease (5.6% in Peru, 15.6% in the US; *p* = 0.006); and 42.9% reported that they had ever smoked cigarettes or marijuana (30.2% in Peru, 55.1% in the US; *p* < 0.001). The mean (median) BMI in this cohort overall was 29 (27.7) with a range of 15.6 to 55; overall, 62.6% had a BMI <30 (69.1% in Peru, 56.3% in the US; *p* = 0.022 for regional difference).

BMI (≥30 versus <30) was not associated with nAb ID50 response rates (OR = 1.21, *p* = 0.64) or ID50 titers (GMR = 1.37, *p* = 0.12) after adjusting for severity, medical and smoking history, age, sex assigned at birth, race/ethnicity, region, and days post-diagnosis (**Table 2**). However, BMI ≥30 was associated with an increased ID80 titer (GMR = 1.44, *p* = 0.018, *q* = 0.039) and a higher ID80 response rate (OR = 1.97, *p* = 0.054, *q* = 0.117) compared with BMI <30 after adjustment. Similar associations were observed in the less adjusted model (**Table A in S1 Data**). The association of BMI with ID80 titers was more evident among hospitalized participants; ID80 titers among hospitalized individuals increased exponentially (linearly on the log-scale) after BMI of approximately 30 (*p* = 0.027) (**Fig E in S2 Data**). However, wide CIs around the trend suggest substantial uncertainty about the magnitude of this trend.

nAb responses were more frequent and at higher titers among participants with diabetes than participants without diabetes, particularly evident in higher titers (ID50: GMR = 2.36, *p* = 0.005, *q* = 0.02; ID80: GMR = 1.81, *p* = 0.01, *q* = 0.03) and higher odds of having a nAb response with a detectable ID80 titer (OR = 3.35, *p* = 0.046, *q* = 0.117) among participants with diabetes compared to participants without diabetes (**Table 2**). The inverse association was observed among those with hypertension, most evident in lower ID50 titers (GMR = 0.5, *p* = 0.006, *q* = 0.02), and lower odds of having a nAb response with a detectable ID50 titer (OR = 0.16, *p* < 0.001, *q* = 0.003) among those with, compared to those without, hypertension after adjusting for COVID-19 severity, demographics, and other preexisting medical history (**Table 2**). There was no statistically significant difference in nAb response rates or in nAb titers between those with hypertension on an ARB and/or ACE inhibitor medication (*n* = 49 of 75 with hypertension) and those with hypertension who were not on these medications (*n* = 26 of 75) (ID50: OR = 0.65, *p* = 0.54; GMR = 0.87, *p* = 0.777; ID80: OR = 0.65, *p* = 0.554; GMR = 0.84, *p* = 0.626; **Table J in S1 Data**).

No significant difference in nAb response rates or titers, either overall or by region, was observed among those reporting either a history of or current smoking tobacco or marijuana (**Table 2, Tables A and B in S1 Data**). Additionally, no significant difference in nAb response rates or titers was observed overall among those reporting a history of chronic lung disease (**Table 2**). However, in our regional analysis, chronic lung disease was associated with lower titers in Peru (ID50: GMR = 0.28, *p* = 0.02, *q* = 0.05; ID80: GMR = 0.36, *p* = 0.02, *q* = 0.05), though not in the US (**Fig F in S2 Data**, **Table B in S1 Data**). In Peru, titers were much higher among those without chronic lung disease (ID50: GMT = 1,096; ID80: GMT = 209) than among those with chronic lung disease (ID50: GMT = 503; ID80: GMT = 117), whereas in the US, the ID50 (ID80) titers among those with and without chronic lung disease were between 200 and 300 (50 to 70) (**Table C in S1 Data**).

### Prolonged viral shedding

One of 65 (1.5%) asymptomatic individuals, 28 of 132 symptomatic outpatients (21%), and 5 of 132 (3.8%) patients with COVID-19 requiring hospitalization had prolonged viral shedding, defined as a report of 2 tests detecting virus at least 21 days apart. In a non-prespecified analysis, prolonged viral shedders in the symptomatic outpatient and hospitalized subgroups had lower nAb titers than those who did not exhibit prolonged viral shedding, but these differences were not statistically significant after adjusting for other potential contributing factors in this cohort (GMR = 0.71, *p* = 0.30 for ID50; GMR = 0.75, *p* = 0.28 for ID80) (**Table 4, Fig G in S2 Data**).

**Table 4:** Comparison of nAb responses between participants with (*n* = 33) and without (*n* = 232) prolonged viral shedding after adjusting for COVID-19 severity, demographics (age, sex assigned at birth, BMI, and region), and days since SARS-CoV-2 diagnosis among symptomatic outpatients and hospitalized individuals in Americas cohort.

	Titer	Response Rate			Response Titer		
		OR	95% CI	*p*-value	GMR	95% CI	*p*-value
Prolonged Viral Shedding (Yes vs No)	ID50	2.26	[0.65, 10.66]	0.239	0.71	[0.36, 1.37]	0.304
	ID80	0.75	[0.29, 2.02]	0.564	0.75	[0.45, 1.25]	0.278

BMI, body mass index; CI, confidence interval; COVID-19, Coronavirus Disease 2019; GMR, geometric mean ratio; nAb, neutralizing antibody; OR, odds ratio; SARS-CoV-2, Severe Acute Respiratory Syndrome Coronavirus 2.

### nAb nonresponders

We observed no detectable ID50 (ID80) nAb responses in 15% (24%) of participants at enrollment (**Table K in S1 Data**). Nonresponders enrolled a median of 51 days from diagnosis with SARS-CoV-2 or COVID-19 and were more likely to be younger (>55 versus 18 to 55 yo; ID50 OR = 0.29, *p* = 0.009, *q* = 0.03; ID80 OR = 0.32, *p* = 0.003, *q* = 0.01) and hypertensive (ID50 OR = 6.19, *p* < 0.001, *q* = 0.003; ID80 OR = 3.09, *p* = 0.01, *q* = 0.03) and less likely to be diabetic (ID50 OR = 0.25, *p* = 0.056, *q* = 0.15; ID80 OR = 0.3, *p* = 0.046, *q* = 0.12) (**Table L in S1 Data**). Nonresponders were also significantly more likely to have milder disease (hospitalized versus symptomatic outpatient ID50 OR = 0.23, *p* = 0.009, *q* = 0.03; ID80 OR = 0.2, *p* = 0.001, *q* = 0.003 and symptomatic outpatient versus asymptomatic ID50 OR = 0.27, *p* = 0.007, *q* = 0.03; ID80 OR = 0.15, *p* < 0.001, *q* < 0.001) (**Table L in S1 Data**). Among the 186 participants evaluated at V2 two months after enrollment, 22% (45%) had no detectable ID50 (ID80) nAb titers at the second time point (**Fig 2**).

## Discussion

An understanding of the role nAbs play in SARS-CoV-2 and COVID-19 may illuminate the path toward a post-pandemic future of protection by active and passive immunization and potentially by herd or community immunity, all of which are likely to depend heavily on nAbs. Moreover, knowing what factors influence nAb responses may inform improved treatments and better clinical outcomes. In our cohort of adults who had recently recovered from PCR-confirmed SARS-CoV-2 infection or COVID-19, being older, assigned male sex at birth, diabetic, having a BMI >30, and a history of more severe illness was associated with higher nAb titers and, except for sex, higher nAb response rates. Hypertension was associated with lower odds of a nAb response and lower titers.

Hypertension is a reported risk factor for poor COVID-19 outcomes [60–62], and among participants with hypertension, we observed fewer nAb responders and lower titers among responders after controlling for potential confounders such as age, diabetes, and obesity. As nAbs promote viral clearance for recovery, the poorer outcomes seen among those with hypertension could be related to these weaker nAb responses. The underlying explanation for relatively low nAb responses and poorer clinical outcome among hypertensives is not known. One hypothesis postulates that ACE inhibitors and/or ARBs, taken by approximately 65% of our participants with hypertension, may mediate changes in baseline inflammation or in ACE2 receptor expression or affinity through the renin-angiotensin-aldosterone system [70]. Large meta-analyses and observational studies have not confirmed this hypothesis [70–72], but if ACE2 or related receptors are down-regulated and viral replication is thus initially limited, lower nAb responses may reflect less early antigen stimulation. We saw no statistically significant difference in nAb responses between hypertensive individuals who did or did not report ARB or ACE inhibitor use, but few participants were not on these medications and the directionality of the nonsignificant differences we observed—lower odds of a nAb response and lower titers among those on ARBs or ACE inhibitors—are consistent with this hypothesis.

Antigen stimulation may also help explain the well-documented association between high nAb responses and disease severity [35,37,38,43,44,47,48,51,52,57–59,73]. Others have also reported similar associations with age and sex assigned at birth [35,38,41,42,47,53], as well as diabetes [47] and BMI [74]. In fact, older, male, diabetic, obese, and more severely ill individuals have suffered the worst COVID-19 outcomes [60–62]. The high nAb responses we observed in these subgroups may reflect the magnitude and duration of viral load, resulting in more antigen persisting longer that stimulates higher nAb titers and, in most subgroups, higher response rates. Most of these subgroups exhibit characteristics that may facilitate high levels or extended periods of antigen stimulation, such as high expression of ACE2 receptors [60,61,75].

However, antigen exposure alone does not fully explain high nAb titers and their apparent association with poor clinical outcome. In an interim analysis of 275 outpatients within 7 days of COVID-19 symptom onset, higher viral loads correlated with lower baseline nAb titers [20]. Furthermore, we observed no significant change in nAb response rates and titers in people with prolonged viral shedding. Yet, older adults, patients with diabetes, and obese individuals often exhibit elevated baseline inflammation, impaired early immune responses, and other immunologic factors that likely work in tandem with virologic factors to explain comparatively poor clinical outcomes in spite of high post-convalescent nAb titers [60,61,75]. Suboptimal or delayed immune responses may contribute to worse clinical outcomes in spite of the ultimate development of higher nAb responses and titers—a response that may well be “too late” to help many in these subgroups avoid poor clinical outcomes, even if the quantities of nAbs ultimately achieved are not “too little.” This premise is suggested by passive immunization and convalescent plasma COVID-19 treatment trials in which early, but not later, treatment with nAbs is associated with improved virologic and clinical outcomes [19,20,76–79] and aligns with our observations regarding kinetics of the autologous nAb response.

Among all participants with samples at both V1 and V2, we observed a 3.5 (2.8)-fold decline in ID50 (ID80) GMT over approximately 2 months. Other studies have reported declines of 2- to 5-fold in the first 6 months post-diagnosis [34,48,50,53], but there has been limited comparison of the timing, magnitude, or rate of decline across demographic or clinical subgroups, where our data showed considerable variability. Among those with and without diabetes or hypertension and among younger compared to older individuals, nAb titers peaked around the same time but achieved significantly different magnitudes—higher among older adults and those with diabetes, lower among those with hypertension. We observed comparatively delayed peak nAb titers in hospitalized versus asymptomatic individuals or symptomatic outpatients, as reported elsewhere [49], and in those assigned male versus female sex at birth. However, our trial limited enrollment to those recovered from SARS-CoV-2 infection, and we therefore did not directly observe early innate and adaptive responses that may drive this.

The durability of SARS-CoV-2–induced nAb responses is unknown. In addition to later and higher peak nAb responses, we also observed more rapid nAb waning over time in several subgroups associated with worse clinical outcomes: Among asymptomatic, symptomatic outpatients, and individuals who were hospitalized, the ID80 GMT declined 2.2-, 2.3-, and 4-fold, respectively, and among those assigned female and male sex at birth, ID80 GMT declined 2.1- and 3.6-fold, respectively, in unadjusted analyses (**Table I in S1 Data**). The latter observation is also consistent with previously reported findings that males exhibit faster initial nAb decay [44,51], though these observations also likely reflect peak titers and the time of cohort sampling relative to diagnosis. Older adults maintained significantly higher nAb titers compared to their younger counterparts through 4 months post-diagnosis, with titers in both age groups declining about 3.5-fold. In contrast, titers among patients with diabetes declined at least 1.5 times more quickly than among patients without diabetes, and while hypertensive participants had lower nAb titers than those without hypertension at approximately 2 months post-diagnosis, hypertensive individuals’ lower titers declined significantly more slowly than those of their nonhypertensive counterparts, erasing much of the difference in titers between these groups by 4 months post-diagnosis.

The differences we observed in nAb kinetics among subgroups may reflect diverse immune mechanisms. For example, while peak nAb titers are higher and may occur later in some subgroups, T-cell help and SARS-CoV-2–specific memory B-cell proliferation may vary and ultimately support a more durable protective nAb response in some subgroups more than others [54–56]. An understanding of nAb kinetics and their underlying mechanisms is important to optimally inform infection monitoring and the development and implementation of vaccines, including potential boosters, and other preventative strategies.

As with the varying nAb kinetics we observed, it is likely that any nAb or broader immune response will differ depending on demographic and clinical cohort characteristics. At an average of less than 8 weeks post-diagnosis, 15% of our cohort had no detectable nAb titers (ID50 <20) and 24% had no detectable ID80 nAb titers. Nonresponders were more likely to be younger, report a milder infection, and have hypertension. Among the 186 participants evaluated at a second visit about 2 months after enrollment, 22% (45%) had no detectable ID50 (ID80) nAb titers, rates that correspond with published values of 20% to 33% of individuals with undetectable nAb titers within the first 6 months of recovering from infection [34,35,37,38,41,43].

The temporal nAb patterns we observed with SARS-CoV-2 contrast with observations from SARS-CoV-1 and severe MERS-CoV, in which nAb responses were detectable for years [80–85]. Instead, the kinetics we observed may be more akin to those of endemic, seasonal human coronaviruses (e.g., 229E, OC43, HKU1, and NL63), for which rapid decline in antibody responses and reinfection within 6 months to 1 year has been observed [82,86,87].

The nAb titers necessary for protection from SARS-CoV-2 infection or reinfection are unknown, and factors impacting nAbs over time remain unclear. One limitation of our study is its cross-sectional nature and initial sampling time point at 2 months post-SARS-CoV-2 diagnosis. The viral load and innate and adaptive responses during the acute period of infection and illness, which we did not observe, likely drive some of our observations and may differ across subgroups. In fact, recent data suggest that not only are nAb responses a correlate of protection from COVID-19, but also that binding antibody responses and nonneutralizing Fc effector functions play important roles as correlates and/or surrogates of protection (e.g., [18]). Our study may also be limited by survivorship bias, as we only sampled participants upon recovery from infection or illness, a bias more likely to impact subgroups with more severe illness. However, many of our observations are supported by smaller cohorts of acutely ill individuals. Furthermore, data from this large, diverse global cohort of post-convalescent individuals are highly relevant for understanding reinfection risk and for vaccine development and deployment.

Further exploration of the virologic and immunologic dynamics of vulnerable subgroups and nonresponders described here will aid in understanding the underlying protective mechanisms of durable anti-SARS-CoV-2 immune responses. This will inform next-generation vaccine development and monitoring policies, reinfection prevention, and vaccine boost strategies. To better understand the implications of SARS-CoV-2 nAb patterns on the clinical course of acute infection and protection from reinfection, exploration of nAb responses in the context of the broader immune responses over time and among demographic and clinical subgroups is likely to be particularly useful.

## Supporting information

S1 File STROBE Checklist. (DOCX)

S1 Text Supplemental list of clinical research sites and methods. (DOCX)

S2 Text Study protocol, statistical analysis plan. (PDF)

S1 Data Supporting information tables A-L. **Table A. Univariable association of COVID-19 severity, demographics, and medical history with nAb responses in Peru and the US. Table B. Associations of COVID-19 severity (asymptomatic, symptomatic, hospitalized), medical history, and demographics with nAb responses in Peru and the US. Table C. nAb GMT and 95% CI at enrollment visit by participant characteristics (including demographics, medical history, disease severity, and days since SARS-CoV-2 diagnosis). Table D. nAb response rate and 95% CI at enrollment visit by participants’ characteristics (including demographics, medical and smoking history, disease severity, and days since SARS-CoV-2 diagnosis). Table E. Associations of COVID-19 severity by age and sex assigned at birth with nAb ID50/ID80 titer at enrollment after adjusting for participants’ medical history, race/ethnicity, BMI, region, and days since SARS-CoV-2 diagnosis. Table F. Associations of COVID-19 severity, medical history, demographics, and days since SARS-CoV-2 diagnosis at enrollment (V1) with nAb ID50/ID80 titer fold-decline from V1 to V2 among participants with data available at both time points (V1 and V2). Table G. Associations of COVID-19 severity (asymptomatic, symptomatic, hospitalized no O**_**2**_**, hospitalized O**_**2**_**, hospitalized intubation/ICU), medical history, and demographics with nAb responses overall and by region (Peru and the US). Table H. Association of corticosteroid use on nAb responses in symptomatic outpatients and hospitalized individuals after adjusting for age, sex assigned at birth, BMI, diabetes, hypertension, and days since SARS-CoV-2 diagnosis. Table I. nAb GMT and 95% CI at the enrollment visit (V1) and 2-month post-enrollment visit (V2) and GMT ratio (V/V2) among participants with data available at both time points (V1 and V2). Table J. Association of ARBs and/or ACE inhibitor use (*n* = 49 use and *n* = 26 no use) and nAb responses, adjusting for COVID-19 severity, age, sex assigned at birth, diabetes, and days since SARS-CoV-2 diagnosis among participants with hypertension. Table K. Participant characteristics by responder or nonresponder and nAb ID50 or ID80 titer at enrollment. Table L. Associations of COVID-19 severity, medical history, demographics, and days since SARS-CoV-2 diagnosis with nAb nonresponse at enrollment.** ACE, angiotensin-converting enzyme; ARB, angiotensin-receptor blocker; BMI, body mass index; CI, confidence interval; COVID-19, Coronavirus Disease 2019; GMT, geometric mean titer; ICU, intensive care unit; nAb, neutralizing antibody; SARS-CoV-2, Severe Acute Respiratory Syndrome Coronavirus 2.
(DOCX)

S2 Data **Supporting information figures A-G. Fig A**. **GMT ID80 at enrollment visit and 95% CI by enrollment group, severity, medical history, and days since COVID-19 onset in Americas. Figure B. Estimated age effect on nAb log-titers stratifying by COVID-19 severity adjusting for COVID-19 severity, medical history, other demographics, and days since SARS-CoV-2 diagnosis using GAM. Fig C. GMT of nAb ID50 (A) and ID80 (B) titer at the enrollment visit in Americas.** White asterisks denote groups with fewer than 10 participants. **Fig D. Estimated days since SARS-CoV-2 diagnosis (days) effect on nAb log-titers stratifying by COVID-19 severity adjusting for COVID-19 severity, medical history, and demographics using GAM. Fig E. Estimated BMI effect on nAb log-titers stratifying by COVID-19 severity adjusting for COVID-19 severity, medical history, other demographics, and days since SARS-CoV-2 diagnosis using GAM. Fig F. GMT at enrollment visit and 95% CI by enrollment group, severity, medical history, and days since SARS-CoV-2 diagnosis in the US and Peru. A. Peru, ID50. B. Peru, ID80. C. US, ID50. D. US, ID80. Fig G. nAb titers by prolonged viral shedding status and COVID-19 severity.** BMI, body mass index; CI, confidence interval; COVID-19, Coronavirus Disease 2019; GAM, generalized additive model; GMT, geometric mean titer; nAb, neutralizing antibody; SARS-CoV-2, Severe Acute Respiratory Syndrome Coronavirus 2.
(PDF)

## Abbreviations

ACE2: angiotensin-converting enzyme 2
ARB: angiotensin-receptor blocker
BMI: body mass index
CI: confidence interval
COVID-19: Coronavirus Disease 2019
CoVPN: COVID-19 Prevention Network
CRS: clinical research site
GAM: generalized additive model
GAMM: generalized additive mixed model
GMR: geometric mean ratio
GMT: geometric mean titer
IRB: Institutional Review Board
LOD: limit of detection
Luc: luciferase
mAb: monoclonal antibody
nAb: neutralizing antibody
NTD: N-terminal domain
OR: odds ratio
RBD: receptor binding domain
RLU: relative Luc unit
SARS-CoV-2: Severe Acute Respiratory Syndrome Coronavirus 2
yo: years old
