# Supplementary material for: Neutralizing antibody responses over time in demographically and clinically diverse individuals recovered from SARS-CoV-2 infection in the United States and Peru: A cohort study
Source: PLoS Med. 2021 Dec 6;18(12):e1003868. doi: 10.1371/journal.pmed.1003868 (PMC8687542; doi:10.1371/journal.pmed.1003868)
Supplement: S1 Text — (DOCX) [file pmed.1003868.s002.docx]

**S1 Text. Supplemental clinical research sites and methods.**

**Clinical research site investigators and locations are listed below.**

Srilatha Edupuganti (Hope Clinic, Atlanta, GA); Valeria Cantos Lucio (Ponce de Leon, Atlanta, GA); Jason Farley (Johns Hopkins, Baltimore, MD); Paul A. Goepfert (Birmingham, AL); Lindsey R. Baden (Brigham and Womens Hospital, Boston, MA); Kenneth H. Mayer (Fenway Health, Boston, MA); Cynthia Gay (Chapel Hill, NC); Temitope Oyedele (AYAR at CORE, Chicago, IL); Juan Carlos Hinojosa Boyer (Asociacion Civil Selva Amazonica, Iquitos, Peru); Javier R. Lama (Barranco, Lima, Peru); Juan Jose Montenegro Idrogo (San Marcos/CITBM, Lima, Peru); Pedro Gonzales (San Miguel, Lima, Peru); Robinson Cabello (Via Libre, Lima, Peru); Raphael Landovitz (UCLA CARE Center, Los Angeles, CA); Spyros A. Kalams (Vanderbilt, Nashville, TN); Susan Abdalian (Adolescent Trials Unit, New Orleans, LA); Ellen Morrison (Bronx Prevention Center, New York, NY); Yael Hirsch-Moverman (Harlem Prevention Center, New York, NY); Hong Van Tieu (NY Blood Center, New York, NY); Magdalena Sobieszczyk (Physicians & Surgeons, New York, NY); Shobha Swaminathan (New Jersey Medical School, Newark, NJ); Ian Frank (Penn Prevention, Philadelphia, PA); Michael Keefer (University of Rochester, Rochester, NY); Susan P. Buchbinder (Bridge HIV, San Francisco, CA); M. Juliana McElrath (Seattle Vaccine Trials Unit, Seattle, WA); and Manya Magnus (George Washington, Washington, DC).

**Supplemental Methods**

Participants were enrolled at 26 clinical research sites (CRS) in Peru (4 CRSs in Lima; 1 CRS in Iquitos) and in the US (4 CRSs in New York; 2 CRSs each in Atlanta and Boston; 1 CRS each in Baltimore, Birmingham, Chapel Hill, Chicago, Los Angeles, Nashville, New Orleans, Newark, Philadelphia, Rochester, San Francisco, Seattle, and Washington, DC).

Eligibility criteria included persons age 18 or older; HIV negative; and for persons of childbearing age, a negative pregnancy test within 4 days of enrollment. Exclusion criteria included current COVID-19 disease; pregnant; prior receipt of SARS-CoV-2-specific antibodies or SARS-CoV-2 vaccination; or any other condition deemed by investigator to interfere with ability to comply with trial protocol or give informed consent. In total, 385 participants were enrolled in Peru or the US. Nineteen participants were not assayed for this analysis, due to samples not collected (n=4), or samples not available by the October 2020 cut-off date for assay and analysis completion for this work (n=15). The remaining cohort of 329 participants in this analysis of nAb responses also excludes individuals with HIV who had enrolled by the data cutoff (n=37) since antiretroviral therapy interferes with the validated pseudovirus assay we used to assess nAb activity.
