# Supplementary material for: Neutralizing antibody responses over time in demographically and clinically diverse individuals recovered from SARS-CoV-2 infection in the United States and Peru: A cohort study
Source: PLoS Med. 2021 Dec 6;18(12):e1003868. doi: 10.1371/journal.pmed.1003868 (PMC8687542; doi:10.1371/journal.pmed.1003868)
Supplement: S2 Text — (PDF) [file pmed.1003868.s003.pdf]

PROTOCOL

HVTN 405/HPTN 1901

Characterizing SARS-CoV-2-specific immunity in convalescent individuals

DAIDS DOCUMENT ID 38725

Non IND Study

CLINICAL TRIAL SPONSORED BY

Division of AIDS (DAIDS)
National Institute of Allergy and Infectious Diseases (NIAID)
National Institutes of Health (NIH)
Department of Health and Human Services (DHHS)
Bethesda, Maryland, USA

June 4, 2020
FINAL
HVTN 405/HPTN 1901
Version 2.0

FOR REVIEW ONLY

Contents

1 Overview....4
 1.1 Protocol Team....7
2 Ethical considerations....8
3 IRB/EC review considerations....9
 3.1 Minimized risks to participants....9
 3.2 Reasonable risk/benefit balance....9
 3.3 Equitable participant selection....10
 3.4 Appropriate informed consent....10
 3.5 Protect privacy/confidentiality....10
4 Background....12
 4.1 Rationale for trial concept....12
 4.2 Potential Risks and Benefits....14
5 Objectives and endpoints....16
 5.1 Primary objectives and endpoints....16
 5.2 Secondary objectives and endpoints....17
 5.3 Exploratory objectives....17
6 Statistical considerations....19
 6.1 Accrual and sample size calculations....19
 6.2 Statistical analyses....21
7 Selection and withdrawal of participants....24
 7.1 Inclusion criteria....24
 7.2 Exclusion criteria....24
 7.3 Participant termination from the study....25
8 Clinical procedures....26
 8.1 Informed consent....26
 8.2 Enrollment visit....27
 8.3 Follow-up visits....28
 8.4 Nasal and nasopharyngeal sampling....28
 8.5 Visit windows....28
9 Laboratory....29
 9.1 HVTN CRS laboratory procedures....29
 9.2 Total blood volume....29
 9.3 Endpoint assays....29
 9.4 Lab assay portfolio....31
 9.5 Exploratory studies....32
 9.6 Specimen storage and other use of specimens....32
 9.7 Biohazard containment....32
10 Protocol conduct....34
 10.1 Emergency communication with study participants....34
 10.2 Study termination....35
11 Version history....36

FOR REVIEW ONLY

12 Document references (other than literature citations)....38
13 Acronyms and abbreviations....40
14 Literature cited....42
Appendix A Sample informed consent form....45
Appendix B Table of procedures (for sample informed consent form)....57
Appendix C Schedule of procedures....58
Appendix D Visit windows....59
Appendix E Protocol Signature Page....60

FOR REVIEW ONLY

1 Overview

Title

Characterizing SARS-CoV-2-specific immunity in convalescent individuals

Primary objectives

Primary objective 1:

To identify serologic reactivities that differentiate SARS-CoV-2 infection from vaccination.

Primary objective 2:

To develop and formally qualify a suite of immunologic assays and reference reagents that permit detailed interrogations of the immune response to SARS-CoV-2 infection in preparation for similar assessments of vaccine-elicited immune responses and immunotherapeutics.

Primary objective 3:

To measure SARS-CoV-2-specific adaptive immune responses in order to identify immune markers of COVID-19 disease severity and duration in different demographic groups (eg, age, gender) and in people with different medical histories, including pre-existing conditions, new acute or chronic medical conditions, and concomitant medications.

Primary objective 4:

To characterize presentations of SARS-CoV-2 infection, including the clinical course of COVID-19, among convalescent individuals.

FOR REVIEW ONLY

Table 1-1 Cohorts

Group		N (approximate*)
**1**	**Persons not hospitalized for COVID-19, without clinical spectrum or outcomes specified in group 3**	
1A	Persons with asymptomatic infection, ages 18 through 55, inclusive	75-125
1B	Persons with asymptomatic infection, age > 55	50-75
1C	Persons with symptomatic infection (ie, COVID-19) ages 18 through 55	175
1D	Persons with symptomatic infection (ie, COVID-19), age > 55	75
**2**	**Persons previously hospitalized for COVID-19, without clinical spectrum or outcomes specified in group 3**	
2A	Persons 18 through 55 years of age	125
2B	Persons > 55 years of age	75
**3****	**Persons with specific clinical spectrums or outcomes, regardless of hospitalization history (eg, persons recovered after intubation, with prolonged viral shedding, with myocarditis/pericarditis, with rapid recovery from COVID-19, with a second positive SARS-CoV-2 RT-PCR test result after a negative result)**	100-150

*Actual sample size may be adjusted over time in response to evolving information regarding SARS-CoV-2 infection and COVID-19.

** See HVTN 405/HPTN 1901 Study Specific Procedures (SSP).

Participants

Approximately 675-800 volunteers aged 18 or older

Design

Observational cohort study

Duration per participant

Minimum one visit (1-8 weeks post resolution of COVID-19 OR 2-10 weeks post most recent positive SARS-CoV-2 test, if asymptomatic) and optional visits approximately 2 months, 4 months, and 1 year later. Additional follow up visit(s) may be added over time in response to evolving information regarding SARS-CoV-2 infection and COVID-19.

Estimated total study duration

15 months (includes enrollment) with possible extension in response to evolving information regarding SARS-CoV-2 infection and COVID-19.

Core operations

HVTN Vaccine Leadership Group/Core Operations Center, Fred Hutchinson Cancer Research Center (Fred Hutch) (Seattle, Washington, USA)

FOR REVIEW ONLY

Statistical and data management center (SDMC)

Statistical Center for HIV/AIDS Research and Prevention (SCHARP), Fred Hutch (Seattle, Washington, USA)

HVTN Laboratory Center (LC)

SARS-CoV-2 diagnostic laboratories

- University of Washington Virology Laboratory (Seattle, Washington, USA)
- Bio-Analytical Research Corporation – South Africa (BARC-SA) (Johannesburg, South Africa)

Endpoint assay laboratories

- Duke University Medical Center (Durham, North Carolina, USA)
- Fred Hutch/University of Washington (Seattle, Washington, USA)
- University of North Carolina Michael Hooker Research Center (Chapel Hill, North Carolina, USA)
- Cape Town HVTN Immunology Laboratory (Cape Town, South Africa)
- South Africa Immunology Laboratory and National Institute for Communicable Diseases (SAIL-NICD) (Johannesburg, South Africa)

Study sites

HIV Vaccine Trials Network (HVTN) and HIV Prevention Trials Network (HPTN) Clinical Research Sites (CRSs) in North and South America and in sub-Saharan Africa participating in this study to be specified in the Site Announcement Memo.

FOR REVIEW ONLY

1.1 Protocol Team

Protocol leadership

Chair	Larry Corey<br>HVTN Core, Fred Hutch<br>206-667-6770<br>lcorey@fredhtch.org	*Lead Statistician*	Sue Li<br>HVTN SDMC, Fred Hutch<br>206-667-7066<br>sli@fredhutch.org
Co-chairs	Shelly Karuna<br>HVTN Core, Fred Hutch<br>206-667-4355<br>mkaruna@fredhutch.org	*Medical officers*	Laura Polakowski<br>DAIDS, NIAID<br>240-627-3040<br>laura.polakowski@nih.gov
	Katherine Gill<br>Masiphumelele CRS<br>0829290991<br>katherine.gill@hiv-research.org.za		Julia Hutter<br>DAIDS, NIAID<br>240-627-3039<br>julia.hutter@nih.gov
Protocol Team leader	Shelly Karuna<br>HVTN Core, Fred Hutch<br>206-667-4355<br>mkaruna@fredhutch.org	*Laboratory leads*	John Hural<br>HVTN Laboratory Program<br>206-667-1683<br>jhural@fredhutch.org
			David Montefiori<br>HVTN Laboratory Program<br>919-684-5278<br>david.montefiori@duke.edu

Other contributors to the original protocol

Co-Protocol Team Leader	Will Hahn<br>HVTN Core, Fred Hutch	*Statisticians*	Ollivier Hyrien<br>Leigh Fisher<br>HVTN SDMC, Fred Hutch
Laboratory center representatives	Jen Hanke<br>Lisa Sanders<br>HVTN Laboratory Program, Fred Hutch	*Clinical trials manager*	Carissa Karg<br>HVTN Core, Fred Hutch
Regulatory affairs associate	Laurie Rinn<br>HVTN Core, Fred Hutch	*Clinical data managers*	Alison Ayres<br>Vicky Kim<br>HVTN SDMC, Fred Hutch
Clinic coordinator	Theresa Wagner<br>San Francisco Department of Public Health	*Clinical safety specialist*	Megan Jones<br>HVTN Core, Fred Hutch
Statistical research associates	Doug Grove<br>Shannon Grant<br>HVTN SDMC, Fred Hutch	*SDMC Associate director of lab science*	April Randhawa<br>HVTN SDMC, Fred Hutch
Community engagement unit representative	Gail Broder<br>HVTN Core, Fred Hutch	*Protocol development manager*	Meg Trahey<br>HVTN Core, Fred Hutch
Community educator representative	Ro Yoon<br>UW/Fred Hutch CRS	*Community Advisory Board member representative*	Nick Maurice, UW/Fred Hutch CRS CAB
Regional medical liaison Peru	Robert De La Grecca<br>HVTN Core, Fred Hutch	*Regional medical liaison sub-Saharan Africa*	Simba Takuva<br>HVTN Core, Fred Hutch

FOR REVIEW ONLY

2 Ethical considerations

It is critical that universally accepted ethical guidelines are followed at all sites involved in the conduct of clinical trials. The HIV Vaccine Trials Network (HVTN) and the HIV Prevention Trials Network (HPTN) have addressed ethical concerns in the following ways:

- HVTN and HPTN (Network) trials are designed and conducted to enhance the scientific knowledge base using methods that are scientifically rigorous and valid, and in accordance with International Council for Harmonisation of Technical Requirements for Pharmaceuticals for Human Use (ICH) and/or other Good Clinical Practice (GCP) guidelines.

- Network scientists and operational staff incorporate the philosophies underlying major codes (1-3), declarations, and other guidance documents relevant to human subjects research into the design and conduct of clinical trials.

- Network scientists and operational staff are committed to substantive community input—into the planning, conduct, and follow-up of its research—to help ensure that locally appropriate cultural and linguistic needs of study populations are met, in keeping with the Good Participatory Practices (GPP) for Emerging Pathogens guidance. Community Advisory Boards (CAB) are required by DAIDS and supported at all HVTN and HPTN research sites to ensure community input, in accordance with GPP and all local and national guidelines.

- The Networks provide training so that all participating sites similarly ensure fair participant selection, protect the privacy of research participants, and obtain meaningful informed consent. During the study, participants will have their wellbeing monitored, and to the fullest extent possible, their privacy protected. Participants may withdraw from the study at any time.

- Prior to implementation, Network trials are rigorously reviewed by scientists who are not involved in the conduct of the trials under consideration.

- Network trials are reviewed by local and national regulatory bodies and are conducted in compliance with all applicable national and local regulations.

- The Networks design their research to minimize risk and maximize benefit to both study participants and their local communities.

- The Networks value the role of in-country Institutional Review Boards (IRBs), Ethics Committees (ECs), and other Regulatory Entities (REs) as custodians responsible for ensuring the ethical conduct of research in each setting.

FOR REVIEW ONLY

3 IRB/EC review considerations

US federal regulations require IRBs/ECs/REs to ensure that certain requirements are satisfied on initial and continuing review of research (Title 45, Code of Federal Regulations (CFR), Part 46.111(a) 1-7; 21 CFR 56.111(a) 1-7). The following section highlights how this protocol addresses each of these research requirements. Each Network Investigator welcomes IRB/EC/RE questions or concerns regarding these research requirements.

This trial is being conducted in countries outside of the United States, with funding from the US NIH among others. Due to this, the trial is subject to both US and local regulations and guidelines on the protection of human research subjects and ethical research conduct. Where there is a conflict in regulations or guidelines, the Networks strive towards maximum protection of human research participants.

In compliance with international and local (as appropriate) ICH and/or other GCP guidelines, each research location has a locally-based Principal Investigator (PI) who is qualified to conduct (and supervise the conduct of) the research. The investigators take responsibility for the conduct of the study, including obtaining all appropriate regulatory and ethical reviews of the research.

3.1 Minimized risks to participants

45 CFR 46.111 (a) 1 and 21 CFR 56.111 (a) 1: Risks to subjects are minimized.

This protocol minimizes risks to participants by (a) correctly and promptly informing participants about risks so that they can join in partnership with the researcher in recognizing and reporting harms; (b) respecting local/national blood draw limits; and (c) having staff properly trained in administering study procedures that may cause physical harm or psychological distress, such as blood draws.

3.2 Reasonable risk/benefit balance

45 CFR 46.111(a) 2 and 21 CFR 56.111(a) 2: Risks to subjects are reasonable in relation to anticipated benefits, if any, to subjects, and the importance of the knowledge that may reasonably be expected to result.

In all public health research, the risk-benefit ratio may be difficult to assess because the benefits to a healthy participant are not as apparent as they would be in treatment protocols, where a study participant may be ill and may have exhausted all conventional treatment options. However, this protocol is designed

FOR REVIEW ONLY

to minimize the risks to participants while maximizing the potential value of the knowledge it is designed to generate.

3.3 Equitable participant selection

45 CFR 46.111 (a) 3 and 21 CFR 56.111 (a) 3: Subject selection is equitable

This protocol has specific inclusion and exclusion criteria for investigators to follow in admitting participants into the protocol. Participants are selected because of these criteria and not because of positions of vulnerability or privilege. Investigators are required to maintain screening and enrollment logs to document volunteers who screened into and out of the protocol and for what reasons.

3.4 Appropriate informed consent

45 CFR 46.111 (a) 4 and 5 and 21 CFR 56.111 (a) 4 and 5: Informed consent is sought from each prospective subject or the subject's legally authorized representative as required by 45 CFR 46.116 and 21 CFR Part 50; informed consent is appropriately documented as required by 45 CFR 46.117 and 21 CFR 50.27

The protocol specifies that informed consent must be obtained before any study procedures are initiated and assessed throughout the trial (see Section 8.1). Each site is provided training in informed consent by the Networks as part of its entering the Network. The HVTN and HPTN require a signed consent document for documentation, in addition to chart notes or a consent checklist.

3.5 Protect privacy/confidentiality

45 CFR 46.111 (a) 7 and 21 CFR 56.111 (a) 7: There are adequate provisions to protect the privacy of subjects and maintain the confidentiality of data.

Privacy refers to an individual's right to be free from unauthorized or unreasonable intrusion into his/her private life and the right to control access to individually identifiable information about him/her. The term "privacy" concerns research participants or potential research participants as individuals whereas the term "confidentiality" is used to refer to the treatment of information about those individuals. This protocol respects the privacy of participants by informing them about who will have access to their personal information and study data (see Appendix A). The privacy of participants is protected by assigning unique identifiers in place of the participant's name on study data and specimens. In the United States, research participants in HVTN and HPTN protocols are protected by a Certificate of Confidentiality from the US NIH, which can prevent disclosure

FOR REVIEW ONLY

of study participation even when that information is requested by subpoena. Participants are told of the use and limits of the certificate in the study consent form. In addition, each staff member at each study site in this protocol signs an Agreement on Confidentiality and Use of Data and Specimens with the HVTN. In some cases, a comparable confidentiality agreement process may be acceptable. Each study site participating in the protocol is required to have a standard operating procedure on how the staff members will protect the confidentiality of study participants.

FOR REVIEW ONLY

4 Background

4.1 Rationale for trial concept

The novel Severe Acute Respiratory Syndrome Coronavirus 2 (SARS-CoV-2) is one of three related betacoronaviruses that have caused highly pathogenic epidemics in humans; the others are Severe Acute Respiratory Syndrome Coronavirus (SARS-CoV-1) and Middle East Respiratory Syndrome Coronavirus (MERS-CoV). An initial outbreak of SARS-CoV-2, first reported in Wuhan, China, quickly became a pandemic and currently presents an unprecedented global public health challenge. Given the extent of dissemination, mathematical models suggest that SARS-CoV-2 will likely become endemic in human populations (4). Therefore, there is a critical need for an improved understanding of the immune response following natural infection in order to develop a vaccine to prevent transmission and to identify and develop strategies to distinguish vaccine-elicited responses from those induced by SARS-CoV-2 natural infection.

Early epidemiologic data suggest that specific populations are at higher risk for developing poor clinical outcomes during acute infection but even within defined populations there exist a variety of disease phenotypes (5). The variety of immune outcomes following natural infection is not well understood, and there is currently no preventive vaccine or effective therapy. The following study proposes to illuminate the variety of immune signatures after recovery from natural SARS-CoV-2 infection across a range of clinical phenotypes, to develop and refine immunologic assays and inform efforts to distinguish natural infection from vaccine-elicited responses in preparation for future preventive and therapeutic vaccine trials, and to explore whether clinical phenotypes are associated with particular immune outcomes.

The published data regarding the immune response following natural coronavirus infection are largely drawn from the experience with SARS-CoV-1 (6). SARS-CoV-1 and SARS-CoV-2 share ~79% sequence identity and use the same cellular receptor, however, there are several differences in spike contact sites (7). Neutralizing Ab responses to SARS-CoV-1 infection show considerable variability, but in general are first observed in the second week after onset of fever, peak during week 4, and persist in some cases for >200 days, but often wane considerably by 2 years post-infection (8-10). In convalescent sera 12 months post-infection with SARS-CoV-1, both IgG directed against whole cell lysates and neutralizing antibodies were detected in greater than 90% of infected patients. However, no correlation was observed between total IgG titers and degree of neutralization (11). Data following mild SARS-CoV-1 infection further suggest that these responses are likely to persist beyond one year, as CD4+ T cells capable of producing IFN-gamma in response to a variety of viral antigens were detected 12 years following natural infection (12, 13). This is in contrast to the duration of the humoral response following severe SARS-CoV-1 infection, as neither SARS-specific IgG nor SARS-specific memory B cells (as assessed via

FOR REVIEW ONLY

ELISpot) were detected in the periphery of patients who recovered from severe SARS (14).

Rapid and high neutralizing Ab responses that decline early are associated with greater disease severity and a higher risk of death from SARS-CoV-1 infection (9, 15, 16). A similar phenomenon was observed in a nonhuman primate model of SARS-CoV-1 infection, where ensuing acute lung injury was attributed to inflammatory cytokines and skewed macrophage functions (16). Additional variations in the immune response to coronavirus infection are associated with clinical disease. More PBMCs capable of producing IFN-gamma and or TNF-alpha were isolated 6 to 24 months after infection in patients recovered from severe MERS-CoV than in asymptomatic seroconverters (17). CD4+ T cells isolated from patients convalescing from severe SARS-CoV-1 infection were capable of recognizing more epitopes than those in patients with mild disease (11). Titers of antibodies directed against the MERS spike protein were also higher in patients with severe MERS disease than in those with milder disease (18), and higher titers of antibodies directed against the SARS-CoV-2 spike protein were recently reported for those with severe SARS-CoV-2 disease compared to those with milder disease (19).

It remains to be seen to what extent lessons learned from SARS-CoV-1 are helpful in formulating hypotheses about SARS-CoV-2. Recent neutralizing and non-neutralizing monoclonal antibodies (mAbs) from SARS-CoV-2 infected individuals provide some of the earliest insights (20). Non-neutralizing but not neutralizing mAbs bound SARS-CoV-1 and MERS-CoV Spike proteins. The neutralizing mAbs targeted the receptor binding domain (RBD), arose from multiple families of heavy and light chains, and possessed few somatic mutations, suggesting a relatively low barrier to vaccine elicitation. Many of the non-neutralizing antibodies targeted regions outside the RBD, which probably explains their lack of neutralizing activity. There appears to be limited cross-neutralization of SARS-CoV-1 and SARS-CoV-2 (20-22), although one group identified a monoclonal antibody from a convalescent SARS-CoV-1 subject that exhibits substantial neutralizing activity against SARS-CoV-2 (23).

As SARS-CoV-2 becomes endemic, understanding the immunity generated by natural infection will be critical for both public health efforts and rational vaccine design. Prior investigations have revealed both protective and harmful roles for pre-existing immunity. Human challenge models with endemic coronavirus (229E) have suggested that pre-challenge serum neutralizing antibody is significantly associated with protection from clinical disease (24). In addition, low pre-challenge secretory IgA was associated with the development of disease and prolonged viral shedding (defined as greater than 5 days), suggesting that pre-existing immune memory can ameliorate the clinical course of coronavirus disease and reduce transmissibility. Less is known about the specific role of CD8+ T cells but vaccines targeting CD8+ T-cell epitopes are capable of inducing protective immunity against homologous rechallenge in murine models (25, 26). Additional studies in preclinical models have demonstrated that there are cross-reactive CD4+ T cells between SARS-CoV-1 and MERS-CoV and that these T-

FOR REVIEW ONLY

cells are capable of mediating protection when they are located in the correct anatomic compartments (eg, in lung-resident CD4+ T cells) (19).

A critical unknown is the quality and duration of the adaptive immune response to SARS-CoV-2 infection and whether the immune response is associated with the underlying disease phenotype. Understanding the dynamics and duration, as well as the epitope specificity and other defining signatures, of the natural immune response can inform rational design and testing of preventive and therapeutic vaccines and monoclonal antibodies. In addition, the data could inform predictive models and public health decisions, such as decisions regarding population-specific return-to-work policies and surveillance practices during future epidemics.

To this end, data generated from this cohort study will facilitate the development and formal qualification of a suite of immunologic assays, including characterizing the kinetics, magnitude and duration of adaptive immune responses to SARS-CoV-2 infection; identifying and producing stable supplies of key reference agents; and ultimately establishing GCLP-compliant centralized laboratory activities that build upon and advance the Networks' existing laboratory expertise and resources. Furthermore, the study will generate highly standardized datasets of SARS-CoV-2-specific adaptive immunity; identify immune markers of COVID-19 disease severity and duration and shed light on the impact of demographic variables such as age and gender; and, perhaps most importantly, identify serologic reactivities that differentiate SARS-CoV-2 infection from vaccination, all of which will inform future vaccine and monoclonal antibody design and testing.

4.2 Potential Risks and Benefits

4.2.1 Potential Risks

The potential risks associated with this study are minimal and are primarily associated with sample collection and potential loss of confidentiality. The potential risks are outlined below.

Blood Draw by Venipuncture:

Blood drawing may cause pain and bruising and may, infrequently, cause a feeling of lightheadedness or fainting. Rarely, it may cause infection at the site where the blood is taken. Risk will be minimized by using sterile technique and universal precautions. Blood drawing may also cause anemia.

Urine sample collection:

There are no known risks for collection of urine by routine clean catch methods.

Nasopharyngeal or nasal swab, and nasal wash sample collection:

FOR REVIEW ONLY

Risks are minimal with a nasopharyngeal or nasal swab or a nasal wash. If a nasal or nasopharyngeal swab were misapplied, it could theoretically cause bleeding. Previous investigations using a similar washing strategy have been well tolerated (27). The major theoretical risk of instilling liquid into the nose (ie, a nasal wash) relate to the potential for aspiration into the respiratory tract. We will be using sterile solution, so we do not anticipate any risk of infection.

Maintenance of Confidentiality:

Participants will be asked to provide personal health information. Every effort will be made to protect participant's privacy and confidentiality (see Section 3.5).

4.2.2 Known potential benefits

There is no direct benefit for subjects who are enrolled in this study.

A better understanding of the clinical history of SARS-CoV-2 infection, virus persistence in different body fluids and the acquisition of samples to study infection and immune responses, including, in particular, responses that may distinguish natural infection from vaccination, constitutes a great potential benefit to society. These samples will assist in the understanding of the immune responses to SARS-CoV-2 infection and will help guide the development and testing of diagnostics, vaccines, monoclonal antibodies, and treatments for SARS-CoV-2 infection.

FOR REVIEW ONLY

5 Objectives and endpoints

5.1 Primary objectives and endpoints

Primary objective 1:

To identify serologic reactivities that differentiate SARS-CoV-2 infection from vaccination.

Primary endpoints 1:

Identification of humoral responses to peptide and protein antigens derived from SARS-CoV-2 structural proteins and regions of the spike protein not present in vaccines.

Primary objective 2:

To develop and formally qualify a suite of immunologic assays and reference reagents that permit detailed interrogations of the immune response to SARS-CoV-2 infection in preparation for similar assessments of vaccine-elicited immune responses and immunotherapeutics.

Primary endpoints 2:

- SARS-CoV-2-specific antibody responses including binding, neutralization, and Antibody-Dependent Cellular Cytotoxicity (ADCC)
- SARS-CoV-2-specific CD4+ and CD8+ T cell responses
- SARS-CoV-2-specific memory B cell characterization

Primary objective 3:

To measure SARS-CoV-2-specific adaptive immune responses in order to identify immune markers of COVID-19 disease severity and duration in different demographic groups (eg, age, gender) and in people with different medical histories, including pre-existing conditions, new acute or chronic medical conditions, and concomitant medications.

Primary endpoints 3:

- Response rate, magnitude, and epitope specificity of SARS-CoV-2-specific antibody neutralization activity, binding, and antibody-dependent cellular cytotoxicity (ADCC)
- Response rate, magnitude, and functional profiling of SARS-CoV-2-specific CD4+ and CD8+ T cells

FOR REVIEW ONLY

- Phenotypic characterization of SARS-CoV-2-specific memory B cells

Primary objective 4:

To characterize presentations of SARS-CoV-2 infection, including the clinical course of COVID-19, among convalescent individuals.

Primary endpoint 4:

Demographics and medical history, including pre-existing conditions, new acute or chronic medical conditions, and concomitant medications

5.2 Secondary objectives and endpoints

Secondary objective 1:

To determine if participants with a history of SARS-CoV-2 infection have detectable levels of virus

Secondary endpoint 1:

Detection of viral RNA in nasopharyngeal or nasal swab samples via RT-PCR

Secondary objective 2:

To determine if participants with a history of SARS-CoV-2 infection have detectable levels of SARS-CoV-2-specific antibodies in nasal cavity

Secondary endpoint 2:

Response rate and magnitude of SARS-CoV-2-specific binding antibodies in nasal samples

5.3 Exploratory objectives

Exploratory objective 1:

To evaluate associations of host genetics (eg, HLA, FcR, ACE-2, TMPRSS2 gene variants) with immune responses and clinical outcomes

Exploratory objective 2:

To investigate immune-mediated enhancement of infection and disease (eg, antibody-dependent enhancement of infection in vitro)

Exploratory objective 3:

FOR REVIEW ONLY

To assess cross-reactivity of immune responses with other pathogens (eg, other circulating coronaviruses)

Exploratory objective 4:

To further evaluate immune responses associated with SARS-CoV-2 infection, additional assays may be performed including, but not limited to, antibody avidity, antibody isolation and mAb production/functional characterization

Exploratory objective 5:

To conduct analyses related to furthering the understanding of SARS-CoV-2, immunology, vaccines, passive protection, and clinical trial conduct

FOR REVIEW ONLY

6 Statistical considerations

6.1 Accrual and sample size calculations

This protocol will enroll persons post report of recent SARS-CoV-2 infection (see Section 7). Recruitment will target enrolling 375-450 participants who were not hospitalized for COVID-19 (75-125 in Group 1A, 50-75 in Group 1B, 175 in Group 1C, and 75 in Group 1D), approximately 200 participants who were hospitalized (approximately 125 in Group 2A and 75 in Group 2B), and 100-150 participants who exhibited specific clinical spectrums or outcomes (Group 3, see cohort descriptions in Table 1-1).

6.1.1 Sample size calculations

One primary objective of this study is to characterize immune responses in participants with a history of SARS-CoV-2 infection and identify associations with the clinical course of COVID-19. The immune responses will be analyzed by measuring response rates and magnitudes among all participants. No adjustments for multiple comparisons will be made for the use of multiple assays. The precision with which the true response rate can be estimated from the observed data depends on the true underlying response rate and the sample size. Two-sided 95% confidence intervals for the response rate based on observing a particular rate of responses among all participants (n=675-800) or within a group (n=375-450, 200, 100-150) or within a subgroup (175, 125, 100, 75, 50) is shown in Table 6-1. Calculations are done using the score test method (28).

Table 6-1 Two-sided 95% confidence intervals for the true response rate based on observing a particular rate of responses among all participants (n = 675-800) and each group and subgroup (n=375-450, 200, 175, 150, 125, 100, 75, 50)

No. of responses	Observed response rate (%)	95% Confidence interval
400/800	50	[46.5, 53.5]
480/800	60	[56.6, 63.3]
560/800	70	[66.7, 73.1]
640/800	80	[77.1, 82.6]
720/800	90	[87.7, 91.9]
338/675	50.1	[46.3, 53.8]
405/675	60	[56.3, 63.6]
472/675	69.9	[66.4, 73.3]
540/675	80	[76.8, 82.8]
608/675	90.1	[87.6, 92.1]
225/450	50	[45.4, 54.6]
270/450	60	[55.4, 64.4]
315/450	70	[65.6, 74.1]
360/450	80	[76.1, 83.4]
405/450	90	[86.9, 92.4]

FOR REVIEW ONLY

No. of responses	Observed response rate (%)	95% Confidence interval
188/375	50.1	[45.1, 55.2]
225/375	60	[55, 64.8]
262/375	69.9	[65, 74.3]
300/375	80	[75.7, 83.7]
338/375	90.1	[86.7, 92.8]
188/375	50.1	[45.1, 55.2]
125/250	50	[43.8, 56.2]
150/250	60	[53.8, 65.9]
175/250	70	[64.1, 75.3]
200/250	80	[74.6, 84.5]
225/250	90	[85.7, 93.1]
100/200	50	[43.1, 56.9]
120/200	60	[53.1, 66.5]
140/200	70	[63.3, 75.9]
160/200	80	[73.9, 85]
180/200	90	[85.1, 93.4]
88/175	50.3	[43, 57.6]
105/175	60	[52.6, 67]
122/175	69.7	[62.5, 76]
140/175	80	[73.5, 85.3]
158/175	90.3	[85, 93.8]
75/150	50	[42.1, 57.9]
90/150	60	[52, 67.5]
105/150	70	[62.2, 76.8]
120/150	80	[72.9, 85.6]
135/150	90	[84.2, 93.8]
62/125	49.6	[41, 58.2]
75/125	60	[51.2, 68.2]
88/125	70.4	[61.9, 77.7]
100/125	80	[72.1, 86.1]
112/125	89.6	[83, 93.8]
50/100	50	[40.4, 59.6]
60/100	60	[50.2, 69.1]
70/100	70	[60.4, 78.1]
80/100	80	[71.1, 86.7]
90/100	90	[82.6, 94.5]
38/75	50.7	[39.6, 61.7]
45/75	60	[48.7, 70.3]
52/75	69.3	[58.2, 78.6]
60/75	80	[69.6, 87.5]
68/75	90.7	[82, 95.4]

FOR REVIEW ONLY

No. of responses	Observed response rate (%)	95% Confidence interval
25/50	50	[36.6, 63.4]
30/50	60	[46.2, 72.4]
35/50	70	[56.2, 80.9]
40/50	80	[67, 88.8]

The study is adequately powered to evaluate differences in response rates between groups. Combinations of true response rates for two groups that can be distinguished with statistical power of 80% and 90% are provided in Table 6-2. For example, we will have 80% (90%) power to detect the difference between Group 1 (n=375-450) and Group 2 (n=200) if the response rate is 63% (64-65%) in Group 1 and 50% in Group 2; we will have 80% (90%) power to detect the difference between Group 1 (n=375-450) and Group 3 (100-150) if the response rate is 64-67% (66-69%) in Group 1 and 50% in Group 3; and we will have 80% (90%) power to detect the difference between Group2 (n=200) and Group 3 (n=100-150) if the response rate is 66-68% (68-71%) in Group 2 and 50% in Group 3. These calculations use a Fisher's exact 2-sided test with a Type I error rate of 0.05.

Table 6-2 Power for comparison of response rates between 2 groups given different sample sizes in groups

True response rate Group 2 (%)	Minimum true response rate in Group 1 in order to detect a difference											
	80% power						90% power					
	n1=450 n2=200	n1=450 n2=150	n1=200 n2=150	n1=375 n2=200	n1=375 n2=100	n1=200 n2=100	n1=450 n2=200	n1=450 n2=150	n1=200 n2=150	n1=375 n2=200	n1=375 n2=100	n1=200 n2=100
10	19	20	22	19	22	24	21	22	24	21	25	26
20	31	32	34	32	35	37	33	34	37	33	37	39
30	42	44	46	43	46	48	44	46	48	45	49	51
40	53	54	56	53	57	58	55	56	58	55	59	61
50	63	64	66	63	67	68	64	66	68	65	69	71

6.2 Statistical analyses

All data from enrolled participants will be analyzed. Analyses for primary, secondary and exploratory endpoints will be performed using SAS and R statistical software. No formal multiple comparison adjustments will be employed for multiple primary immunogenicity endpoints, or secondary endpoints across assays. However, multiplicity adjustments will be made for certain immunogenicity assays when the assay endpoint is viewed as a collection of

FOR REVIEW ONLY

hypotheses (eg, testing multiple peptide pools to determine a positive response) or group comparisons.

6.2.1 Analysis variables

The analysis variables consist of participant characteristics such as demographics and medical history (including pre-existing conditions, new acute or chronic medical conditions, and concomitant medications), presentations of SARS-CoV-2 infection (including the clinical course of COVID-19), immunogenicity for primary, secondary, and exploratory objective analyses, and host genetics.

6.2.2 Primary and Secondary Analysis

Data from all enrolled participants will be evaluated and compared between groups (Group 1- Group 3) and between subgroups within Group 1- Group 2. Additional analyses may be performed, limited to participants with confirmed SARS-Cov-2 infection. All analyses will be performed for overall and by region (Americas and sub-Saharan Africa (SSA)). The comparison between regions (Americas vs SSA) may be conducted as well.

Discrete categorical assay endpoints (eg, response rates) will be analyzed by tabulating the frequency of positive response for each assay by antigen and group at each timepoint for which an assessment is performed. Crude response rates will be presented with their corresponding 95% confidence interval estimates calculated using the score test method (28). Fisher's exact tests will be used to compare the response rates of any 2 groups.

To compare the response rates among more than two groups, an overall test for any difference in crude response rate among the groups will be conducted, using a Chi-square test. When all pair-wise comparisons between the multiple groups are of interest, the Holm-Bonferroni procedure will be used to calculate adjusted p-values for multiplicity adjustment.

For quantitative assay data (eg, percentage of positive cells from the intracellular cytokine staining (ICS) assay), graphical and tabular summaries of the distributions by antigen and group at each time point will be made. Box plots and plots of estimated reverse cumulative distribution curves will be used for graphical display by antigen and group at each timepoint.

The difference between groups will be tested with a nonparametric Wilcoxon rank sum test if the data are not normally distributed and with a 2-sample t-test if the data appear to be normally distributed. To test for differences among more than two groups, first a Kruskal-Wallis test or an F-test (depending on the normality assumption) will be used to test for overall differences. Secondly, if the overall test is significant at the 2-sided 0.05 level, then pair-wide group comparisons will be done unless prespecified. When all pair-wise comparisons between the multiple groups are of interest, the Holm-Bonferroni procedure will be used to calculate adjusted p-values for multiplicity adjustment. An appropriate data

FOR REVIEW ONLY

transformation (eg, log10 transformation) may be applied to better satisfy assumptions of symmetry and homoscedasticity (constant variance).

In addition, we will perform the comparisons between groups adjusting for some relevant covariates (eg, gender, race, hospitalization status, underlying health conditions) using regression models.

Details for calculating a positive response, response magnitude, and additional analyses for each assay data will be provided in the statistical analysis plan.

Categorical variables of participants' characteristics and medical history etc. at the enrollment visit will be tabulated for overall and by group and compared between groups using chi-square test. The distribution of continuous variables of participants' characteristics and medical history etc. at the enrollment visit will be summarized and compared between groups using t-test.

6.2.3 Exploratory Analysis

Clinical outcomes and immune responses will be compared between host genetics (eg, HLA, FcR, ACE-2, and TMPRSS2 gene variants) groups. For a binary clinical outcome or immune response rate, we will use Fisher's Exact test and/or a logistic regression to assess the association of host genetic groups with clinical outcome and immune response rate after adjusting for participants' characteristics such as age, gender, race, hospitalization status, and underlying health conditions. For a continuous clinical outcome or immune response magnitude, we will use Wilcoxon rank sum or t-test or a general linear regression adjusting for other covariates to assess the association of host genetic group with clinical outcomes and immune response. Benjamini-Hochberg procedure (29) will be used to calculate q-values adjusting for multiple comparisons involving host genotype groups.

For assessing humoral and cellular responses such as pro-inflammatory cytokine, cross-reactivity of immune responses with other pathogens or evaluating immune responses measured from additional assays associated with SARS-CoV-2 infection, we will calculate the Spearman correlation between immune responses or use methods described in Section 6.2.2. Additional information about immunological outcome analysis methods will be detailed in the Statistical Analysis Plan.

FOR REVIEW ONLY

7 Selection and withdrawal of participants

Volunteers determined to be eligible, based on the inclusion and exclusion criteria, may be enrolled in the study. Final eligibility determination will depend on information available at the time of enrollment.

Investigators should always use good clinical judgment in considering a volunteer's overall fitness for trial participation. Some volunteers may not be appropriate for enrollment even if they meet all inclusion/exclusion criteria.

7.1 Inclusion criteria

1. Age 18 or older.
2. Reports having had a positive test for SARS-CoV-2.
3. Reports resolution of COVID-19 within 1-8 weeks of enrollment OR, if asymptomatic infection, reports positive SARS-CoV-2 test within 2-10 weeks of enrollment. Not excluded: individuals with symptoms consistent with residual sequelae of resolved COVID-19, in the clinical judgement of the investigator.
4. Access to a participating HVTN or HPTN CRS and willingness to be followed for the planned duration of the study.
5. Ability and willingness to provide informed consent.
6. Assessment of understanding: volunteer demonstrates understanding of this study.
7. Volunteers who were assigned female sex at birth: negative urine or serum beta human chorionic gonadotropin (β-HCG) pregnancy test within 4 days of enrollment visit (ie, prior to enrollment blood draw or nasal collections). Persons who are NOT of reproductive potential due to having undergone hysterectomy or bilateral oophorectomy (verified by medical records) or having reached menopause (no menses for ≥ 1 year), are not required to undergo pregnancy testing.

7.2 Exclusion criteria

1. Reports current COVID-19.
2. Pregnant.
3. Receipt of SARS-CoV-2 specific antibodies (eg, convalescent plasma or sera, monoclonal antibodies, hyperimmune globulin). Not excluded: antibody therapy without SARS-CoV-2 specificity (eg, IL-6 pathway inhibitors for COVID-19).

FOR REVIEW ONLY

4. SARS-CoV-2 vaccine(s) received in a prior vaccine trial.

5. Any medical, psychiatric, occupational, or other condition that, in the judgment of the investigator, would interfere with, or serve as a contraindication to, protocol adherence or a volunteer's ability to give informed consent.

7.3 Participant termination from the study

Under certain circumstances, an individual participant may be terminated from participation in this study. Specific events that will result in early termination include:

- Participant becomes pregnant,
- Participant refuses further participation,
- Participant relocates and remote follow-up or transfer to another HVTN or HPTN CRS is not possible,
- HVTN or HPTN CRS determines that the participant is lost to follow-up,
- Investigator decides, in consultation with Protocol Team leadership, to terminate participation (eg, if participant exhibits inappropriate behavior toward clinic staff), or
- Any condition where termination from the study is required by applicable regulations.

FOR REVIEW ONLY

8 Clinical procedures

The schedule of clinical and laboratory procedures is shown in Appendix C.

A study visit may be conducted remotely, such as via phone, text message, email, or other electronic means, in lieu of, or in combination with, in-person visits. Furthermore, some visit procedures may be conducted outside the CRS (eg, home nasal sample collection).

8.1 Informed consent

Informed consent is the process of working with participants so that they fully understand what will and may happen to them while participating in a research study. The protocol-specific informed consent form documents that a participant (1) has been informed about the potential risks, benefits, and alternatives to participation, and (2) is willing to participate in the study. Informed consent encompasses all written, verbal, and electronic study information HVTN and HPTN (Network) CRS staff provide to the participant, before and during the trial. Network CRS staff will obtain informed consent of participants according to HVTN MOP policies and procedures.

If any new information is learned that might affect the participants' decisions to stay in the trial, this information will be shared with trial participants. If necessary, participants will be asked to provide revised informed consent.

8.1.1 Protocol-specific consent forms

The protocol-specific consent form describes the study and all aspects of protocol participation, including study procedures. A sample protocol-specific consent form is located in Appendix A.

Each Network CRS is responsible for developing a protocol-specific consent form for local use, based on the sample protocol-specific consent form in Appendix A The consent form must be developed in accordance with requirements of the following:

- CRS's IRB/EC and any applicable REs,
- CRS's institution, and
- Elements of informed consent as described in Title 45, CFR Part 46 and Title 21 CFR, Part 50, and in ICH E6, Good Clinical Practice: Consolidated Guidance 4.8.

The sample informed consent form includes instructions for developing specific content.

FOR REVIEW ONLY

Regarding protocol registration, sites should follow procedures outlined in the current version of the DAIDS Protocol Registration Manual.

8.1.2 Assessment of Understanding

Study staff are responsible for ensuring that participants fully understand the study before enrolling them. This process involves reviewing the informed consent form with the participant, allowing time for the participant to reflect on the procedures and issues presented, and answering all questions completely.

An Assessment of Understanding is used to document the participant's understanding of key concepts in this study. Participants must verbalize understanding of all questions answered incorrectly. This process and the participant's understanding of the key concepts is to be recorded in source documentation at the site.

8.2 Enrollment visit

All inclusion and exclusion criteria must be assessed on the day of enrollment (ie, no separate screening visit) with the exception of a pregnancy test, which must be done within 4 days of enrollment visit. An individual who has signed the study-specific consent and meets all inclusion and exclusion criteria (see Sections 7.1 and 7.2) will be considered enrolled.

After the appropriate informed consent and assessment of understanding have been obtained the following procedures are performed:

- Urine or serum pregnancy test (for participants who were assigned female sex at birth). Persons who are NOT of reproductive potential due to having undergone hysterectomy or bilateral oophorectomy (verified by medical records) or having reached menopause (no menses for ≥ 1 year), are not required to undergo pregnancy testing.

- Medical history (including COVID-19 history), documented in the case history record.

- Obtaining of volunteer demographics in compliance with the NIH Policy on Reporting Race and Ethnicity Data: Subjects in Clinical Research, Aug. 8, 2001 (available at http://grants.nih.gov/grants/guide/notice-files/NOT-OD-01-053.html).

- Assessment of concomitant medications, including all treatment for COVID-19, prescription and nonprescription drugs, vitamins, topical products, alternative/complementary medicines (eg, herbal and health food supplements), recreational drugs, vaccinations, and allergy shots.

Sample collections are described in Appendix C.

FOR REVIEW ONLY

8.3 Follow-up visits

At all follow-up visits, which are optional, the following procedures are performed **before blood** sample collections:

- Urine or serum pregnancy test (for participants who were assigned female sex at birth). Persons who are NOT of reproductive potential due to having undergone hysterectomy or bilateral oophorectomy (verified by medical records) or having reached menopause (no menses for ≥ 1 year), are not required to undergo pregnancy testing.

- Medical history, updated from the enrollment visit and subsequent follow-up visits, to include new acute or chronic medical conditions.

- Assessment of concomitant medications, including prescription and nonprescription drugs, vitamins, topical products, alternative/complementary medicines (eg, herbal and health food supplements), recreational drugs, vaccinations, and allergy shots.

Sample collections are described in Appendix C.

8.4 Nasal and nasopharyngeal sampling

An optional nasal or nasopharyngeal swab may be collected at the enrollment visit and may also be collected at any follow-up visit at which a participant reports symptoms that per CRS clinician judgement are consistent with possible COVID-19. The swab may be collected by site staff or it may be self-collected (at home or in the clinic); both nostrils will be sampled.

An optional nasal wash (preferred collection method) or nasal swab (if nasal wash cannot be collected) may be collected at all visits. For nasal wash, a sterile solution (eg, normal saline) will be instilled into the nostril and then removed; this process will be repeated until sufficient sample is collected (about 5mL). The wash or swab may be collected by site staff or it may be self-collected at home or in the clinic.

8.5 Visit windows

Visit windows are included in Appendix D.

FOR REVIEW ONLY

9 Laboratory

9.1 HVTN CRS laboratory procedures

The HVTN 405/HPTN 1901 Site Processing Lab Instructions and SSP provide further guidelines for operational issues concerning the clinical and processing laboratories. These documents include special considerations for phlebotomy and guidelines for general specimen collection, specimen labeling and specimen processing.

Tube types for blood collection are specified in Appendix C. For tests performed locally, the local lab may assign appropriate tube types.

In specific situations, the blood collection tubes may be redirected to another laboratory or may require study-specific processing techniques. In these cases, laboratory special instructions will be posted on the protocol-specific section of the HVTN website.

Of note, all assays described below are performed as research assays to evaluate the ability of the SARS-CoV-2 virus to induce immune responses in the context of the participants' genetic background and are not approved for use in medical care. Results from these assays are not made available to participants or medical professionals to guide treatment decisions.

9.2 Total blood volume

Required blood volumes per visit are shown in Appendix C. Not shown is any additional blood volume that would be required if a serum pregnancy test needs to be performed. The additional blood volume would likely be minimal. The total blood volume drawn for each participant will not exceed 500 mL in any 56-day (8-week) period.

9.3 Endpoint assays

9.3.1 SARS-CoV-2 PCR

Real-time reverse transcription polymerase chain reaction (RT-PCR) assays to detect SARS-CoV-2 will be run on nasal and/or nasopharyngeal swab samples collected from study participants. After RNA extraction, specimens will be analyzed by a laboratory developed test using the World Health Organization's E/RdRp primer set, the N1/N2 primer set from the Centers for Disease Control and Prevention, or tests from Hologic (Panther Fusion), DiaSorin (Simplexa), or Roche (cobas). Novel methods to detect SARS-CoV-2 may also be used if applicable.

FOR REVIEW ONLY

9.3.2 Neutralizing antibody assay (NAb)

SARS-CoV-2–specific nAb assays may be performed on serum samples from study participants. The assay will test neutralization of S-pseudotyped and/or full-genome recombinant viruses as measured by a reduction in luciferase (Luc) reported gene expression after infection in ACE2-positive cells.

9.3.3 Binding antibody multiplex assay (BAMA)

SARS-CoV-2 specific total IgG binding antibodies may be assessed in serum samples and IgA binding antibodies may be measured in nasal samples. In addition, SARS-CoV-2–specific serum IgA and IgG subclass (IgG1, IgG2, IgG3, and IgG4) antibodies may also be assessed.

9.3.4 ACE-2 blocking assay

The ability of polyclonal sera to block binding of SARS-CoV-2 spike protein to the cellular ACE-2 receptor may be determined by biophysical assays utilizing Biolayer Interferometry (BLI) or Surface Plasmon Resonance (SPR) and/or cell-based flow cytometry assay.

9.3.5 Peptide microarray

Linear epitope specificities of purified serum IgG may be examined by peptide microarray using a SARS-CoV-2 peptide library, which contains 15-mer peptides that overlap by 12 amino acids.

9.3.6 Antibody avidity assay

Antibody avidity may be measured using BAMA with the addition of a dissociation step to calculate the antibody avidity index (BAMA-AI). BLI and/or SPR technologies may also be used to measure antibody avidity.

9.3.7 Antibody-dependent cellular cytotoxicity assay (ADCC)

ADCC activity may be assessed using serum samples from study participants. For the Granzyme B flow-based cytotoxicity assay, participant sera are incubated with effector cells sourced from human PBMCs and S protein-transfected 293F cells. ADCC is quantified as net percent granzyme B activity which is the percent of target cells positive for GranToxiLux (GTL), an indicator of granzyme B uptake, minus the percent of mock transfected target cells positive for GTL when incubated with effector cells and sera.

9.3.8 Antibody dependent enhancement (ADE) assays

SARS-CoV-2–specific ADE assays may be performed on serum samples from study participants. The assays will test enhancement of S-pseudotyped virus

FOR REVIEW ONLY

infection as measured by an increase in luciferase (Luc) reported gene expression after a single round of infection in ACE2+/FcR- and ACE2-/FcR+ cells.

9.3.9 T-cell responses by intracellular cytokine staining (ICS)

Flow cytometry may be used to examine SARS-CoV-2-specific CD4+ and CD8+ T-cell responses following stimulation of PBMCs with synthetic peptides that span the proteins encoded by the SARS-CoV-2 virus. Data will be reported as percentages of CD4+ or CD8+ T cells responding to a specific peptide pool. Additional cell surface markers, cytokines, or functional markers may also be analyzed. Additional assays may include evaluation of additional cytokines using a multiplexed secreted cytokine assay, mapping of individual peptide-level T- cell responses, and characterization of viral inhibition.

9.3.10 SARS-CoV-2 -specific B cell phenotyping and monoclonal antibody isolation

SARS-CoV-2 -specific memory B cells and plasmablasts induced by infection may be identified and characterized using fluorescently labeled recombinant proteins in combination with a flow cytometry phenotyping panel. In particular, SARS-CoV-2 spike and receptor binding domain-specific B cells and plasmablasts will be enumerated and may be further characterized for expression of memory, activation, inhibitory or other markers of interest. From a subset of individuals, SARS-CoV-2-specific memory B cells or plasmablasts will be isolated for B cell receptor sequencing and generation of recombinant monoclonal antibodies for in-depth characterization (e.g. function, binding-specificity, etc.).

9.3.11 Genotyping

Molecular human leukocyte antigen (HLA), Fc Receptors, ACE-2, and TMPRSS2 genotyping may be performed on enrolled participants using cryopreserved whole blood. Other genes, including those associated with immune responses (eg, immunoglobulin or T cell receptor genes) or SARS-CoV-2 infection may also be evaluated.

9.4 Lab assay portfolio

Additional assays may be performed per the HVTN Laboratory Center assay portfolio, which includes immune assessments such as those for cellular, humoral, and innate immune responses, and host genetics. The assay portfolio will be updated periodically to include new assays and adjust qualification levels of existing assays.

FOR REVIEW ONLY

9.5 Exploratory studies

Samples may be used for other testing and research related to furthering the understanding of SARS-CoV-2 immunology or vaccines. In addition, cryopreserved samples may be used to perform additional assays to support standardization and validation of existing or newly developed methods.

9.6 Specimen storage and other use of specimens

The HVTN stores specimens from all study participants indefinitely, unless a participant requests that specimens be destroyed or if required by IRB/EC, or RE.

Other use of specimens is defined as studies not covered by the protocol or the informed consent form for the main study (see Appendix A).

This research may relate to SARS-CoV-2, vaccines, passive protection, the immune system, and other diseases. This could include genetic testing and, potentially, genome-wide studies. This research is done only to the extent authorized in each study site's informed consent form, or as otherwise authorized under applicable law. Other research on specimens ("other use") will occur only after review and approval by the HVTN, the IRB/EC of the researcher requesting the specimens, and the IRBs/ECs/REs of the CRSs if required.

As part of consenting for the study, participants document their initial decision to allow or not allow their specimens to be used in other research, and they may change their decision at any time. The participant's initial decision about other use of their specimens, and any later change to that decision, is recorded by their CRS in a Web-based tool that documents their current decisions for other use of their specimens. The HVTN will only allow other research to be done on specimens from participants who allow such use.

CRSs must notify HVTN Regulatory Affairs if institutional or local governmental requirements pose a conflict with or impose restrictions on specimen storage or other use of specimens.

9.7 Biohazard containment

The transmission of blood-borne pathogens can occur through contact with contaminated needles, blood, and blood products, and the transmission of SARS-CoV-2 and other respiratory pathogens may occur through contact with contaminated respiratory droplets and aerosols. Appropriate precautions will be employed by all personnel in the collection, shipping and handling of all specimens for this study, as currently recommended by the CDC and the NIH or other applicable agencies.

FOR REVIEW ONLY

All dangerous goods materials, including Biological Substances, Category A or Category B, must be transported according to instructions detailed in the International Air Transport Association Dangerous Goods Regulations.

FOR REVIEW ONLY

10 Protocol conduct

This protocol and all actions and activities connected with it will be conducted in compliance with the principles of GCP (ICHe6), and according to DAIDS and HVTN policies and procedures as specified in the *HVTN Manual of Operations,* DAIDS Clinical Research Policies and Standard Procedures Documents including procedures for the following:

- Protocol registration, activation, and implementation;
- Informed consent and enrollment;
- Study participant reimbursement;
- Data collection, documentation, transfer, and storage;
- Participant confidentiality;
- Study follow-up and close-out;
- Quality control;
- Protocol monitoring and compliance;
- Specimen collection, processing, and analysis;
- Exploratory and ancillary studies and sub-studies; and
- Destruction of specimens.

Any policies or procedures that vary from DAIDS and HVTN standards or require additional instructions (eg, instructions specific to this study) will be described in the HVTN 405/HPTN 1901 *Study Specific Procedures.*

10.1 Emergency communication with study participants

As in all clinical research, this study may generate a need to reach participants quickly to avoid imminent harm, or to report study findings that may otherwise concern their health or welfare.

When such communication is needed, the CRS will request that its IRB/EC and any applicable RE expedite review of the message. If this review cannot be completed in a timeframe consistent with the urgency of the required communication, the site can contact the participant without IRB/EC approval if such communication is necessary to avoid imminent harm to the study participant. The CRS must notify the IRB/EC and any applicable RE of the matter as soon as possible.

FOR REVIEW ONLY

10.2 Study termination

This study may be terminated early by the determination of a pertinent national regulatory authority, NIH, or Office for Human Research Protections (OHRP). In addition, the conduct of this study at an individual HVTN or HPTN CRS may be terminated by the determination of the IRB/EC and any applicable RE.

FOR REVIEW ONLY

11 Version history

The Protocol Team may modify the original version of the protocol. Modifications are made to HVTN protocols via clarification memos, letters of amendment, or full protocol amendments.

The version history of, and modifications to, Protocol HVTN 405/HPTN 1901 are described below.

Protocol history and modifications

Date: June 4, 2020

Protocol version:2.0
Protocol modification: Full protocol amendment 1

Item 1	Added in Section 1, *Overview*, Section 2, *Ethical considerations*, Section 6, *Statistical considerations*, Section 13, *Acronyms and abbreviations*, and Appendix A, *Sample informed consent form*: Clinical research sites in sub-Saharan Africa
Item 2	Revised in Section 1, *Overview*, Section 6, Statistical considerations, and Appendix A, *Sample informed consent form*: sample size increased from 400 to a target of 800 with a range from 675-800
Item 3	Added to Section 7.1, *Inclusion criteria* #7, Section 8.2, *Enrollment visit* and Appendix C, *Schedule of procedures* as new footnote 9: pregnancy test may be completed within 4 days prior to enrollment.
Item 4	Added to Section 1, *Overview*: new diagnostic and endpoint laboratories
Item 5	Added to Appendix A, *Sample informed consent form*, and Appendix C, Schedule of procedures: optional antibody testing for SARS-CoV-2 and return of results to participants
Item 6	Revised in Section 8.1, *Informed consent*: requirements for signed form
Item 7	Revised in Appendix A, *Sample informed consent form*, item 13: medical emergency and study-related injury language
Item 8	Version history updated in Section 11
Item 9	Editorial changes: corrected minor formatting issues in Section 1, *Overview* and Appendix A, *Sample informed consent form* and updated date and version number on Title page
Item 10	Updated with changes described in Letter of Amendment 1 dated May 12, 2020

FOR REVIEW ONLY

Date: May 12, 2020

Protocol version: Version 1.0
Protocol modification: Letter of Amendment 1

Item 1 Added in Section 7.2, *Exclusion criteria*: Receipt of passive antibodies for SARS-CoV-2 infection (eg, convalescent plasma or sera, monoclonal antibody, hyperimmune globulin)

Item 2 Added in Section 7.2, *Exclusion criteria*: SARS-CoV-2 vaccine(s) received in a prior vaccine trial

Item 3 Clarified Section 8.2, *Enrollment visit*, and Section 8.3, *Follow-up visits*: pregnancy testing requirements

Item 4 Revised in Section 9.3.1, *SARS-CoV-2 PCR*: description for SARS-CoV-2 PCR test

Item 5 Revised in Section 9.3.2, *Neutralizing antibody assay (Nab)*: description of the neutralization assay

Item 6 Added in Section 1.1, *Protocol Team*: Regional medical liaison

Date: April 27, 2020

Protocol version:
Protocol modification:

Original protocol

FOR REVIEW ONLY

12 Document references (other than literature citations)

Other documents referred to in this protocol, and containing information relevant to the conduct of this study, include:

- Assessment of Understanding. Accessible through the HVTN protocol-specific website.
- Division of AIDS (DAIDS) Clinical Research Policies and Standard Procedures Documents. Available at https://www.niaid.nih.gov/research/daids-clinical-research-policies-standard-procedures
- Division of AIDS Protocol Registration Manual. Available at https://www.niaid.nih.gov/sites/default/files/prmanual.pdf
- HVTN Certificate of Confidentiality. Accessible through the HVTN website.
- HVTN 405/HPTN 1901 Special Instructions. Accessible through the HVTN protocol-specific website.
- HVTN 405/HPTN 1901 Study Specific Procedures. Accessible through the HVTN protocol-specific website.
- HVTN 405/HPTN 1901 Site Lab Instructions. Accessible through the HVTN protocol-specific website.
- HVTN Manual of Operations. Accessible through the HVTN website.
- Dangerous Goods Regulations (updated annually), International Air Transport Association. Available for purchase at https://www.iata.org/publications/dgr/Pages/index.aspx
- HVTN Lab Center assay portfolio.
- International Council on Harmonisation of Technical Requirements for Pharmaceuticals for Human Use (ICH) E6, Guideline for Good Clinical Practice: Section 4.8, Informed consent of trial subjects. Available at http://www.ich.org/products/guidelines/efficacy/article/efficacy-guidelines.html
- Good Participatory Practice Guidelines for Trials of Emerging (And Re-emerging) Pathogens that are Likely to Cause Severe Outbreaks in the Near Future and for Which Few or No Medical Countermeasures Exist WHO December 2016): https://www.avac.org/resource/good-participatory-practice-guidelines-trials-emerging-and-re-emerging-pathogens%C2%A0-are

FOR REVIEW ONLY

- Bill of Rights and Responsibilities. Accessible through the HVTN protocol-specific website.

- NIH Policy on Reporting Race and Ethnicity Data: Subjects in Clinical Research. Available at https://grants.nih.gov/grants/guide/notice-files/NOT-OD-01-053.html

- Requirements for Source Documentation in DAIDS Funded and/or Sponsored Clinical Trials. Available at https://www.niaid.nih.gov/sites/default/files/daids-sourcedocpolicy.pdf

- Title 21, Code of Federal Regulations, Part 50. Available at http://www.accessdata.fda.gov/scripts/cdrh/cfdocs/cfcfr/CFRSearch.cfm?CFRPart=50

- Title 45, Code of Federal Regulations, Part 46. Available at https://www.hhs.gov/ohrp/regulations-and-policy/regulations/45-cfr-46/index.html

See Section 14 for literature cited in the background and statistics sections of this protocol.

FOR REVIEW ONLY

13 Acronyms and abbreviations

Ab	antibody
ACE2	angiotensin-converting enzyme 2
ADCC	Antibody-dependent cellular cytotoxicity assay
AUC-MB	area-under-the-curve-magnitude-breadth
BAMA	Binding antibody multiplex assay
β-HCG	beta human chorionic gonadotropin
BLI	biolayer interferometry
CAB	Community Advisory Board
CDC	US Centers for Disease Control and Prevention
CFR	Code of Federal Regulations
COVID-19	Coronavirus Disease 2019
CRS	clinical research site
CTL	cytotoxic T lymphocyte
DAIDS	Division of AIDS (US NIH)
DHHS	US Department of Health and Human Services
EC	Ethics Committee
EIA	enzyme immunoassay
ELISA	enzyme-linked immunosorbent assay
ELISpot	enzyme-linked immunospot
FcR	Fc receptor
FDA	US Food and Drug Administration
Fred Hutch	Fred Hutchinson Cancer Research Center
GCP	Good Clinical Practice
GEE	generalized estimating equation
GPP-EP	Good Participatory Practices for Emerging Pathogens
GTL	GranToxiLux
HLA	human leukocyte antigen
HPTN	HIV Prevention Trials Network
HVTN	HIV Vaccine Trials Network
ICH	International Council on Harmonisation of Technical Requirements for Pharmaceuticals for Human Use
ICS	intracellular cytokine staining
IFN-γ	interferon gamma
IND	Investigational New Drug
IRB	Institutional Review Board
mAb	monoclonal antibody
MERS-CoV	Middle East Respiratory Syndrome Coronavirus
nAb	neutralizing antibody

FOR REVIEW ONLY

NIAID	National Institute of Allergy and Infectious Diseases (US NIH)
NIH	US National Institutes of Health
OHRP	US Office for Human Research Protections
PBMC	peripheral blood mononuclear cell
PCR	polymerase chain reaction
RE	regulatory entity
RT-PCR	reverse transcription polymerase chain reaction
SAHPRA	South African Healthcare Products Regulatory Authority
SAP	Statistical Analysis Plan
SARS-CoV	Severe Acute Respiratory Syndrome Coronavirus
SCHARP	Statistical Center for HIV/AIDS Research and Prevention
SDMC	statistical and data management center
SPR	surface plasmon resonance
SSP	study-specific procedures
TMPRSS2	Transmembrane protease serine 2
UW-VSL	University of Washington Virology Specialty Laboratory

FOR REVIEW ONLY

14 Literature cited

1. UNAIDS. Ethical considerations in biomedical HIV prevention trials. 2007 7/2007.

2. The National Commission for the Protection of Human Subjects of Biomedical and Behavioral Research. The Belmont Report: Ethical Principles and Guidelines for the Protection of Human Subjects of Research. 1979 4/18/1979.

3. Council for International Organizations of Medical Sciences (CIOMS). International ethical guidelines for biomedical research involving human subjects. Bull Med Ethics. 2002(182):17-23.

4. Yang CY, Wang J. A mathematical model for the novel coronavirus epidemic in Wuhan, China. Math Biosci Eng. 2020;17(3):2708-24.

5. Guan WJ, Ni ZY, Hu Y, Liang WH, Ou CQ, He JX, et al. Clinical Characteristics of Coronavirus Disease 2019 in China. N Engl J Med. 2020.

6. Liu WJ, Zhao M, Liu K, Xu K, Wong G, Tan W, et al. T-cell immunity of SARS-CoV: Implications for vaccine development against MERS-CoV. Antiviral Res. 2017;137:82-92.

7. Lu R, Zhao X, Li J, Niu P, Yang B, Wu H, et al. Genomic characterisation and epidemiology of 2019 novel coronavirus: implications for virus origins and receptor binding. Lancet. 2020;395(10224):565-74.

8. Temperton NJ, Chan PK, Simmons G, Zambon MC, Tedder RS, Takeuchi Y, et al. Longitudinally profiling neutralizing antibody response to SARS coronavirus with pseudotypes. Emerg Infect Dis. 2005;11(3):411-6.

9. Zhang L, Zhang F, Yu W, He T, Yu J, Yi CE, et al. Antibody responses against SARS coronavirus are correlated with disease outcome of infected individuals. J Med Virol. 2006;78(1):1-8.

10. Liu W, Fontanet A, Zhang PH, Zhan L, Xin ZT, Baril L, et al. Two-year prospective study of the humoral immune response of patients with severe acute respiratory syndrome. J Infect Dis. 2006;193(6):792-5.

11. Li CK, Wu H, Yan H, Ma S, Wang L, Zhang M, et al. T cell responses to whole SARS coronavirus in humans. J Immunol. 2008;181(8):5490-500.

12. Fan YY, Huang ZT, Li L, Wu MH, Yu T, Koup RA, et al. Characterization of SARS-CoV-specific memory T cells from recovered individuals 4 years after infection. Arch Virol. 2009;154(7):1093-9.

FOR REVIEW ONLY

13. Ng OW, Chia A, Tan AT, Jadi RS, Leong HN, Bertoletti A, et al. Memory T cell responses targeting the SARS coronavirus persist up to 11 years post-infection. Vaccine. 2016;34(17):2008-14.

14. Tang F, Quan Y, Xin ZT, Wrammert J, Ma MJ, Lv H, et al. Lack of peripheral memory B cell responses in recovered patients with severe acute respiratory syndrome: a six-year follow-up study. J Immunol. 2011;186(12):7264-8.

15. Ho MS, Chen WJ, Chen HY, Lin SF, Wang MC, Di J, et al. Neutralizing antibody response and SARS severity. Emerg Infect Dis. 2005;11(11):1730-7.

16. Liu L, Wei Q, Lin Q, Fang J, Wang H, Kwok H, et al. Anti-spike IgG causes severe acute lung injury by skewing macrophage responses during acute SARS-CoV infection. JCI Insight. 2019;4(4).

17. Zhao J, Alshukairi AN, Baharoon SA, Ahmed WA, Bokhari AA, Nehdi AM, et al. Recovery from the Middle East respiratory syndrome is associated with antibody and T-cell responses. Sci Immunol. 2017;2(14).

18. Choe PG, Perera R, Park WB, Song KH, Bang JH, Kim ES, et al. MERS-CoV Antibody Responses 1 Year after Symptom Onset, South Korea, 2015. Emerg Infect Dis. 2017;23(7):1079-84.

19. Zhao J, Yuan Q, Wang H, Liu W, Liao X, Su Y, et al. Antibody responses to SARS-CoV-2 in patients of novel coronavirus disease 2019. Clin Infect Dis. 2020.

20. Ju B, Zhang Q, Ge X, Wang R, Yu J, Shan S, et al. Potent human neutralizing antibodies elicited by SARS-CoV-2 infection. bioRxiv preprint doi: https://doiorg/101101/20200321990770. 2020.

21. Ou X, Liu Y, Lei X, Li P, Mi D, Ren L, et al. Characterization of spike glycoprotein of SARS-CoV-2 on virus entry and its immune cross-reactivity with SARS-CoV. Nat Commun. 2020;11(1):1620.

22. Wu F, Wang A, Liu M, Wang Q, Chen J, Xia S, et al. Neutralizing antibody responses to SARS-CoV-2 in a COVID-19 recovered patient cohort and their implications. medRxiv preprint doi: https://doiorg/101101/2020033020047365. 2020.

23. Pinto D, Park Y-J, Beltramello M, Walls A, Tortorici MA, Bianchi S, et al. Structural and functional analysis of a potent sarbecovirus neutralizing antibody. bioRxiv preprint doi: https://doiorg/101101/20200407023903. 2020.

FOR REVIEW ONLY

24. Callow KA. Effect of specific humoral immunity and some non-specific factors on resistance of volunteers to respiratory coronavirus infection. J Hyg (Lond). 1985;95(1):173-89.

25. Liu WJ, Lan J, Liu K, Deng Y, Yao Y, Wu S, et al. Protective T Cell Responses Featured by Concordant Recognition of Middle East Respiratory Syndrome Coronavirus-Derived CD8+ T Cell Epitopes and Host MHC. J Immunol. 2017;198(2):873-82.

26. Channappanavar R, Fett C, Zhao J, Meyerholz DK, Perlman S. Virus-specific memory CD8 T cells provide substantial protection from lethal severe acute respiratory syndrome coronavirus infection. J Virol. 2014;88(19):11034-44.

27. Hermelingmeier KE, Weber RK, Hellmich M, Heubach CP, Mosges R. Nasal irrigation as an adjunctive treatment in allergic rhinitis: a systematic review and meta-analysis. Am J Rhinol Allergy. 2012;26(5):e119-25.

28. Agresti A, Coull BA. Approximate is better than "exact" for interval estimation of binomial proportions. Am Stat. 1998;52(2):119-26.

29. Benjamini Y, Hochberg Y. Controlling the false discovery rate: a practical and powerful approach to multiple testing. Journal of the Royal Statistical Society, Series B. 1995;57(1):125-33.

FOR REVIEW ONLY

Appendix A Sample informed consent form

Title: Characterizing SARS-CoV-2-specific immunity in convalescent individuals
HVTN protocol number: HVTN 405/HPTN 1901
Site: [Insert site name]

Thank you for your interest in our research study. Please read this consent form or ask someone to read it to you. If you decide to join the study, we will ask you to sign or make your mark on this form. We will offer you a copy to keep. We will ask you questions to see if we have explained everything clearly. You can also ask us questions about the study.

Research is not the same as treatment or medical care. The purpose of a research study is to answer scientific questions.

Key information

- Being in this research study is voluntary. It is your choice.
- The purpose of this study is to learn more about infection with and recovery from the virus called SARS-CoV-2. Some people know this virus by the name "coronavirus." It can cause the disease called COVID-19. You are being asked to take part in this study because you are age 18 or older, had a positive test for SARS-CoV-2, and have recovered.
- Your participation in this study will last for at least 1 visit and may last up to about 1 year. Procedures will include blood draws and optional nasal sample collections at one required visit and 3 optional visits.
- The risks associated with this study are minimal and are primarily due to collecting certain samples and the potential loss of confidentiality.
- We do not expect the study to benefit you in any way.

About the study

The HIV Vaccine Trials Network (HVTN), HIV Prevention Trials Network (HPTN) and [Insert site name] are doing a study to learn more about SARS-CoV-2 infection and the COVID-19 disease that it can cause.

About 800 people will take part in this study at multiple sites across the United States, South America and sub-Saharan Africa. The researcher in charge of this study at this clinic is [Insert name of site PI]. The US National Institutes of Health (NIH) is paying for the study.

FOR REVIEW ONLY

1. Why are we doing this study?

The main purpose of this study is to learn more about SARS CoV-2 infection and how our bodies respond to and recover from it. This information can be used to help develop better tests for SARS-CoV-2 infection and COVID-19 disease and may help in developing future vaccines and treatments.

Joining the study

2. It is completely up to you whether or not to join the study.

Take your time in deciding. If it helps, talk to people you trust, such as your doctor, friends or family. If you decide not to join this study, or if you leave it after you have joined, your other care at this clinic and the benefits or rights you would normally have will not be affected. If you decide not to enroll today and want to come back at another time, we may need to repeat some procedures to make sure you are still eligible.

Being in the study

3. You will have at least 1 scheduled visit, and 3 more optional visits over about 1 year if you choose.

At the first visit, we will review your medical history to see if you meet the study requirements and want to join. If you are eligible, we will collect more information about your health and medications you take. We will also ask you about any symptoms you may have had related to COVID-19 disease. We may need to review your medical records for additional information. If we do, we will ask you to sign a medical release of information so we can get your records from your doctor.

At this visit, we will also:

- Take urine or blood to do a pregnancy test if you were assigned female sex at birth. You will not be able to join the study if you are pregnant. (People who are NOT capable of becoming pregnant due to total hysterectomy or bilateral oophorectomy (verified by medical records) or who have reached menopause (no menses for one year or more) are not required to have pregnancy testing.)

- Ask if you are willing to provide optional nasal swab and nasal wash samples. You may be able to do these procedures yourself at home or in the clinic, or the clinic staff can do them for you. This may depend on what the clinic is allowed to do, and may change over time.

- Take blood samples. We will ask if you want to know if you have antibodies for SARS-CoV-2. If you do, we will take an additional blood sample.

The first visit could last from 1 to 2 hours.

FOR REVIEW ONLY

When we take blood, the amount will depend on the lab tests we need to do. It will be some amount between about 62 mL and about 112.5 mL (or about one quarter to one half a cup). Your body will make new blood to replace the blood we take out.

Site: You may want to add a sentence to the end of the previous paragraph contextualizing the blood volumes described (eg, "To compare, people who donate blood in the US can give a total of about 500 mL in an 8-week period."). Modify the example for cultural relevance and alter blood volumes as necessary.

We would like to have you complete the optional visits so that we can check on your health, and so that we can collect samples to see how your immune responses develop or change over time. Some parts of these visits could be completed over the phone or through electronic communication such as text messages and email, in addition to the sample collections done in person. For the 3 optional follow-up visits scheduled at about 2 months, about 4 months, and about 1 year later, we will:

- Take urine or blood to do a pregnancy test if you were assigned female sex at birth. Your participation in the study will end if you are pregnant.

- Ask if you are willing to provide a nasal swab if you have any new signs or symptoms of possible COVID-19. You may be able to do this procedure yourself at home or in the clinic, or the clinic staff can do it.

- Ask if you are willing to provide a nasal wash sample. You may be able to do this procedure yourself at home or in the clinic, or the clinic staff can do it. If a nasal wash collection is not possible, we may ask you for a second nasal swab sample instead.

- Ask questions about your health and any medications you are taking.

- Take blood samples. If you haven't already provided a sample for antibody testing, we will ask if you want to know if you have antibodies for SARS-CoV-2. If you want to know, we will take an additional blood sample.

These visits could last from ½ hour to 2 hours.

Site: Insert Appendix B, Table of procedures (for informed consent form) in this section or distribute it as a separate sheet if it is helpful to your study participants. You are not required to do either.

Information about SARS-CoV-2 and COVID-19 is changing every day. We may ask you to complete additional visits that are not currently scheduled. This may be because we would like to obtain additional samples and/or information. We will keep you updated about any new information we learn that may change your decision to be involved in this study.

FOR REVIEW ONLY

We may contact you after the study ends (for example, to tell you about the study results).

4. **We will give you [Site: Insert compensation] for each study visit you complete.**

This amount is to cover the costs of [Site: Insert text]

US sites: Include the following paragraph. You can remove the box around the text.

Payments you receive for being in the study may be taxable. We may need to ask you for your Social Security number for tax reasons.

You do not have to pay anything to be in this study.

5. **The HVTN will use your samples for research related to your immune system's response to COVID-19.**

We will send your samples (without your name or other identifying information) to labs approved by the HVTN for this study, which are located in the United States and South Africa. In rare cases, some of your samples may be sent to labs approved by the HVTN in other countries for research related to this study.

Researchers may also do genetic testing related to this study on your samples. Your genes are passed to you from your birth parents. They affect how you look and how your body works. The differences in people's genes can help explain why some people get a disease while others do not. The genetic testing will only involve some of your genes, not all of your genes (your genome). The researchers will study only the genes related to the immune system and COVID-19, and those that affect how people get infected with the SARS-CoV-2 virus that causes COVID-19.

In some cases, researchers may take cells from your samples and grow more of them over time, so that they can continue to contribute to this study.

These tests done on your samples are for research purposes, not to check your health. If you choose to provide a sample for antibody testing for SARS-CoV-2, the clinic will provide you the results. For all other lab tests, we will not give the results to you or this clinic because those tests are not approved for use in making health care decisions. These labs are only approved to do research tests.

When your samples are no longer needed for this study, the HVTN will continue to store them.

FOR REVIEW ONLY

6. **When samples are no longer needed for this study, the HVTN wants to use them in other studies and share them with other researchers.**

The HVTN calls these samples "extra samples." The HVTN will only allow your extra samples to be used in other studies if you agree to this. You will mark your decision at the end of this form. If you have any questions, please ask.

Do I have to agree? No. You are free to say yes or no, or to change your mind after you sign this form. At your request, we will destroy all extra samples that we have. Your decision will not affect your being in this study or have any negative consequences here.

Where are the samples stored? Extra samples are stored in a secure central place called a repository. *[Site: choose one of the following two sentences. African sites should choose the sentence referencing the repository in South Africa. All other sites should choose the sentence referencing the repository in the United States.]* Your samples will be stored in the HVTN repository in South Africa. Your samples will be stored in the HVTN repository in the United States.

How long will the samples be stored? There is no limit on how long your extra samples will be stored. *[Site: Revise the previous sentence to insert limits if your regulatory authority imposes them.]*

Will I be paid for the use of my samples? No. Also, a researcher may make a new scientific discovery or product based on the use of your samples. If this happens, there is no plan to share any money with you. The researcher is not likely to ever know who you are.

Will I benefit from allowing my samples to be used in other studies? Probably not. Results from these other studies are not given to you, this clinic, or your doctor. They are not part of your medical record. The studies are only being done for research purposes.

Will the HVTN sell my samples and information? No, but the HVTN may share your samples with HVTN or other researchers. Once we share your samples and information, we may not be able to get them back.

How do other researchers get my samples and information? When a researcher wants to use your samples and information, their research plan must be approved by the HVTN. Also, the researcher's institutional review board (IRB) or ethics committee (EC) will review their plan. *[Site: If review by your institution's IRB/EC/RE is also required, insert a sentence stating this.]* IRBs/ECs protect the rights and well-being of people in research. If the research plan is approved, the HVTN will send your samples to the researcher's location.

What information is shared with HVTN or other researchers? The samples and information will be labeled with a code number. The key to the code will stay at this clinic. It will not be shared with anyone who does not need to know your

FOR REVIEW ONLY

name. Your name will not be part of the information. However, some information that we share may be personal, such as your race, ethnicity, and sex assigned at birth.

What kind of studies might be done with my extra samples and information? The studies will be related to COVID-19, vaccines, antibodies, the immune system and other diseases.

Researchers may also do genetic testing on your samples.

In some cases, researchers may take cells from your samples and grow more of them over time, so that they can continue to do research with them.

If you agree, your samples could also be used for genome-wide studies. In these studies, researchers will look at all of your genes (your genome). The researchers compare the genomes of many people, looking for common patterns of genes that could help them understand diseases. The researchers may put the information from the genome-wide studies into a protected database so that other researchers can access it, but your name and other personal information will not be included. Usually, no one would be able to look at your genome and link it to you as a person. However, if another database exists that also has information on your genome and your name, someone might be able to compare the databases and identify you. If others found out, it could lead to discrimination or other problems. The risk of this is very small. There may be other unknown risks.

Who will have access to my information in studies using my extra samples?

People who may see your information are:

- Researchers who use your extra samples and information for other research
- Government agencies that fund or monitor the research using your extra samples and information
- The researcher's Institutional Review Board or Ethics Committee
- Any regulatory agency that reviews clinical trials
- The people who work with the researcher

All of these people will do their best to protect your information. The results of any new studies that use your extra samples and information may be published. No publication will use your name or identify you personally.

7. We will do our best to protect your private information.

US sites: Check HIPAA authorization for conflicts with this section.

FOR REVIEW ONLY

Your study records and samples will be kept in a secure location. We will label all of your samples and most of your records with a code number, not your name or other personal information. However, it is possible to identify you, if necessary. We will not share your name with the lab that does the tests on your samples, or with anyone else who does not need to know your name.

Clinic staff will have access to your study records. Your records may also be reviewed by groups who watch over this study to see that we are protecting your rights, keeping you safe, and following the study plan. These groups include:

- The US National Institutes of Health and its study monitors,
- Any regulatory agency that reviews clinical trials,
- *Sites must include:* [Insert name of local IRB/EC] ,
- [Insert name of local and/or national regulatory authority as appropriate],
- The HVTN and people who work for them,
- The US Office for Human Research Protections.

All reviewers will take steps to keep your records private.

US sites: Include the following boxed text. You can remove the box around the text.

We have a Certificate of Confidentiality from the US government, to help protect your privacy. With the certificate, we do not have to release information about you to someone who is not connected to the study, such as the courts or police. Sometimes we can't use the certificate. Since the US government funds this research, we cannot withhold information from it. Also, you can still release information about yourself and your study participation to others.

The results of this study may be published. No publication will use your name or identify you personally.

We may share information from the study with other researchers. We will not share your name or information that can identify you.

Site: The text below may not be deleted or changed, per FDA requirement. You can remove the box.

A description of this clinical trial will be available on http://www.ClinicalTrials.gov. This website will not include information that can identify you. At most, the website will include a summary of the results. You can search this website at any time.

FOR PREVIEW ONLY

8. We may take you out of the study at any time.

We may take you out of the study if:

- you do not follow instructions,
- we think that staying in the study might harm you,
- you become pregnant, or
- the study is stopped for any reason.

Risks

9. The potential risks associated with this study are minimal.

This section describes the risks we know about. There may also be unknown risks. We will tell you if we learn anything new that may affect your willingness to stay in the study.

Risks of taking blood:

In this study, we will take blood. This procedure can cause bruising, pain, feeling faint or dizzy, soreness, redness, swelling, itching, a sore, or bleeding. Taking blood can cause a low blood cell count (anemia), making you feel tired.

Risks of the nasal swab procedure:

The feeling of having a small, soft-tipped swab inserted into your nostril and twirled around may be a little uncomfortable, but it should not be painful. There is a small chance there could be some bleeding, but this is unlikely.

Risks of the nasal wash procedure:

The nasal wash may be uncomfortable, but it should not be painful. There is a small chance that the sterile salt water could go into your throat, causing you to cough or feel like you are choking, but this is unlikely.

Risks of disclosure of your personal information:

We will take several steps to protect your personal information. Although the risk is very low, it is possible that your personal information could be given to someone who should not have it. If that happened, you could face discrimination, stress, and embarrassment. We can tell you more about how we will protect your personal information if you would like it.

Risks of genetic testing:

FOR REVIEW ONLY

It is unlikely, but the genetic tests done on your samples could show you may be at risk for certain diseases. If others found out, it could lead to discrimination or other problems. However, it is almost impossible for you or others to know your test results from the genetic testing. The results are not part of your study records and are not given to you.

US Sites, include the following paragraph. In the very unlikely event that your genetic information becomes linked to your name, a federal law called the Genetic Information Nondiscrimination Act (GINA) helps protect you. GINA keeps health insurance companies and employers from seeing results of genetic testing when deciding about giving you health insurance or offering you work. GINA does not help or protect you against discrimination by companies that sell life, disability or long-term care insurance.

Benefits

10. The study may not benefit you.

We do not expect the study to benefit you in any way. This study may help us learn more about SARS-CoV-2 infection and COVID-19 disease. The study results may be used to help others in the future.

Your rights and responsibilities

11. If you join the study, you have rights and responsibilities.

You have many rights that we will respect. You also have responsibilities. We list these in the Bill of Rights and Responsibilities for Research. We will give you a copy of it.

Leaving the study

12. Tell us if you decide to leave the study.

You are free to leave the study at any time and for any reason. Your care at this clinic and your legal rights will not be affected, but it is important for you to let us know.

Injuries

Sites: Approval from HVTN Regulatory Affairs (at vtn.core.reg@hvtn.org) is needed for any change to the section below (other than those that the instructions specifically request or those previously approved by HVTN Regulatory Affairs).

FOR REVIEW ONLY

13. **If you have a life-threatening emergency, call [insert local-appropriate emergency number (e.g. 911)] right away or seek help immediately.**

Contact us when the life-threatening medical emergency is over or as soon as you can. For all other medical problems or injuries related to this study, contact us immediately. We will treat you or refer you for treatment. *(Sites: insert locale-appropriate medical insurance language in the following sentence)* You and/or your health insurance will be responsible for treatment costs.

There are no funds to pay for a study-related injury, added treatment costs, loss of a job, or other costs to you or your family. Local or national law may give you rights to seek payment for some of these expenses. You always have the right to use the court system if you are not satisfied.

Questions

14. **If you have questions or problems at any time during your participation in this study, use the following important contacts.**

If you have questions about this study, contact
[name or title and telephone number of the investigator or other study staff].

If you have any symptoms that you think may be related to this study, contact
[name or title and telephone number of the investigator or other study staff].

This study has been reviewed and approved by a committee called the
[name of local IRB/EC]. If you have questions about your rights as a research participant, or problems or concerns about how you are being treated in this study, contact [name or title and telephone number of person on IRB/EC] , at the committee.

Paragraph for South African sites. The study has been structured in accordance with the Declaration of Helsinki (last updated October 2013) which deals with the recommendations guiding doctors in biomedical research involving human participants, the Ethics in Health Research: Principles, Structures and Processes Second Edition 2015, and Guidelines for Good Practice in the Conduct of Clinical Trials in Human Participants in South Africa. We can provide you with copies of these guidelines if you wish to review them.

If you want to leave this study, contact
[name or title and telephone number of the investigator or other study staff].

Remainder of section for South African sites only.

You can reach a study staff member 24 hours a day at [telephone number].

If you have questions about this trial you should first discuss them with your doctor or the ethics committee (contact details as provided on this form). After

FOR PREVIEW ONLY

you have consulted your doctor or the ethics committee and if they have not provided you with answers to your satisfaction, you should write to the South African Healthcare Products Regulatory Authority (SAHPRA) at:

The Chief Executive Officer
Dr Boitumelo Semete-Makokotlela
South African Health Products Regulatory Authority Private Bag X828
PRETORIA
0001
Tel: (012) 842 7629/26
e-mail: Boitumelo.Semete@sahpra.org.za

Your permissions and signature

15. In Section 6 of this form, we told you about possible other uses of your extra samples and information, outside this study. Please choose only one of the options below and write your initials or make your mark in the box next to it. Whatever you choose, the HVTN keeps track of your decision about how your samples and information can be used. You can change your mind after signing this form.

☐ I allow my extra samples and information to be used for other studies related to COVID-19, vaccines, antibodies, the immune system, and other diseases. This may include genetic testing and keeping my cells growing over time.

OR

☐ I agree to the option above *and* also to allow my extra samples and information to be used in genome wide studies.

OR

☐ I do not allow my extra samples to be used in any other studies. This includes not allowing genetic testing, growing more of my cells, or genome wide studies.

16. If you agree to join this study, you will need to sign or make your mark below. Before you sign or make your mark on this consent form, make sure of the following:

- You have read this consent form, or someone has read it to you.
- You feel that you understand what the study is about and what will happen to you if you join. You understand what the possible risks and benefits are.
- You have had your questions answered and know that you can ask more.

FOR REVIEW ONLY

- You agree to join this study.

You will not be giving up any of your rights by signing this consent form.

Participant's name (print)	Participant's signature or mark	Date	Time
Clinic staff conducting consent discussion (print)	Clinic staff signature	Date	Time

For participants who are unable to read or write, a witness should complete the signature block below:

Witness's name (print)	Witness's signature	Date	Time

*Witness is impartial and was present for the entire discussion of this consent form.

FOR REVIEW ONLY

Appendix B Table of procedures (for sample informed consent form)

Procedure	Visit 1<br>Month 0	Optional Visit 2<br>Month 2	Optional Visit 3<br>Month 4	Optional Visit 4<br>Month 12
Medical history	√	√	√	√
Pregnancy test (if appropriate*)	√	√	√	√
Blood drawn	√	√	√	√
Optional nasal swab	√	√**	√**	√**
Optional nasal wash sample	√***	√***	√***	√***
Interview/questionnaire	√	√	√	√

* For participants assigned female sex at birth. Pregnancy test may be performed on urine or blood samples. Persons who are NOT capable of becoming pregnant due to total hysterectomy or bilateral oophorectomy (verified by medical records) or who have reached menopause (no menses for one year or more) are not required to undergo pregnancy testing.

**Nasal swab may be collected at these visits if the participant reports new symptoms that are consistent with possible COVID-19.

*** If a nasal wash collection is not possible, participants may be asked to provide a second nasal swab sample instead.

FOR REVIEW ONLY

Appendix C Schedule of procedures

**Visit Number**		**01**	**02**[1]	**03**[1]	**04**[1]
**Study Month**		0	2	4	12
**Study Day**		0	56	112	364
**Study procedures**					
Assessment of Understanding		√	—	—	—
Informed consent		√	—	—	—
Eligibility confirmation		√	—	—	—
Demographics		√	—	—	—
Medical history		√	√	√	√
Concomitant medications[2]		√	√	√	√
**Clinical labs**	**Tube (size)**				
Pregnancy test (urine or serum)[3]		√[9]	√	√	√
Optional SARS-CoV-2 IgG assay[4]	SST (8.5mL)	8.5	—	—	—
**Research samples**[5]					
Cells for assays & storage[6]	ACD (8.5mL)	42.5–85	42.5–85	42.5–85	42.5–85
Serum for assays and storage	SST (8.5mL)	17	17	17	17
Whole blood for assays and storage	EDTA (2mL)	2	—	—	—
Optional nasal or nasopharyngeal swab		√	√[7]	√[7]	√[7]
Optional nasal wash or swab sample		√	√	√	√
Daily volume (mL)		61.5–112.5	59.5–102	59.5–102	59.5–102
56-day total volume (mL)[8]		112.5	214.5	204	102

[1] These visits are optional. Additional optional visits may be conducted based on evolving information regarding SARS-CoV-2 infection and COVID-19.

[2] Concomitant medications (see Section 8.2 and 8.3).

[3] Pregnancy test (see Section 7.1). Pregnant participants will be terminated from the study (see Section 7.3).

[4] Specimen for optional SARS-CoV-2 IgG assay may be collected at interim or other optional visits if not collected at first clinic visit. See Specimen Collection SSP.

[5] Research samples: Blood draw volumes for each tube type shown.

[6] A minimum of 42.5mL up to a maximum 85mL of ACD blood will be collected. Within this range, the volume of ACD blood to be drawn should be determined in consideration of the current and planned 56-day blood draw volume total. See HVTN 405/HPTN 1901 Study Specific Procedures.

[7] An optional nasal or nasopharyngeal swab may be collected at these visits if the participant reports onset of new symptoms that in the clinician's judgment are consistent with possible COVID-19 (see Section 8.4).

[8] 56-day total based on maximum daily collection volume.

[9] Pregnancy test may be completed within 4 days prior to enrollment.

FOR REVIEW ONLY

Appendix D Visit windows

Visit Number	Visit Type	Lower Allowable Window	Lower Target Day	Target Day	Upper Target Day	Upper Allowable Window
01.0	**Enrollment**	-	-	0	-	+0
02.0[2]	Follow-up	-	-28	Day 56	+28	-
03.0[1,2]	Follow-up	-	-27	Day 112	+56	-
04.0[2]	Follow-up	-84	-28	Day 364	+28	+84

[1]Visit 3 must occur at least 28 days after Visit 2.
[2]These visits are optional. Additional optional visits may be conducted based on evolving information regarding SARS-CoV-2 infection and COVID-19.

FOR REVIEW ONLY

Appendix E Protocol Signature Page

Characterizing SARS-CoV-2-specific immunity in convalescent individuals

I will conduct the study in accordance with the provisions of this protocol and all applicable protocol-related documents. I agree to conduct this study in compliance with United States (U.S.) Health and Human Service regulations (45 CFR 46); applicable U.S. Food and Drug Administration regulations; standards of the International Conference on Harmonization Guideline for Good Clinical Practice (E6); Institutional Review Board/Ethics Committee determinations; all applicable in-country, state, and local laws and regulations; and other applicable requirements (eg, U.S. National Institutes of Health, Division of AIDS) and institutional policies

Investigator of Record Name (print)	Investigator of Record Signature	Date

DAIDS Protocol Number: HVTN 405/HPTN 1901

DAIDS Protocol Version: Version 2.0

Protocol Date: June 4, 2020

FOR REVIEW ONLY

HVTN 405/HPTN 1901

Protocol Version 2.0

Characterizing SARS-CoV-2-specific immunity in convalescent individuals

August 24, 2021
SAP version 1.3

Prepared by: Shuying Sue Li, PhD, Ollivier Hyrien, PhD, Leigh Fisher, PhD, Doug Grove, MS, Shannon Grant, MS

Approval Signature Page

HVTN 405/HPTN 1901

Statistical Analysis Plan

Characterizing SARS-CoV-2-specific immunity in convalescent individuals

I have read this Statistical Analysis Plan and approve its contents.

________________________________ ________________________

Shuying Sue Li, PhD Date

Lead Protocol Statistician

HVTN, FHCRC

SAP Modification History

The version history of, and modifications to, this statistical analysis plan are described below.

Date: 26 Aug 2020
SAP version: Version 1.0

Date: 10 Sept 2020
SAP version: version 1.1
Modification: adding Section 8.3 CD4 and CD8 T Cell

Date: 08 Dec 2020
SAP version: version 1.2
Modification: adding Section 8.4 Analysis of BCP data

Date: 24 Aug 2021
SAP version: version 1.3
Modification: adding %S+ IgM+ of total B cells definition in Section 8.4 Analysis of BCP data

Table of Contents

1 OVERVIEW **6**

2 PROTOCOL SUMMARY **6**

COHORT **7**

3 OBJECTIVES AND ENDPOINTS **7**

3.1 Primary Objectives and Endpoints 7

3.2 Secondary Objectives and Endpoints 8

3.3 Exploratory Objectives and Endpoints 8

4 COHORT DEFINITION **9**

5 POTENTIAL CONFOUNDERS **9**

6 STATISTICAL ANALYSIS **9**

6.1 Analysis Variables 10

6.2 General Approach for Immunological Data 10

6.3 Primary and Secondary Analysis 11

6.3.1 Primary Objective 1 11

6.3.2 Primary Objective 2 12

6.3.3 Primary Objective 3 12

6.3.4 Primary Objective 4 13

6.3.5 Secondary Objectives 1 and 2 14

6.4 Exploratory Analysis 14

6.4.1 Exploratory Objective 1 14

6.4.2 Exploratory Objectives 2-5 14

6.5 Analyses Prior to End of Study 14

7 STUDY PARTICIPANT CHARACTERISTICS **14**

7.1 List of Tables 15

8 ASSAY-SPECIFIC TABLES AND FIGURES FOR PROTOCOL TEAM REPORTS **15**

8.1 Binding Antibody 15

8.1.1 List of Tables 16

8.1.2 List of Graphs 16

8.2 Neutralizing Antibody 16

8.2.1 List of Tables 17

8.2.2 List of Graphs 17

8.3 CD4 and CD8 T Cell 17

8.3.1 List of Tables (for IFN-γ and/or IL2 for CD4+ and CD8+ T cells) 19

8.3.2 List of Graphs (for IFN-γ and/or IL2 for CD4+ and CD8+ T cells) 19
8.3.3 Additional List of Tables 19

1 OVERVIEW

The following describes the Statistical Analysis Plan (SAP) for the analysis of data from HVTN 405/HPTN 1901 according to the protocol version 2. As detailed in SCHARP SOP-0013, Revision 5 (effective date: August 15, 2016), this SAP is required prior to the first analysis and must be approved by the lead protocol statistician. Any subsequent revisions are expected to describe analysis of immunogenicity data and are archived.

2 PROTOCOL SUMMARY

Title
Characterizing SARS-CoV-2-specific immunity in convalescent individuals

Primary objectives
Primary objective 1:
To identify serologic reactivities that differentiate SARS-CoV-2 infection from vaccination.

Primary objective 2:
To develop and formally qualify a suite of immunologic assays and reference reagents that permit detailed interrogations of the immune response to SARS-CoV-2 infection in preparation for similar assessments of vaccine-elicited immune responses and immunotherapeutics.

Primary objective 3:
To measure SARS-CoV-2-specific adaptive immune responses in order to identify immune markers of COVID-19 disease severity and duration in different demographic groups (e.g., age, sex assigned at birth) and in people with different medical histories, including pre-existing conditions, new acute or chronic medical conditions, and concomitant medications.

Primary objective 4:
To characterize presentations of SARS-CoV-2 infection, including the clinical course of COVID-19, among convalescent individuals.

Cohort

Group		N (approximate*)
**1**	**Persons not previously hospitalized for COVID-19 and without any of the clinical spectrums or outcomes specified in group 3**	
1A	Persons with SARS-CoV-2 infection, ages 18 through 55, inclusive	75-125
1B	Persons with asymptomatic/presymptomatic SARS-CoV-2 infection, age > 55	50-75
1C	Persons with symptomatic SARS-CoV-2 infection ages 18 through 55	175
1D	Persons with symptomatic SARS-CoV-2 infection, age > 55	75
**2**	**Persons previously hospitalized for COVID-19 but without any of the clinical spectrums or outcomes specified in group 3**	
2A	Persons 18 through 55 years of age	125
2B	Persons > 55 years of age	75
**3****	**Persons with specific clinical spectrums or outcomes, regardless of hospitalization history (e.g., persons recovered after intubation, with prolonged viral shedding, with myocarditis/pericarditis, with rapid recovery from COVID-19, with a second positive SARS-CoV-2 NAAT*** or antigen test result after a negative result)**	100-150

*Actual sample size may be adjusted over time in response to evolving information regarding SARS-CoV-2 infection and the COVID-19 pandemic.

** See HVTN 405/HPTN 1901 Study Specific Procedures (SSP).

*** NAAT = nucleic acid amplification test.

3 OBJECTIVES AND ENDPOINTS

3.1 Primary Objectives and Endpoints

Primary objective 1:
To identify serologic reactivities that differentiate SARS-CoV-2 infection from vaccination

Primary endpoints 1:
Identification of humoral responses to peptide and protein antigens derived from SARS-CoV-2 structural proteins and regions of the spike protein not present in vaccines

Primary objective 2:
To develop and formally qualify a suite of immunologic assays and reference reagents that permit detailed interrogations of the immune response to SARS-CoV-2 infection in preparation for similar assessments of vaccine-elicited immune responses and immunotherapeutics.

Primary endpoints 2:

- SARS-CoV-2-specific antibody responses including binding, neutralization, and antibody-dependent cellular cytotoxicity (ADCC)
- SARS-CoV-2-specific CD4+ and CD8+ T-cell responses
- SARS-CoV-2-specific memory B cell characterization

Primary objective 3:
To measure SARS-CoV-2-specific adaptive immune responses in order to identify immune markers of COVID-19 disease severity and duration in different demographic groups (e.g., age, sex assigned at birth) and in people with different medical histories, including pre-existing conditions, new acute or chronic medical conditions, and concomitant medications

Primary endpoints 3:

- Response rate, magnitude, and epitope specificity of SARS-CoV-2-specific antibody neutralization activity, binding, and ADCC
- Response rate, magnitude, and functional profiling of SARS-CoV-2-specific CD4+ and CD8+ T cells
- Phenotypic characterization of SARS-CoV-2-specific memory B cells

Primary objective 4:
To characterize presentations of SARS-CoV-2 infection, including the clinical course of COVID-19, among convalescent individuals

Primary endpoint 4:
Demographics and medical history, including pre-existing conditions, new acute or chronic medical conditions, and concomitant medications

3.2 Secondary Objectives and Endpoints

Secondary objective 1:
To determine if participants with a history of SARS-CoV-2 infection have detectable levels of virus

Secondary endpoint 1:
Detection of viral RNA in nasopharyngeal and/or nasal swab samples via NAAT

Secondary objective 2:
To determine if participants with a history of SARS-CoV-2 infection have detectable levels of SARS-CoV-2-specific antibodies in the nasal cavity

Secondary endpoint 2:
Response rate and magnitude of SARS-CoV-2-specific binding antibodies in nasal samples

3.3 Exploratory Objectives and Endpoints

Exploratory objective 1:
To evaluate associations of host genetics (e.g., HLA, FcR, ACE-2, TMPRSS2 gene variants) with immune responses and clinical outcomes

Exploratory objective 2:
To investigate immune-mediated enhancement of infection and disease (e.g., antibody-dependent enhancement of infection in vitro)

Exploratory objective 3:
To assess cross-reactivity of immune responses with other pathogens (e.g., other circulating coronaviruses)

Exploratory objective 4:

To further evaluate immune responses associated with SARS-CoV-2 infection, additional assays may be performed including, but not limited to, antibody avidity, antibody isolation, and mAb production/functional characterization

Exploratory objective 5:
To conduct analyses related to furthering the understanding of SARS-CoV-2, immunology, vaccines, passive protection, and clinical trial conduct

4 COHORT DEFINITION

Participants
Approximately 675-800 volunteers aged 18 or older in the US, Peru, and sub-Saharan Africa.

Design
This is observational cohort study. Enrollments are stratified by clinical symptoms and age group (18-55 and >55) in each country (US, Peru, South Africa, and other Sub-Saharan countries).

Duration per participant
Minimum one visit (1-8 weeks post resolution of COVID-19 symptoms OR 2-10 weeks post most recent positive SARS-CoV-2 test, if asymptomatic/presymptomatic) and optional visits approximately 2 months, 4 months, and 1 year later. Additional follow up visit(s) may be added over time in response to evolving information regarding SARS-CoV-2 infection and the COVID-19 pandemic.

Estimated total study duration
15 months (includes enrollment) with possible extension in response to evolving information regarding SARS-CoV-2 infection and the COVID-19 pandemic.

5 POTENTIAL CONFOUNDERS

Characterizing SARS-CoV-2-specific immunity among convalescent individuals in an observational study is susceptible to measured and unmeasured confounding characteristics of participants (e.g. participants' demographics and medical history including pre-existing conditions, new acute or chronic medical conditions, and concomitant medications). Therefore, analyses involving SARS-CoV-2-specific immune responses will adjust for all measured potential confounders.

6 STATISTICAL ANALYSIS

Data from all enrolled participants will be analyzed. Analyses for primary, secondary and exploratory endpoints will be performed using SAS and R statistical software. No formal multiple comparison adjustments will be employed for multiple primary immunogenicity endpoints, or secondary endpoints across assays. However, multiplicity adjustments will be made for certain immunogenicity assays when the analysis of the assay endpoint generates a collection of hypotheses (e.g., testing multiple peptide pools to determine a positive response) or group comparisons. See below for detail.

Analyses will be performed by region [Americas and sub-Saharan Africa (SSA)]. The comparison between region may be conducted as well. Additional analyses may be performed, limited to participants with laboratory-confirmed SARS-Cov-2 infection.

6.1 Analysis Variables

The analysis variables consist of participant characteristics such as demographics and medical history (including pre-existing conditions, new acute or chronic medical conditions, and concomitant medications); presentations of SARS-CoV-2 infection (including the clinical course of COVID-19); COVID-19 treatments; immunogenicity for primary, secondary, and exploratory objective analyses; and host genetics. In addition, some variables will be derived from these variables. For example, COVID-19 disease severity might be derived from COVID-19 symptoms and treatments collected in the CRF at the enrollment visit as
1: Asymptomatic/presymptomatic
2: Symptomatic but not hospitalized [could divide into supplemental O2 and no supplemental O2]
3: Hospitalized, mild disease, and no supplemental O2
4: Hospitalized, requiring supplemental O2 but no intubation and no ICU
5: Hospitalized- requiring intubation or go into the ICU
6: Hospitalization, ECMO (by definition this is also in ICU)- *likely to be few or no ppts in this group*

In immunological analyses, the time (in days) post first evidence of SARS-CoV-2 infection will be estimated as follows:

For participants with any COVID-19 symptoms: from the first date of COVID-19 symptom onset or the first positive NAAT antigen test date, whichever is earlier, to the samples collection date.

For asymptomatic/presymptomatic participants: from the first positive NAAT/antigen test date to the sample collection date.

6.2 General Approach for Immunological Data

Immune response rates will be analyzed by tabulating the frequency by antigen and group [e.g. enrollment group, pre-existing medical conditions (Yes and No), and severity] at each visit (if applicable) and their corresponding 95% confidence interval will be estimated using the score test method (Agresti & Coull, 1998).

Immune response magnitudes [e.g., percentage of cytokine-secreting cells from the intracellular cytokine staining (ICS) assay] will be summarized using graphical and descriptive statistics. Boxplots will be used to display immune response magnitude by antigen and group [e.g. enrollment group, pre-existing medical conditions (Yes and No), and severity] at each visit (if applicable).

For each immune readout at the enrollment visit, we will investigate any associations between outcome response rate/magnitude and participants' profile covariates of interest such as COVID-19 symptom/severity, demographics (age, sex, race/ethnicity, BMI), pre-existing medical conditions (e.g. hypertension, COPD/emphysema/Asthma, diabetes, immune system disorder and HIV/AIDS), etc. We will run multivariate logistic (for response rates) or linear (for regression magnitudes) models including all covariates of interest. We will also run univariate model for each covariate as a reference. Both univariate and multivariate models will adjust for time post first SARS-CoV-2 positive evidence and country (Peru vs US in Americas analysis; Other SSA countries vs South Africa). We will also perform similar analyses within subgroups of participants without clinical spectrums and outcomes (G1 and G2). The comparisons between enrollment groups will include

- between age groups (18-55 and >55) within asymptomatic (G1A vs G1B)
- between age groups (18-55 and >55) within symptomatic (G1C vs G1D)
- between age groups (18-55 and >55) within hospitalization (G2A vs G2B)

- between asymptomatic and symptomatic within age 18-55 (G1A vs G1C)
- between asymptomatic and symptomatic within age >55 (G1B vs G1D)
- between asymptomatic and hospitalized within age 18-55 (G1A vs G2A)
- between asymptomatic and hospitalized within age >55 (G1B vs G2B)
- between symptomatic and hospitalized within age 18-55 (G1C vs G2A)
- between symptomatic and hospitalized within age >55 (G1D vs G2B)

This restricted analysis is considered as a sensitivity analysis for the full group.

Additional subgroups analyses may also be performed.

For each immune readout, Holm-Bonferroni procedure (Holm, 1979) will be used to calculate adjusted p-values for multiplicity testing between groups to control the family-wise type I error rate for hypothesis-testing. The Benjamini-Hochberg procedure (Benjamini & Hochberg, 1995) will be used to calculate adjusted p-values for multiplicity testing within each assay readout to control the false discovery rate (FDR) for hypothesis-generating.

For longitudinal immune responses analyses, linear mixed models or generalized linear mixed models will be used to compare groups while accounting for correlations of immune response between visits within participants. The models will adjust for time post first SARS-CoV-2 positive evidence and other potential confounders (e.g. age, sex assigned at birth, race/ethnicity, BMI, and pre-existing medical conditions) as appropriate.

When a substantial amount of immunogenicity data are missing (e.g., more than 20% of participants at a given follow up visit), methods that require the missing completely at random (MCAR) assumption may give misleading results. In this situation, analyses of immunogenicity endpoints will be performed using parametric generalized linear models, fitted using the method of maximum likelihood. These methods provide reliable estimates and inferences under parametric modeling assumptions when data are missing at random (MAR). Left- and/or right-censored immunological outcome variables will be analyzed using Hughes' linear mixed effects model (Hughes, 1999) to accommodate censoring.

Spearman correlations between immune response variables will be calculated. Covariate-adjusted Spearman correlations will be also calculated adjusting for covariates (e.g. time post first SARS-CoV-2 positive evidence, age, sex assigned at birth, race/ethnicity, BMI) using the covariate-adjusted Spearman rank correlation method (Liu, Li, Wanga, & Shepherd, 2018). To visualize multivariate immune response variables, we may use dimensionality reduction techniques (e.g., principal component analysis, multidimensional scaling, Uniform Manifold Approximation and Projection) and perform unsupervised learning (e.g., k-means, model-based clustering) to identify subgroups of participants with similar immune profiles.

6.3 Primary and Secondary Analysis

6.3.1 Primary Objective 1

HVTN labs will identify humoral responses to peptide (using the Peptide Microarray assay) and protein antigens (using Binding Antibody Multiplex Assay, BAMA) derived from SARS-CoV-2 structural proteins and regions of the spike protein not present in vaccines. Upon identification of humoral responses to peptides and protein antigens, the analysis will seek to evaluate humoral responses in convalescent individuals by region (Americas and SSA).

Humoral response rates and magnitudes will be analyzed using the methods described in Section 6.2, “General Approach for Immunological Data”.

To visualize the relationship between humoral response magnitude and disease severity among participants who were/will be potentially enrolled in the study at different stages of infection, we will generate a scatter plot of humoral response magnitudes over time post first positive evidence of SARS-CoV-2 infection, with color-coding for severity. A loess regression line for each severity level (if there are enough data points) will be added. The calculations for severity and time post first SARS-CoV-2 positive evidence are described in Section 6.1, "Analysis Variables".

The positivity calls and lists of tables and figures will be detailed in Sec 8 "Assay-Specific Tables and Figures for Protocol Team Reports".

6.3.2 Primary Objective 2

HVTN labs will develop and formally qualify a suite of immunologic assays and reference reagents that permit detailed interrogations of the immune response to SARS-CoV-2 infection, in preparation for similar assessments of vaccine-elicited immune responses and immune-therapeutics. Immune responses include SARS-CoV-2-specific binding antibodies measured by BAMA, neutralizing antibodies (nAbs), ADCC, SARS-CoV-2-specific CD4+ and CD8+ T-cell responses measured by ICS, and SARS-CoV-2-specific memory B cell phenotypes. Similarly to the analyses of humoral responses in Primary Objective 1, all immune response readouts will be analyzed using the methods described in Section 6.2, "General Approach for Immunological Data".

We will generate a scatter plot of immune response magnitudes over time post first SARS-CoV-2 positive evidence, with color-coding for severity. A loess regression line for each severity level (if there are enough data points) will be added.

The positivity calls and lists of tables and figures will be detailed in Sec 8 "Assay-Specific Tables and Figures for Protocol Team Reports" for each assay.

6.3.3 Primary Objective 3

Primary objective 3 will seek to identify immune markers of COVID-19 disease severity and duration in different demographic groups (e.g., age, sex assigned at birth) and in people with different medical histories, including pre-existing conditions [e.g., diabetes, chronic obstructive pulmonary disease (COPD)], new acute or chronic medical conditions, and concomitant medications.

For each immune readout (response rate and magnitude), we will further test its association with disease severity and interaction with age/sex/pre-existing medical conditions adjusting for potential confounders (e.g. age, sex assigned at birth, race/ethnicity, BMI, pre-existing medical conditions) in a logistic/linear regression.

We may apply a machine learning method, Super Learner (Van Der Laan, Polley, & Hubbard, 2007), to predict disease severity from immune responses (rates and magnitudes), participant demographics, and pre-existing medical conditions. A Super Learner is an ensemble of several algorithms such as generalized linear regression (SL.glm), stepwise regression (SL.step), generalized additive models (SL.gam), a generalized linear model via penalized maximum likelihood (SL.glmnet), multiple adaptive regression spline procedure (SL.polymars), and random forest procedure (SL.randomForest). If the number of predictors is large, Lasso regression will be used to screen out variables with zero association coefficients. The prediction performances will be evaluated by cross-validated mean square error (CV-MSE) and cross-validated R^2 (CV-R^2). We will use the vimp R package to estimate the importance for each predictor in the final prediction model (Williamson Gilbert, Simon 2020).

We will also build heatmaps to visualize the relationships between multiple immune responses and perform hierarchical clustering to identify subgroups of participants sharing similar immune

response profiles. Hierarchical clustering is done for both the rows (participants) and the columns (scaled immune responses) of the matrix represented on the heatmap. Various distances will be considered to build these visualizations, including the Euclidean and Mahanalobis distances. Participants (rows) will be annotated in accordance with their group/disease severity, sex assigned at birth, age, medical history (e.g. hypertension, diabetes, any immune system disorder, HIV, ever smoked cigarettes or marijuana). Immune responses (columns) will be annotated by endpoint.
We may also build heatmaps to visualize correlations between immune responses. Spearman correlations between immune response variables will be calculated. Covariate-adjusted Spearman correlations adjusting for covariates (e.g. age, sex assigned at birth, race/ethnicity, BMI, medical history, time post first SARS-CoV-2 positive evidence) will be also calculated using the covariate-adjusted Spearman rank correlation method (Liu et al., 2018).

6.3.4 Primary Objective 4

Primary Objective 4 will seek to characterize presentations of SARS-CoV-2 infection, including the clinical course of COVID-19, among convalescent individuals.

Presentations of SARS-CoV-2 infection will be measured by COVID-19 symptoms that might be grouped by system [General; Head, Eyes, Ears, Nose, and Throat (HEENT); Respiratory; Cardiac; Gastrointestinal (GIT); Central Nervous System (CNS); Skin].

Table 1. COVID-19 symptom systems

General	HEENT	Respiratory	Cardiac	GIT	CNS		Skin
Arthralgia<br>Chills<br>New or increased Fatigue<br>Fever<br>Malaise<br>Myalgia<br>Weakness	Nasal congestion<br>Rhinorrhea<br>Sore throat<br>Throat congestion	Chest pain<br>Cough<br>Dyspnea with exertion<br>Dyspnea at rest<br>Unable to sleep lying down<br>Hemoptysis<br>Increased sputum production<br>Shortness of breath<br>Acute respiratory distress syndrome	Acute cardiac injury<br>Arrhythmia<br>Shock	Abdominal pain<br>Acute liver failure<br>Anorexia<br>Belching<br>Diarrhoea<br>Dysgeusia<br>Nausea<br>Vomiting	Altered mental status / confusion<br>Anosmia / Hyposmia<br>Ataxia<br>Bilateral leg stiffening<br>Conjunctival congestion<br>Dizziness<br>Features of Guillain Barre<br>Gustatory dysfunction<br>Hallucinations<br>Headache	Hypersomnolence<br>Hypogeusia<br>Irritability<br>Loss of consciousness<br>Neuralgias<br>Ophthalmoplegia<br>Visual agnosia<br>Seizure<br>Stroke	Skin tingling<br>Rash

Note: the table includes some symptoms that are not specifically listed in the COVID-19 symptom CRF; these symptoms might be categorized as “Other” in the COVID-19 symptom CRF.

We will use heatmaps and hierarchical clustering to investigate whether subgroups of participants, defined by demographic characteristics and medical history, share similar clusters of symptoms and/or whether the frequency of these clusters differs across participant subgroups. Euclidean distance will be used as the distance measure. Hierarchical clustering is done of both the rows (participants) and the columns [COVID-19 symptoms (Yes vs No)] of the data matrix. Participants (rows) will be annotated by group/severity, sex assigned at birth, age, medical history (e.g. hypertension, diabetes, any immune system disorder, ever smoked cigarettes or marijuana). Symptoms (columns) will be annotated by symptom type (e.g. system).

6.3.5 Secondary Objectives 1 and 2

The proportion of participants with detectable (via NAAT) SARS-CoV-2 viral RNA levels in nasopharyngeal, nasal swab, or saliva cup samples will be tabulated by visit, enrollment group, and region. SARS-CoV-2 viral loads among participants with detectable virus will be summarized and graphically displayed by visit, group, and region. The proportion of participants with detectable SARS-CoV-2 viral RNA and the viral load will be compared between groups both by continent and by region using regression methods described in Section 6.2.

Similarly, the proportions of detectable SARS-CoV-2-specific binding antibodies and the magnitudes of SARS-CoV-2-specific binding antibodies in the nasal cavity will be tabulated and graphically displayed by visit, group, and region.

6.4 Exploratory Analysis

6.4.1 Exploratory Objective 1

Host genotypes (e.g., HLA, FcR, ACE-2, and TMPRSS2 gene variants) will be summarized both by continent and by region. Clinical outcomes (e.g. COVID-19 severity and hospitalization) and immune responses will be compared between host genotype groups while adjusting for covariates (e.g. age, sex assigned at birth, race/ethnicity, pre-existing medical conditions) using the general approaches described in Section 6.2. To account for linkage disequilibrium/correlation between host genotypes, the resampling-based multiplicity adjustment procedures described in (Ge et al., 2003) will be used.

6.4.2 Exploratory Objectives 2-5

We will characterize humoral and cellular responses such as pro-inflammatory cytokine release, cross-reactivity of immune responses with other pathogens, or evaluate other immune responses associated with SARS-CoV-2 infection measured by additional assays. We will calculate covariate-adjusted Spearman’s rank correlation coefficients to characterize the relationships between immune responses or use the methods described in Section 6.2 to characterize and compare immune responses.

6.5 Analyses Prior to End of Study

A statistical analysis of primary immunogenicity endpoints will be performed when all participants in each region (Americas/SSA) have completed the baseline visit (V1) and all data are available. Descriptive analyses of primary/secondary immunogenicity endpoints may also be performed when partial data become available in each region. However, for a secondary or exploratory immunogenicity endpoint, such analyses may only take place after at least one of the primary immunogenicity endpoints of the same class (humoral or cell-mediated) (or, if no primary endpoint of the same class, at least one of the primary immunogenicity endpoints) reaches the aforementioned threshold. The Laboratory Program will review the analysis report prior to distribution to the protocol chairs, DAIDS, and other key HVTN members and investigators. Distribution of reports will be limited to those with a need to know for the purpose of informing future trial-related decisions. The HVTN leadership must approve any other requests for immunogenicity analyses prior to the end of the scheduled follow-up visits.

7 STUDY PARTICIPANT CHARACTERISTICS

Categorical variables of participants’ characteristics and medical history, etc., at the enrollment visit (V1) will be tabulated by enrollment group (or age category or sex assigned at birth specified below) and country. They will also be tabulated by region and compared between two regions (Americas and SSA) using the chi-square test. The distribution of continuous variables of

participants' characteristics and medical history, etc., at the enrollment visit will be summarized by enrollment group and compared between regions using the T-test or F-test.

Screening outcome and study termination will be tabulated overall and by region (US, Peru, and South Africa, and other SSA countries).

7.1 List of Tables

- Screening outcome by region
- Study termination by region
- Specimen collection by type, enrollment group, visit time and region
- Participants' demographics by enrollment group and country and by region

  Age will be summarized as a categorical variable (18-55 and >55 and 10 year categories) as well as a continuous variable.
- Medical history and concomitant medications by enrollment group and country and by region
- COVID-19 treatment and hospitalization by age group (18-55 and 55+) and region and by sex assigned at birth and region
- Presentations of SARS-CoV-2 infection (COVID-19 symptoms and duration) for each symptom and system by age group (18-55 and 55+) and region and by sex assigned at birth and region
- COVID-19 severity by age group (18-55 and 55+) and region and by sex assigned at birth and region
- SARS-CoV-2 exposure by enrollment group and region
- SARS-CoV-2 test results by enrollment group and region
- Time (in Weeks) post SARS-CoV-2 positive group by enrollment group and region
- Pre-existing medical conditions by severity and region

List of Graphs

- Boxplot of time (in days) post first SARS-CoV-2 infection evidence by enrollment group/severity and region

8 ASSAY-SPECIFIC TABLES AND FIGURES FOR PROTOCOL TEAM REPORTS

8.1 Binding Antibody

IgA and IgG binding antibodies and subtype IgG1 and IgG3 antibodies to SARS-CoV-2-specific antigens (TBD) will be measured using BAMA. BAMA assays will be performed for all samples from all participants at Visit 1 (Month 0, baseline) and all available samples at follow-up visits 2-4. Serum SARS-CoV-2-specific IgA/IgG, IgG1, IgG3 responses (dilution TBD) to the same SARS-CoV-2 antigens will be measured on a Bio-Plex instrument (Bio-Rad). The Bioplex software provides 2 readouts: a background-subtracted mean fluorescence intensity (MFI), where background refers to a plate level control (i.e., a blank well run on each plate), and a concentration based on a standard curve. Positive responses at each visit will be called if the MFI minus blank values are >= antigen-specific cutoff (will be provided by the lab). MFIs minus blank (or net MFI) are used to summarize the magnitude at a given time-point.

If sufficient immunogenicity is observed, samples may be titrated to calculate antibody titrations. Titrations are quantified by EC50/AUC.

The descriptive and association analyses described in Section 6.2., "General Approached for Immunological Data", will be performed.

8.1.1 List of Tables

- Response rate table by isotype, antigen, visit, group/severity/pre-existing medical conditions, and region.
- Summary statistics (i.e., min, mean, median, max) among responders by isotype, antigen, visit, group/severity/pre-existing medical conditions, and region.
- Associations table for each readout (response rates and magnitudes).
- If titrations are measured, a table of geometric mean titrations by isotype, antigen, visit, group/severity/pre-existing medical conditions, and region
- If titrations are measured, a table of associations (described in Section 6.2) for titration.

8.1.2 List of Graphs

- Bar chart of binding antibody response rates and boxplots of binding antibody magnitudes (in net MFI or microgram/mL or mAb equivalence units) by specimen type, isotype, antigen, visit, group/severity/pre-existing medical conditions, continent and region. The default layout will be used.
- Scatter plot of binding antibody magnitudes against time post first SARS-CoV-2 positive evidence with color-coding for COVID-19 severity, a loess regression line, and 95% confidence bands by specimen type, isotype, antigen, and region.
- If titrations are measured, boxplots of binding titration (EC50/AUC), by specimen type, isotype, antigen, visit, group/severity/pre-existing medical conditions, continent and region.

8.2 Neutralizing Antibody

Neutralizing antibodies against SARS coronaviruses will be measured in 293T/ACE2 cells as a function of a reduction in Tat-induced luciferase reporter gene expression after a single round of infection with molecularly cloned, Spike-pseudotyped viruses. The 293T cell line is a human embryonic kidney cell line. The 293T/ACE2 cells were made by transduction of ACE2 into 293T cells. They are permissive to infection by strains of betacoronaviruses, including SARS-CoV-1 and SARS-CoV-2 Spike-pseudotyped viruses. Expression of the reporter gene is induced in trans by the viral Tat protein soon after infection. Luciferase activity is quantified by measuring luminescence and is directly proportional to the number of infectious virus particles present in the initial inoculum. The assay is performed in 96-well culture plates for high throughput capacity. The use of a clonal cell population provides enhanced precision and uniformity. The 293T/ACE2 assay is still in development and is being formally optimized and validated for single-round infection with Spike-pseudotyped viruses produced by transfection in 293T cells.
Neutralizing antibody titer [50% and 80% inhibitory dose (ID50, ID80)] will be defined as the serum dilution (ID50, ID80) that reduces relative luminescence units (RLU) by 50% or 80% relative to the RLU in virus-only positive control wells after subtraction of background RLU in negative control wells. Titers against SARS-CoV-2.D614G will be measured in 293T/ACE2 cells. MPI (maximum percent inhibition) will be also measured.
Positivity calls will be an ID50/ID80 value >10 or >20, depending on the starting dilution of the sample, which is the lower limit of detection of the assay.

The descriptive and association analyses described in Section 6.2., “General Approached for Immunological Data”, will be performed.

8.2.1 List of Tables

- Response rate table by virus, visit, group/severity/pre-existing medical conditions, and region.
- Summary statistics (i.e., min, mean, median, max) among responders by virus, visit, group/severity/pre-existing medical conditions, and region.
- Table of all association/comparisons of nAb response rate and titer by region.

8.2.2 List of Graphs

- Bar chart of nAb response rates and boxplots of nAb titer by virus, visit, group/severity/pre-existing medical conditions, and region.
- Scatter plot of nAb titer against time post first SARS-CoV-2 positive evidence with color-coding for COVID-19 severity, a loess regression line, and 95% confidence bands by virus and region.

8.3 CD4 and CD8 T Cell

Flow cytometry was used to examine SARS-CoV-2 specific CD4+ and CD8+ T-cell responses using an intracellular cytokine staining (ICS) assay, validated for selected markers and qualified for other markers. A 27-color panel (experiment Assay ID 117; analysis plan 51 version 2.0) was used (Horton et al., 2007)(De Rosa, Carter, & McElrath, 2012).

The SARS-CoV-2-specific antigens evaluated were COV2 CON S1, COV2 CON S2, COV2 E COV2 M (E and M combined for stimulation), COV2 N, COV2 ORF3a6, and COV2 ORF7a7b8.

Previously cryopreserved specimens were stimulated with the synthetic peptide pools. As a negative control, cells were incubated with DMSO, the diluent for the peptide pools. As a positive control, cells were stimulated with a polyclonal stimulant, staphylococcal enterotoxin B (SEB). There were no replicates except for the negative control, which had two replicates. As an additional internal control, cells were stimulated with a CMVpp65 peptide pool.

Several criteria were used to determine if data from an assay were acceptable and could be statistically analyzed. After sample thawing and overnight incubation, the viability of the PBMC must have been 66% or greater for testing to have proceeded. If it was not, a new aliquot for that sample was thawed for testing. If the PBMC viability of the second thawed aliquot was below this threshold, the ICS assay was not performed and no data were reported to the statistical center for that sample. No negative control acceptance criteria were applied.

The total numbers of CD4+ and CD8+ T cells must also have exceeded certain thresholds. If the number of CD8+ T cells was < 5,000 or CD4+ T cells was < 10,000 for any of the SARS-CoV-2 peptide pools or for one of the negative control replicates for a particular sample, data for that stimulation were filtered. If both negative control replicates failed for number of T cells, the sample was retested. If one negative control replicate failed for number of T cells, the negative control replicate with sufficient cells was used. COV2 CON S1 and COV2 CON S2 were considered the key antigens and if T cells were below the thresholds for either of these

stimulation conditions, the sample was repeated. Samples were not repeated if T cells were below the thresholds for the other SARS COV2 antigens.
The following marker combinations will be reported as primary and secondary markers and selected functional markers will be included in polyfunctionality COMPASS analysis (Table 2).

Table 2. Listing of primary and secondary functional T cell markers

T-cell subset	Functional Marker	Priority	Include in COMPASS
CD4+	IFNg_OR_IL2	Primary	
	IFNg_OR_IL2_OR_154	Secondary	
	IFNg+	Secondary	Yes
	IL2+	Secondary	Yes
	TNFa+	Secondary	Yes
	154+	Secondary	Yes
	IL4_AND_154	Secondary	
	IL5_OR_IL13_AND_154	Secondary	
	IL4_OR_IL5_OR_IL13_AND_154	Secondary	
	IL17a+	Secondary	Yes
	GzB+	Only for COMPASS	Yes (constitutive)
	Perf+	Only for COMPASS	Yes (constitutive)
	IL4+	Only for COMPASS	Yes
	IL5_OR_IL13	Only for COMPASS	Yes
CD8+	IFNg_OR_IL2	Primary	
	IFNg+	Secondary	Yes
	IL2+	Secondary	Yes
	TNFa+	Secondary	Yes
	IFNg_OR_IL2_OR_TNFa	Secondary	
	154+	Secondary	Yes
	IL17a+	Secondary	Yes
	GzB+	Only for COMPASS	Yes (constitutive)
	Perf+	Only for COMPASS	Yes (constitutive)

Positive calls will be made using Fisher's exact positivity test for all these markers. But, the descriptive graphs and the associations described in Section 6.2 "General Approach for Immunological Data", will be performed only for IFN-γ and/or IL2 for CD4+ and CD8+ T cells to each of COV2 antigens and any COV2. The CD4+/CD8+ T cell response magnitudes to Any COV2 will be sum of CD4+/CD8+ T cell response magnitudes over all six COV2 antigens. The positive call for CD4+/CD8+ T cell response to Any COV2 will be made if a positive call is made for the CD4+/CD8+ T cell response to at least one of the six COV2 antigens.

The COMPASS (Combinatorial Polyfunctionality analysis of Antigen-Specific T-cell Subsets) statistical framework (Lin et al., 2015) will be used to perform joint modelling of multiple T-cell subsets (specified for COMPASS analysis in Table 2) of different cytokine combinations. The functionality score (FS) and the polyfunctionality score (PFS) will be used to summarize the multi-parameter ICS responses.

8.3.1 List of Tables (for IFN-γ and/or IL2 for CD4+ and CD8+ T cells)

- Response rate table by T-cell subset, antigen, visit, group/severity/pre-existing medical conditions, and region.
- Summary statistics (i.e., min, mean, median, max) among responders by T-cell subset antigen, visit, group/severity/pre-existing medical conditions, and region.
- Table of all association/comparisons of T-cell response rate and magnitude by T-cell subset, antigen, and region.

8.3.2 List of Graphs (for IFN-γ and/or IL2 for CD4+ and CD8+ T cells)

- Bar chart of T-cell response rates and boxplots of T-cell response magnitude by T-cell subset, antigen, visit, group/severity/pre-existing medical conditions, and region.
- Scatter plot of T-cell response magnitude against time post first SARS-CoV-2 positive evidence with color-coding for COVID-19 severity, a loess regression line, and 95% confidence bands by T-cell subset, antigen and region.

In addition, we will summarize the response rates and magnitudes for all functional markers (those listed in Table 2 above) among all participants.

8.3.3 Additional List of Tables

- Response rate table by T-cell subset, antigen, visit, and functional marker.

8.4 B-Cell Phenotyping (BCP)

SARS-CoV-2-specific B cell responses and B cell phenotypic data will be identified and characterized using fluorescently-labelled recombinant Env proteins (S6P and RBD) in combination with a flow cytometry antibody panel on all available samples from the enrollment visit (V1). The sample will be included in the analysis only if the number of IgD- B cells is equal or greater than 1000 for that sample.

The following 15 SARS-CoV-2-specific B cell frequencies/phenotype will be calculated and evaluated.

	B cell frequencies	Definition
1.	%S+ of total B cells	[Time/S/L/CD19+/CD20+/S6P++]/ [Time/S/L/CD19+/CD20+]*100
2.	%RBD+ of total B cells	[Time/S/L/CD19+/CD20+/RBD+S6P+]/ [Time/S/L/CD19+/CD20+]*100
3.	%S+ IgM+ IgD+ of total B cells	[Time/S/L/CD19+/CD20+/IgD+/IgM+/S6P++]/[Time/S/L/CD19+/CD20+]*100
4.	%S+ IgG+ of total B cells	[Time/S/L/CD19+/CD20+/IgD-/IgG+/S6P++]/[Time/S/L/CD19+/CD20+]*100
5.	%S+ IgA+ of total B cells	[Time/S/L/CD19+/CD20+/IgD-/IgA+/S6P++]/[Time/S/L/CD19+/CD20+]*100
6.	%S+ of Memory B cells	[Time/S/L/CD19+/CD20+/IgD-/S6P++]/ [Time/S/L/CD19+/CD20+/IgD-]*100
7.	%RBD+ of Memory B cells	[Time/S/L/CD19+/CD20+/IgD-/RBD+S6P+]/ [Time/S/L/CD19+/CD20+/IgD-]*100
8.	%RBD+ of %S+ IgG+ B cells	[Time/S/L/CD19+/CD20+/IgD-/IgG+/RBD+S6P+]/[Time/S/L/CD19+/CD20+/IgD-/IgG+/S6P++]*100
9.	%S+ IgG+ of Memory B cells	[Time/S/L/CD19+/CD20+/IgD-/IgG+/S6P++]/ [Time/S/L/CD19+/CD20+/IgD-]*100
10.	%RBD+ IgG+ of Memory B cells	[Time/S/L/CD19+/CD20+/IgD-/IgG+/RBD+S6P+]/ [Time/S/L/CD19+/CD20+/IgD-]*100
11.	%S+ IgA+ of Memory B cells	[Time/S/L/CD19+/CD20+/IgD-/IgA+/S6P++]/ [Time/S/L/CD19+/CD20+/IgD-]*100
12.	%RBD+ IgA+ of Memory B cells	[Time/S/L/CD19+/CD20+/IgD-/IgA+/RBD+S6P+]/ [Time/S/L/CD19+/CD20+/IgD-]*100
13.	%S+ IgM+ of Memory B cells	[Time/S/L/CD19+/CD20+/IgD-/IgM+/S6P++]/ [Time/S/L/CD19+/CD20+/IgD-]*100

14.	%RBD+ IgM+ of Memory B cells	[Time/S/L/CD19+/CD20+/IgD-/IgM+/RBD+S6P+]/ [Time/S/L/CD19+/CD20+/IgD-]*100
15.	%S+ IgM+ of total B cells	[Time/S/L/CD19+/CD20+/IgD-/IgM+/S6P++,Count]/[Time/S/L/CD19+/CD20+,Count]*100

8.4.1 List of Tables

- Summary statistics (i.e., min, mean, median, max) among all participants by B-cell phenotype, overall and by region
- Summary statistics (i.e., min, mean, median, max) by B-cell phenotype and enrollment group/severity/pre-existing medical conditions/days since first SARS-CoV-2 infection evidence (<28 days, 28-<42 days, 42-<56 days, 56+ days), overall and by region.
- Spearman correlations of %S+ of total B cells and %S+ IgG+ total B cells with S-specific CD4+ T cells (CD4+ T cells to COV2 CON S1 and COV2 CON S2); Spearman correlations of %S+ of total B cells, %S+ IgG+ of total B cells, %RBD+ of total B cells, and %RBD+ IgG+ of total B cells with NAb titer (ID50/ID80) among the HIV- participants; and Spearman correlations of %RBD+ of total B cells with %S+ of total B cells, overall and by region

8.4.2 List of Graphs

- Boxplots of B-cell responses by phenotype overall and by region
- Boxplots of B-cell responses by phenotype and group/severity/pre-existing medical conditions/days since first SARS-CoV-2 infection evidence (<28 days, 28-<42 days, 42-<56 days, 56+ days), overall and by region.

9. REFERENCES

Agresti, A., & Coull, B. A. (1998). Approximate Is Better than "Exact" for Interval Estimation of Binomial Proportions. *The American Statistican*, *52*(2), 119–126.

Benjamini, Y., & Hochberg, Y. (1995). Controlling the False Discovery Rate: A Practical and Powerful Approach to Multiple Testing. *Journal of the Royal Statistical Society: Series B (Methodological)*. https://doi.org/10.1111/j.2517-6161.1995.tb02031.x

De Rosa, S. C., Carter, D. K., & McElrath, M. J. (2012). OMIP-014: Validated multifunctional characterization of antigen-specific human T cells by intracellular cytokine staining. *Cytometry Part A*. https://doi.org/10.1002/cyto.a.22218

Ge, Y., Dudoit, S., Speed, T. P., Glonek, G., Solomon, P., Grant, G. R., … Westfall, P. H. (2003). Resampling-based multiple testing for microarray data analysis. *Test*. https://doi.org/10.1007/BF02595811

Holm, S. (1979). A simple sequentially rejective multiple test procedure. *Scandinavian Journal of Statistics*.

Horton, H., Thomas, E. P., Stucky, J. A., Frank, I., Moodie, Z., Huang, Y., … De Rosa, S. C. (2007). Optimization and validation of an 8-color intracellular cytokine staining (ICS) assay to quantify antigen-specific T cells induced by vaccination. *Journal of Immunological Methods*. https://doi.org/10.1016/j.jim.2007.03.002

Hughes, J. P. (1999). Mixed effects models with censored data with application to HIV RNA levels. *Biometrics*, *55*(June), 625–629.

Lin, L., Finak, G., Ushey, K., Seshadri, C., Hawn, T. R., Frahm, N., … Gottardo, R. (2015). COMPASS identifies T-cell subsets correlated with clinical outcomes. *Nature Biotechnology*, *33*(6), 610–616. https://doi.org/10.1038/nbt.3187

Liu, Q., Li, C., Wanga, V., & Shepherd, B. E. (2018). Covariate-adjusted Spearman's rank correlation with probability-scale residuals. *Biometrics*. https://doi.org/10.1111/biom.12812

Van Der Laan, M. J., Polley, E. C., & Hubbard, A. E. (2007). Super learner. *Statistical Applications in Genetics and Molecular Biology*. https://doi.org/10.2202/1544-6115.1309

Williamson, Brian D, Peter B Gilbert, Noah Simon, and M. C. (2020). A Unified Approach for Inference on Algorithm-Agnostic Variable Importance. *Https://Arxiv.Org/Abs/2004.03683*.
