# Supplementary material for: Neutralizing antibody responses over time in demographically and clinically diverse individuals recovered from SARS-CoV-2 infection in the United States and Peru: A cohort study
Source: PLoS Med. 2021 Dec 6;18(12):e1003868. doi: 10.1371/journal.pmed.1003868 (PMC8687542; doi:10.1371/journal.pmed.1003868)
Supplement: S1 Data — Table A. Univariable association of COVID-19 severity, demographics, and medical history with nAb responses in Peru and the US. Table B. Associations of COVID-19 severity (asymptomatic, symptomatic, hospitalized), medical history, and demographics with nAb responses in Peru and the US. Table C. nAb GMT and 95% CI at enrollment visit by participant characteristics (including demographics, medical history, disease severity, and days since SARS-CoV-2 diagnosis). Table D. nAb response rate and 95% CI at enrollment visit by participants’ characteristics (including demographics, medical and smoking history, disease severity, and days since SARS-CoV-2 diagnosis). Table E. Associations of COVID-19 severity by age and sex assigned at birth with nAb ID50/ID80 titer at enrollment after adjusting for participants’ medical history, race/ethnicity, BMI, region, and days since SARS-CoV-2 diagnosis. Table F. Associations of COVID-19 severity, medical history, demographics, and days since SARS-CoV-2 diagnosis at enrollment (V1) with nAb ID50/ID80 titer fold-decline from V1 to V2 among participants with data available at both time points (V1 and V2). Table G. Associations of COVID-19 severity (asymptomatic, symptomatic, hospitalized no O2, hospitalized O2, hospitalized intubation/ICU), medical history, and demographics with nAb responses overall and by region (Peru and the US). Table H. Association of corticosteroid use on nAb responses in symptomatic outpatients and hospitalized individuals after adjusting for age, sex assigned at birth, BMI, diabetes, hypertension, and days since SARS-CoV-2 diagnosis. Table I. nAb GMT and 95% CI at the enrollment visit (V1) and 2-month post-enrollment visit (V2) and GMT ratio (V/V2) among participants with data available at both time points (V1 and V2). Table J. Association of ARBs and/or ACE inhibitor use (n = 49 use and n = 26 no use) and nAb responses, adjusting for COVID-19 severity, age, sex assigned at birth, diabetes, and days since SARS-CoV [file pmed.1003868.s004.docx]

**S1 Data. Supplemental tables A-L.**

| **Table A. Univariable association of COVID-19 severity, demographics, and medical history with neutralizing** **antibody (nAb) responses in Peru and the US** | | | | | | | | | | | |
| --- | --- | --- | --- | --- | --- | --- | --- | --- | --- | --- | --- |
|  | **Response Rate** | | | | **Titer** | | | | | | |
|  | **OR^+^** | **95% CI** | **p-value** | **q-value** | **GMR^*^** | | **95% CI** | | **p-value** | | **q-value** |
| **A. Total** |  |  |  |  |  | |  | |  | |  |
| **ID50** |  |  |  |  |  | |  | |  | |  |
| Symptomatic outpatient vs Asymptomatic | **4.12** | **[1.86, 9.36]** | **0.001** | **0.004** | **5.18** | | **[3, 8.96]** | | **<0.001** | | **<0.001** |
| Hospitalized vs Symptomatic outpatient | **3.01** | **[1.21, 8.34]** | **0.023** | **0.101** | **4.63** | | **[2.99, 7.18]** | | **<0.001** | | **<0.001** |
| Diabetes | 2.17 | [0.71, 8.38] | 0.21 | 0.418 | **2.29** | | **[1.29, 4.06]** | | **0.005** | | **0.017** |
| Hypertension | **0.22** | **[0.09, 0.51]** | **0.001** | **0.004** | 0.63 | | [0.39, 1.01] | | 0.057 | | 0.148 |
| Age (>55 vs 18-55) | 1.81 | [0.89, 3.84] | 0.109 | 0.283 | **2.31** | | **[1.57, 3.42]** | | **<0.001** | | **<0.001** |
| Sex at Birth (Male vs Female) | 0.8 | [0.4, 1.59] | 0.522 | 0.73 | 1.36 | | [0.92, 2.02] | | 0.121 | | 0.225 |
| COPD/Emphysema/Asthma | 1.32 | [0.48, 4.32] | 0.618 | 0.73 | 0.88 | | [0.47, 1.63] | | 0.676 | | 0.843 |
| Current Cigarettes/Marijuana Smoker | 0.72 | [0.28, 2.01] | 0.511 | 0.73 | 0.92 | | [0.46, 1.87] | | 0.826 | | 0.894 |
| Ever Cigarettes/Marijuana Smoker | 0.65 | [0.33, 1.3] | 0.225 | 0.418 | 0.93 | | [0.62, 1.38] | | 0.714 | | 0.843 |
| Non-Hispanic Black vs White | 0.83 | [0.3, 2.38] | 0.722 | 0.782 | 1.05 | | [0.51, 2.15] | | 0.894 | | 0.894 |
| Hispanic/Latino vs Non-His White | 5.96 | [1, 115.58] | 0.105 | 0.283 | 1.61 | | [0.62, 4.18] | | 0.324 | | 0.489 |
| Others vs Non-His White | 1.58 | [0.34, 11.48] | 0.593 | 0.73 | 1.65 | | [0.59, 4.57] | | 0.339 | | 0.489 |
| BMI (≥30 vs <30) | 1.08 | [0.55, 2.19] | 0.826 | 0.826 | 1.38 | | [0.93, 2.05] | | 0.109 | | 0.225 |
| **ID80** |  |  |  |  |  | |  | |  | |  |
| Symptomatic outpatient vs Asymptomatic | **5.29** | **[2.56, 11.33]** | **<0.001** | **<0.001** | **3.44** | | **[2.28, 5.19]** | | **<0.001** | | **<0.001** |
| Hospitalized vs Symptomatic outpatient | **4.77** | **[2.18, 11.33]** | **<0.001** | **0.001** | **4.63** | | **[3.33, 6.43]** | | **<0.001** | | **<0.001** |
| Diabetes | 2.83 | [1.01, 9.16] | 0.062 | 0.179 | **1.93** | | **[1.25, 2.97]** | | **0.003** | | **0.01** |
| Hypertension | 0.51 | [0.23, 1.09] | 0.082 | 0.179 | 0.92 | | [0.64, 1.32] | | 0.654 | | 0.712 |
| Age (>55 vs 18-55) | **2.29** | **[1.22, 4.42]** | **0.011** | **0.049** | **2.11** | | **[1.57, 2.83]** | | **<0.001** | | **<0.001** |
| Sex at Birth (Male vs Female) | 1.22 | [0.66, 2.25] | 0.527 | 0.761 | **1.51** | | **[1.13, 2.02]** | | **0.006** | | **0.016** |
| COPD/Emphysema/Asthma | 0.96 | [0.4, 2.46] | 0.936 | 0.936 | 0.98 | | [0.61, 1.57] | | 0.934 | | 0.934 |
| Current Cigarettes/Marijuana Smoker | 1.55 | [0.6, 4.29] | 0.38 | 0.705 | 1.14 | | [0.67, 1.93] | | 0.635 | | 0.712 |
| Ever Cigarettes/Marijuana Smoker | 0.87 | [0.48, 1.6] | 0.656 | 0.784 | 1.1 | | [0.81, 1.48] | | 0.538 | | 0.712 |
| Non-Hispanic Black vs White | 1.25 | [0.47, 3.43] | 0.664 | 0.784 | 1.27 | | [0.74, 2.17] | | 0.387 | | 0.712 |
| Hispanic/Latino vs Non-His White | 1.61 | [0.47, 6.25] | 0.464 | 0.753 | 1.18 | | [0.57, 2.41] | | 0.657 | | 0.712 |
| Others vs Non-His White | 1.21 | [0.31, 5.42] | 0.791 | 0.857 | 1.2 | | [0.56, 2.6] | | 0.636 | | 0.712 |
| BMI (≥30 vs <30) | 1.77 | [0.95, 3.38] | 0.078 | 0.179 | **1.49** | | **[1.11, 2]** | | **0.009** | | **0.019** |
|  |  |  |  |  |  | |  | |  | |  |
| **B. Peru** |  |  |  |  |  | |  | |  | |  |
| **ID50** |  |  |  |  |  | |  | |  | |  |
| Symptomatic outpatient vs Asymptomatic | 1.79 | [0.38, 7.98] | 0.442 | 0.982 | **3.76** | | **[1.74, 8.15]** | | **0.001** | | **0.004** |
| Hospitalized vs Symptomatic outpatient | 5.04 | [1.1, 35.61] | 0.055 | 0.245 | **6.12** | | **[3.52, 10.63]** | | **<0.001** | | **<0.001** |
| Diabetes |  |  |  |  | 2.01 | | [0.88, 4.6] | | 0.1 | | 0.15 |
| Hypertension | **0.09** | **[0.01, 0.45]** | **0.006** | **0.053** | **0.43** | | **[0.22, 0.86]** | | **0.018** | | **0.04** |
| Age (>55 vs 18-55) | 0.94 | [0.26, 3.9] | 0.931 | 0.992 | **1.97** | | **[1.17, 3.34]** | | **0.012** | | **0.036** |
| Sex at Birth (Male vs Female) | 0.8 | [0.22, 2.85] | 0.725 | 0.992 | 1.45 | | [0.86, 2.43] | | 0.162 | | 0.209 |
| COPD/Emphysema/Asthma | 0.5 | [0.07, 10.11] | 0.546 | 0.982 | **0.33** | | **[0.11, 0.97]** | | **0.046** | | **0.082** |
| Ever Cigarettes/Marijuana Smoker | 0.65 | [0.19, 2.4] | 0.496 | 0.982 | 1.06 | | [0.62, 1.83] | | 0.829 | | 0.829 |
| BMI (≥30 vs <30) | 1.17 | [0.31, 5.63] | 0.83 | 0.992 | 1.25 | | [0.73, 2.14] | | 0.42 | | 0.472 |
| **ID80** |  |  |  |  |  | |  | |  | |  |
| Symptomatic outpatient vs Asymptomatic | 3.19 | [0.97, 10.98] | 0.058 | 0.174 | **3.35** | | **[1.81, 6.19]** | | **<0.001** | | **0.001** |
| Hospitalized vs Symptomatic outpatient | **8.65** | **[2.08, 59.1]** | **0.008** | **0.071** | **5.74** | | **[3.7, 8.9]** | | **<0.001** | | **<0.001** |
| Diabetes |  |  |  |  | 1.87 | | [0.97, 3.6] | | 0.063 | | 0.095 |
| Hypertension | 0.12 | [0.02, 0.61] | 0.016 | 0.073 | 0.68 | | [0.39, 1.18] | | 0.175 | | 0.225 |
| Age (>55 vs 18-55) | 2.07 | [0.64, 8.04] | 0.246 | 0.369 | **1.99** | | **[1.31, 3.02]** | | **0.002** | | **0.005** |
| Sex at Birth (Male vs Female) | 1 | [0.36, 2.88] | 0.993 | 0.993 | 1.49 | | [0.99, 2.24] | | 0.059 | | 0.095 |
| COPD/Emphysema/Asthma | 0.25 | [0.04, 2.18] | 0.167 | 0.3 | **0.4** | | **[0.17, 0.93]** | | **0.036** | | **0.08** |
| Ever Cigarettes/Marijuana Smoker | 0.97 | [0.34, 3] | 0.961 | 0.993 | 1.19 | | [0.77, 1.83] | | 0.434 | | 0.434 |
| BMI (≥30 vs <30) | 2.76 | [0.79, 13.22] | 0.145 | 0.3 | 1.3 | | [0.85, 1.99] | | 0.229 | | 0.258 |
|  |  |  |  |  |  | |  | |  | |  |
| **C. USA** |  |  |  |  |  | |  | |  | |  |
| **ID50** |  |  |  |  |  |  | |  | |  | |
| Symptomatic outpatient vs Asymptomatic | **6.8** | **[2.51, 19.71]** | **<0.001** | **0.003** | **6.56** | **[2.97, 14.49]** | | **<0.001** | | **<0.001** | |
| Hospitalized vs Symptomatic outpatient | 2.48 | [0.79, 8.97] | 0.135 | 0.352 | **3.25** | **[1.6, 6.59]** | | **0.001** | | **0.006** | |
| Diabetes | 2.06 | [0.59, 8.82] | 0.286 | 0.531 | 2.51 | [1.11, 5.69] | | 0.028 | | 0.091 | |
| Hypertension | **0.32** | **[0.11, 0.86]** | **0.026** | **0.149** | 0.8 | [0.41, 1.56] | | 0.507 | | 0.612 | |
| Age (>55 vs 18-55) | **2.61** | **[1.1, 6.63]** | **0.034** | **0.149** | **2.69** | **[1.5, 4.84]** | | **0.001** | | **0.006** | |
| Sex at Birth (Male vs Female) | 0.86 | [0.37, 2.05] | 0.737 | 0.799 | 1.37 | [0.76, 2.48] | | 0.293 | | 0.476 | |
| COPD/Emphysema/Asthma | 1.59 | [0.5, 6.25] | 0.461 | 0.6 | 1.22 | [0.55, 2.7] | | 0.631 | | 0.684 | |
| Current Cigarettes/Marijuana Smoker | 0.58 | [0.2, 1.8] | 0.329 | 0.534 | 1.18 | [0.48, 2.89] | | 0.722 | | 0.722 | |
| Ever Cigarettes/Marijuana Smoker | 0.6 | [0.25, 1.4] | 0.243 | 0.526 | 0.82 | [0.46, 1.48] | | 0.518 | | 0.612 | |
| Non-Hispanic Black vs White | 1.32 | [0.42, 4.44] | 0.638 | 0.755 | 1.47 | [0.64, 3.36] | | 0.367 | | 0.529 | |
| Hispanic/Latino vs Non-His White | 8.37 | [1.26, 174.18] | 0.067 | 0.216 | 1.75 | [0.63, 4.89] | | 0.287 | | 0.476 | |
| Others vs Non-His White | 1.99 | [0.41, 15.25] | 0.437 | 0.6 | 1.92 | [0.63, 5.84] | | 0.253 | | 0.476 | |
| BMI (≥30 vs <30) | 1.09 | [0.48, 2.53] | 0.835 | 0.835 | 1.47 | [0.83, 2.61] | | 0.189 | | 0.476 | |
| **ID80** |  |  |  |  |  |  | |  | |  | |
| Symptomatic outpatient vs Asymptomatic | **7.18** | **[2.77, 20.23]** | **<0.001** | **0.001** | **3.36** | **[1.89, 5.97]** | | **<0.001** | | **<0.001** | |
| Hospitalized vs Symptomatic outpatient | **3.88** | **[1.49, 11.14]** | **0.008** | **0.051** | **3.41** | **[2.04, 5.69]** | | **<0.001** | | **<0.001** | |
| Diabetes | 2.79 | [0.89, 10.1] | 0.094 | 0.306 | **1.9** | **[1.05, 3.44]** | | **0.035** | | **0.092** | |
| Hypertension | 0.79 | [0.33, 1.92] | 0.605 | 0.634 | 1.12 | [0.69, 1.83] | | 0.653 | | 0.707 | |
| Age (>55 vs 18-55) | **2.54** | **[1.2, 5.62]** | **0.017** | **0.076** | **2.17** | **[1.42, 3.33]** | | **<0.001** | | **0.002** | |
| Sex at Birth (Male vs Female) | 1.39 | [0.65, 3.05] | 0.397 | 0.573 | 1.53 | [1, 2.34] | | 0.05 | | 0.108 | |
| COPD/Emphysema/Asthma | 1.28 | [0.48, 3.66] | 0.634 | 0.634 | 1.34 | [0.76, 2.39] | | 0.316 | | 0.456 | |
| Current Cigarettes/Marijuana Smoker | 1.66 | [0.55, 5.4] | 0.38 | 0.573 | 1.43 | [0.75, 2.74] | | 0.281 | | 0.456 | |
| Ever Cigarettes/Marijuana Smoker | 0.81 | [0.38, 1.7] | 0.579 | 0.634 | 1.03 | [0.67, 1.59] | | 0.879 | | 0.879 | |
| Non-Hispanic Black vs White | 1.72 | [0.58, 5.51] | 0.341 | 0.573 | 1.47 | [0.81, 2.69] | | 0.211 | | 0.392 | |
| Hispanic/Latino vs Non-His White | 1.8 | [0.5, 7.57] | 0.388 | 0.573 | 1.19 | [0.56, 2.51] | | 0.652 | | 0.707 | |
| Others vs Non-His White | 1.44 | [0.36, 6.74] | 0.617 | 0.634 | 1.31 | [0.58, 2.95] | | 0.51 | | 0.663 | |
| BMI (≥30 vs <30) | 1.56 | [0.75, 3.34] | 0.238 | 0.573 | **1.62** | **[1.07, 2.44]** | | **0.024** | | **0.077** | |
| ^+^: Odds Ratio ^*^: Geometric Mean Ratio. Region and days since SARS-CoV-2 diagnosis were adjusted in all univariable regression models. In addition, age was adjusted in the model of COVID-19 severity; COVID-19 severity was adjusted in the model of age; and COVID-19 severity and age were adjusted in the models of other variables. | | | | | | | | | | | |

| **Table B. Associations of COVID-19 severity (asymptomatic, symptomatic, hospitalized), medical history, and demographics with nAb responses in Peru and the US.** | | | | | | | | | |  |
| --- | --- | --- | --- | --- | --- | --- | --- | --- | --- | --- |
|  | **Response Rate** | | | | **Titer** | | | |  |  |
|  | **OR** | **95% CI** | **p-value** | **q-value** | **GMR** | **95% CI** | **p-value** | **q-value** |  |  |
| **A. Peru** |  |  |  |  |  |  |  |  |  |  |
| **ID50** |  |  |  |  |  |  |  |  |  |  |
| COVID-19 severity (overall test) | - | - | **0.029** |  | - | - | **<0.001** |  |  |  |
| Symptomatic outpatient vs asymptomatic | 2.03 | [0.37, 11.22] | 0.405 | 0.541 | **4.28** | **[2.02, 9.06]** | **<0.001** | **0.001** |  |  |
| Hospitalized vs symptomatic outpatient | **9.13** | **[1.39, 106.85]** | **0.038** | 0.152 | **4.83** | **[2.72, 8.59]** | **<0.001** | **<0.001** |  |  |
| Diabetes | - | - | - |  | 2.16 | [0.96, 4.85] | 0.064 | 0.106 |  | |
| Hypertension | **0.06** | **[0.01, 0.36]** | **0.004** | **0.032** | **0.36** | **[0.18, 0.72]** | **0.004** | **0.014** |  |  |
| Age (>55 vs 18-55) | 2.95 | [0.53, 26.45] | 0.262 | 0.524 | **1.86** | **[1.07, 3.22]** | **0.029** | **0.057** |  |  |
| Sex assigned at birth (male vs female) | 1.21 | [0.3, 5.15] | 0.791 | 0.791 | 1.52 | [0.91, 2.56] | 0.115 | 0.143 |  |  |
| COPD/emphysema/asthma | 0.2 | [0.02, 4.58] | 0.206 | 0.524 | **0.28** | **[0.1, 0.8]** | **0.019** | **0.047** |  |  |
| Current cigarettes/marijuana smoker | - | - | - |  | 0.34 | [0.1, 1.22] | 0.1 | 0.143 |  | |
| Ever cigarettes/marijuana smoker | 0.51 | [0.11, 2.3] | 0.367 | 0.541 | 1.08 | [0.61, 1.91] | 0.801 | 0.801 |  |  |
| BMI (≥30 vs <30) | 1.43 | [0.33, 8.13] | 0.655 | 0.749 | 1.12 | [0.66, 1.89] | 0.676 | 0.676 |  |  |
| **ID80** |  |  |  |  |  |  |  |  |  |  |
| COVID-19 severity (overall test) | - | - | **0.003** |  | - | - | **<0.001** |  |  |  |
| Symptomatic outpatient vs asymptomatic | 3.82 | [1.05, 15.22] | 0.046 | 0.107 | **3.71** | **[2.04, 6.73]** | **<0.001** | **<0.001** |  |  |
| Hospitalized vs symptomatic outpatient | **17.78** | **[3.02, 202.46]** | **0.005** | **0.032** | **4.57** | **[2.89, 7.23]** | **<0.001** | **<0.001** |  |  |
| Diabetes | - | - | - |  | **1.93** | **[1.01, 3.67]** | **0.047** | **0.086** |  |  |
| Hypertension | **0.08** | **[0.01, 0.45]** | **0.008** | **0.032** | 0.6 | [0.35, 1.03] | 0.065 | 0.086 |  |  |
| Age (>55 vs 18-55) | 6.06 | [1.2, 53.8] | 0.054 | 0.107 | **1.79** | **[1.16, 2.77]** | **0.01** | **0.034** |  |  |
| Sex assigned at birth (male vs female) | 1.35 | [0.43, 4.53] | 0.609 | 0.609 | 1.51 | [1, 2.28] | 0.053 | 0.086 |  |  |
| COPD/emphysema/asthma | 0.12 | [0.01, 1.34] | 0.077 | 0.124 | **0.36** | **[0.16, 0.84]** | **0.019** | **0.048** |  |  |
| Current cigarettes/marijuana smoker | - | - | - |  | 0.39 | [0.14, 1.07] | 0.069 | 0.086 |  |  |
| Ever cigarettes/marijuana smoker | 0.66 | [0.19, 2.32] | 0.51 | 0.583 | 1.19 | [0.76, 1.88] | 0.45 | 0.45 |  |  |
| BMI (≥30 vs <30) | 3 | [0.78, 15.6] | 0.14 | 0.186 | 1.18 | [0.77, 1.78] | 0.448 | 0.45 |  |  |
|  |  |  |  |  |  |  |  |  |  |  |
| **B. USA** |  |  |  |  |  |  |  |  |  |  |
| **ID50** |  |  |  |  |  |  |  |  |  |  |
| COVID-19 severity (overall test) | **-** | **-** | **<0.001** |  | **-** | **-** | **<0.001** |  |  |  |
| Symptomatic outpatient vs asymptomatic | **9.61** | **[2.5, 43.47]** | **0.002** | **0.022** | **10.61** | **[4.21, 26.73]** | **<0.001** | **<0.001** |  |  |
| Hospitalized vs symptomatic outpatient | 2.64 | [0.66, 12.21] | 0.186 | 0.404 | **2.81** | **[1.31, 6.05]** | **0.009** | **0.039** |  |  |
| Diabetes | 3.05 | [0.73, 16.61] | 0.157 | 0.404 | **2.64** | **[1.09, 6.37]** | **0.032** | **0.106** |  |  |
| Hypertension | **0.25** | **[0.07, 0.78]** | **0.021** | **0.092** | 0.53 | [0.26, 1.11] | 0.093 | 0.241 |  |  |
| Age (>55 vs 18-55) | **4.79** | **[1.63, 16.37]** | **0.007** | **0.046** | **3.44** | **[1.79, 6.62]** | **<0.001** | **0.002** |  |  |
| Sex assigned at birth (male vs female) | 1.44 | [0.53, 4.1] | 0.48 | 0.624 | 1.67 | [0.89, 3.13] | 0.113 | 0.246 |  |  |
| COPD/emphysema/asthma | 1.68 | [0.45, 7.84] | 0.469 | 0.624 | 1.34 | [0.59, 3.04] | 0.488 | 0.488 |  |  |
| Current cigarettes/marijuana smoker | 0.95 | [0.26, 3.73] | 0.935 | 0.935 | 1.48 | [0.56, 3.89] | 0.432 | 0.488 |  |  |
| Ever cigarettes/marijuana smoker | 0.58 | [0.2, 1.61] | 0.297 | 0.552 | 0.66 | [0.35, 1.25] | 0.201 | 0.337 |  |  |
| Non-Hispanic Black vs White | 1.21 | [0.35, 4.53] | 0.768 | 0.833 | 1.35 | [0.58, 3.11] | 0.487 | 0.488 |  |  |
| Hispanic/Latino vs Non-His White | 7.01 | [0.97, 157.8] | 0.108 | 0.35 | 1.65 | [0.59, 4.59] | 0.342 | 0.444 |  |  |
| Others vs Non-His White | 1.99 | [0.36, 16.17] | 0.462 | 0.624 | 1.74 | [0.57, 5.33] | 0.336 | 0.444 |  |  |
| BMI (≥30 vs <30) | 1.22 | [0.47, 3.26] | 0.691 | 0.817 | 1.48 | [0.81, 2.71] | 0.207 | 0.337 |  |  |
| **ID80** |  |  |  |  |  |  |  |  |  |  |
| COVID-19 severity (overall test) | **-** | **-** | **<0.001** |  | **-** | **-** | **<0.001** |  |  |  |
| Symptomatic outpatient vs asymptomatic | **16.08** | **[4.52, 68.17]** | **<0.001** | **0.001** | **5.59** | **[2.88, 10.85]** | **<0.001** | **<0.001** |  |  |
| Hospitalized vs symptomatic outpatient | **3.49** | **[1.17, 11.42]** | **0.03** | **0.132** | **2.85** | **[1.65, 4.93]** | **<0.001** | **0.001** |  |  |
| Diabetes | 3.24 | [0.92, 13.52] | 0.083 | 0.27 | 1.76 | [0.94, 3.32] | 0.08 | 0.174 |  |  |
| Hypertension | 0.52 | [0.19, 1.43] | 0.209 | 0.366 | 0.77 | [0.46, 1.3] | 0.335 | 0.435 |  |  |
| Age (>55 vs 18-55) | **3.49** | **[1.46, 8.93]** | **0.007** | **0.043** | **2.42** | **[1.51, 3.86]** | **<0.001** | **0.001** |  |  |
| Sex assigned at birth (male vs female) | 1.7 | [0.73, 4.16] | 0.225 | 0.366 | **1.68** | **[1.07, 2.64]** | **0.026** | **0.084** |  |  |
| COPD/emphysema/asthma | 1.32 | [0.45, 4.22] | 0.619 | 0.67 | 1.51 | [0.84, 2.71] | 0.175 | 0.326 |  |  |
| Current cigarettes/marijuana smoker | 2.52 | [0.7, 9.99] | 0.169 | 0.366 | 1.44 | [0.72, 2.88] | 0.306 | 0.435 |  |  |
| Ever cigarettes/marijuana smoker | 0.59 | [0.25, 1.37] | 0.221 | 0.366 | 0.85 | [0.54, 1.34] | 0.475 | 0.561 |  |  |
| Non-Hispanic Black vs White | 1.52 | [0.48, 5.19] | 0.485 | 0.573 | 1.48 | [0.81, 2.7] | 0.201 | 0.326 |  |  |
| Hispanic/Latino vs Non-His White | 1.77 | [0.47, 7.9] | 0.424 | 0.551 | 1.18 | [0.57, 2.46] | 0.661 | 0.661 |  |  |
| Others vs Non-His White | 1.29 | [0.29, 6.43] | 0.741 | 0.741 | 1.26 | [0.56, 2.82] | 0.573 | 0.621 |  |  |
| BMI (≥30 vs <30) | 1.6 | [0.71, 3.76] | 0.264 | 0.382 | **1.56** | **[1.01, 2.4]** | **0.047** | **0.122** |  |  |
| Note: All Diabetes participants (n=17) and all current smokers (n=7) in Peru had positive nAb ID50/ID80 response. Diabetes and current cigarettes/marijuana smoker were excluded in the response rate analysis. Days since SARS-CoV-2 diagnosis was adjusted in the model. Bold values are significant. | | | | | | | | | |  |

| **Table C. NAb geometric mean titer (GMT) and 95% CI at enrollment visit by participant characteristics (including demographics, medical history, disease severity, and days since SARS-CoV-2 diagnosis).** | | | | | | |
| --- | --- | --- | --- | --- | --- | --- |
|  | **ID50** | | | **ID80** | | |
| **Characteristic** | **Total (n=329)** | **Peru (n=162)** | **USA (n=167)** | **Total (n=329)** | **Peru (n=162)** | **USA (n=167)** |
| Overall | 499.7 (394.1, 633.5) | 1049.6 (767.6, 1435.1) | 243.2 (176.1, 335.8) | 103.6 (85.5, 125.6) | 202.5 (155, 264.6) | 54.1 (42.6, 68.7) |
| Age |  |  |  |  |  |  |
| 18-55 | 362.1 (266.9, 491.1) | 752.6 (510.8, 1108.8) | 165.2 (107.5, 254) | 78.1 (61.6, 99) | 145.8 (105.3, 202.1) | 40 (29.7, 53.8) |
| >55 | 828.5 (574.6, 1194.7) | 1905.7 (1145.6, 3170.2) | 415.5 (259.5, 665.3) | 161.6 (118.2, 220.9) | 365 (235.9, 564.6) | 82.3 (56, 120.8) |
| Sex assigned at birth |  |  |  |  |  |  |
| Female | 353.9 (259.3, 482.9) | 629.7 (404.4, 980.6) | 219.1 (144.6, 332) | 70.1 (54.7, 89.7) | 120.6 (83.7, 174) | 44.6 (32.7, 60.8) |
| Male | 701.1 (492.3, 998.5) | 1612.8 (1052.9, 2470.4) | 273.9 (164.3, 456.7) | 152.2 (114.6, 202.1) | 313.2 (217.3, 451.3) | 67.5 (46.5, 97.8) |
| Race/ethnicity |  |  |  |  |  |  |
| Hispanic or Latinx | 933.4 (692.2, 1258.7) | 1049.6 (767.6, 1435.1) | 263 (110.9, 623.8) | 178 (137.4, 230.7) | 202.5 (155, 264.6) | 44.2 (20.2, 96.6) |
| Non-Hispanic Black | 168.4 (77.1, 367.8) |  | 168.4 (77.1, 367.8) | 50.4 (29.4, 86.6) |  | 50.4 (29.4, 86.6) |
| Non-Hispanic White | 257.2 (169.1, 391.3) |  | 257.2 (169.1, 391.3) | 55.6 (40.6, 76) |  | 55.6 (40.6, 76) |
| Other | 394.2 (128, 1213.6) |  | 394.2 (128, 1213.6) | 67.1 (27.2, 165.5) |  | 67.1 (27.2, 165.5) |
| BMI |  |  |  |  |  |  |
| <30 | 462.8 (342.9, 624.7) | 885.1 (600.3, 1305.1) | 213.8 (140, 326.3) | 92.4 (72.3, 118.2) | 169.3 (121.2, 236.6) | 44.9 (32.9, 61.3) |
| ≥30 | 568 (383.6, 841.1) | 1537.4 (910.1, 2597.1) | 287.2 (173, 476.8) | 125.5 (92.3, 170.8) | 302.6 (197, 464.7) | 68.7 (47.2, 100) |
| COVID-19 severity at enrollment |  |  |  |  |  |  |
| Asymptomatic | 100.9 (62.3, 163.7) | 210.3 (109.5, 403.6) | 57.9 (30, 112) | 27.7 (20.4, 37.7) | 41.2 (26, 65.3) | 20.5 (13.7, 30.7) |
| Symptomatic | 355.5 (250.1, 505.2) | 571.3 (332.4, 981.7) | 253.3 (160.4, 400) | 68.4 (52.3, 89.3) | 112.1 (73.8, 170.2) | 48 (34.4, 67) |
| Hospitalized | 1543.7 (1129.2, 2110.3) | 2834 (1995.9, 4024) | 624.2 (380.2, 1024.7) | 300.9 (229.1, 395.1) | 537.7 (396.2, 729.7) | 126.6 (83.8, 191.3) |
| Hypertension |  |  |  |  |  |  |
| No | 555.7 (428.5, 720.6) | 1119.8 (810, 1548.2) | 241.4 (166.9, 349.2) | 106.2 (85.5, 131.9) | 202.7 (152.5, 269.3) | 49.2 (37.3, 64.9) |
| Yes | 348.6 (199, 610.7) | 723.2 (251.9, 2075.9) | 247.3 (127.6, 479.5) | 95.6 (62.7, 145.7) | 201.8 (88.8, 458.5) | 67.2 (41.8, 108) |
| COPD/emphysema/asthma |  |  |  |  |  |  |
| No | 525.5 (408, 676.8) | 1096 (794, 1512.9) | 236.7 (166.2, 337.2) | 107.4 (87.6, 131.8) | 209.2 (158.6, 275.9) | 52.1 (40.4, 67.3) |
| Yes | 327 (163, 656.1) | 502.7 (112.3, 2249.4) | 281.8 (122.2, 650) | 76.7 (43, 136.8) | 117.1 (36.8, 372.7) | 66.3 (32.8, 134.1) |
| Diabetes |  |  |  |  |  |  |
| No | 444.6 (344.5, 573.7) | 900 (643.7, 1258.3) | 216.4 (152.3, 307.3) | 94.1 (76.6, 115.5) | 175.6 (132.5, 232.6) | 49.7 (38.2, 64.7) |
| Yes | 1110 (596.8, 2064.7) | 3894.2 (2134.8, 7103.6) | 472.8 (207.3, 1078.4) | 201.2 (119, 340.2) | 685.1 (352.2, 1332.7) | 87.4 (49.1, 155.6) |
| Currently smoke cigarettes or marijuana | |  |  |  |  |  |
| No | 547.3 (428.5, 698.9) | 1115 (809.1, 1536.4) | 258.4 (185, 361) | 110.7 (90.4, 135.5) | 214.3 (162.9, 282) | 55.1 (42.7, 71.2) |
| Yes | 180.6 (73, 446.5) | 275.3 (71.5, 1059.1) | 155.8 (47.6, 509.4) | 49.9 (28.4, 87.4) | 57.9 (23.8, 141.2) | 47.3 (22.7, 98.4) |
| Ever smoked cigarettes or marijuana |  |  |  |  |  |  |
| No | 619.8 (460.6, 834.1) | 1127.8 (790.7, 1608.6) | 251.5 (159.7, 396.1) | 118.6 (92.4, 152.2) | 207.7 (152.4, 283) | 51 (36.2, 71.8) |
| Yes | 374.9 (254.7, 551.7) | 889.2 (463.9, 1704.5) | 236.7 (149.2, 375.4) | 86.6 (64.1, 117.1) | 191.1 (111.5, 327.6) | 56.8 (40.5, 79.7) |
| Days since SARS-CoV-2 diagnosis at enrollment | |  |  |  |  |  |
| <28 | 478.1 (252.9, 903.9) | 634.4 (306.7, 1312) | 204.7 (47.1, 889.4) | 82.2 (51.3, 131.9) | 98.5 (55.5, 174.8) | 47.8 (19.5, 117.2) |
| 28-41 | 751.8 (409.2, 1381) | 2055.5 (1152.8, 3665.1) | 109.3 (38.2, 312.7) | 161.5 (97.5, 267.4) | 368.1 (218.8, 619.2) | 33.3 (15.2, 73) |
| 42-55 | 560 (335.4, 935) | 1315.8 (697.6, 2481.9) | 180.6 (87.8, 371.6) | 115.3 (75.8, 175.4) | 244.5 (141.3, 423.1) | 42.6 (25.4, 71.4) |
| 56+ | 386.7 (281.3, 531.5) | 530.1 (309, 909.4) | 334 (224.9, 496) | 82.2 (64.1, 105.3) | 126.7 (82.6, 194.5) | 67.2 (49.7, 90.7) |

| **Table D. NAb response rate and 95% CI at enrollment visit by participants characteristics (including demographics, medical and smoking history, disease severity, and days since SARS-CoV-2 diagnosis).** | | | |
| --- | --- | --- | --- |
|  | **ID50** | | |
| **Characteristic** | **Total (n=329)** | **Peru (n=162)** | **USA (n=167)** |
| Overall | 281/329 = 85.4% (81.2%, 88.8%) | 150/162 = 92.6% (87.5%, 95.7%) | 131/167 = 78.4% (71.6%, 84.0%) |
| Age |  |  |  |
| 18-55 | 167/201 = 83.1% (77.3%, 87.6%) | 96/104 = 92.3% (85.5%, 96.0%) | 71/97 = 73.2% (63.6%, 81.0%) |
| >55 | 114/128 = 89.1% (82.5%, 93.4%) | 54/58 = 93.1% (83.6%, 97.3%) | 60/70 = 85.7% (75.7%, 92.0%) |
| Sex assigned at birth |  |  |  |
| Female | 140/163 = 85.9% (79.7%, 90.4%) | 68/74 = 91.9% (83.4%, 96.2%) | 72/89 = 80.9% (71.5%, 87.7%) |
| Male | 141/166 = 84.9% (78.7%, 89.6%) | 82/88 = 93.2% (85.9%, 96.8%) | 59/78 = 75.6% (65.1%, 83.8%) |
| Race/ethnicity |  |  |  |
| Hispanic or Latinx | 164/177 = 92.7% (87.8%, 95.7%) | 150/162 = 92.6% (87.5%, 95.7%) | 14/15 = 93.3% (70.2%, 98.8%) |
| Non-Hispanic Black | 24/36 = 66.7% (50.3%, 79.8%) |  | 24/36 = 66.7% (50.3%, 79.8%) |
| Non-Hispanic White | 82/103 = 79.6% (70.8%, 86.3%) |  | 82/103 = 79.6% (70.8%, 86.3%) |
| Other | 11/13 = 84.6% (57.8%, 95.7%) |  | 11/13 = 84.6% (57.8%, 95.7%) |
| BMI |  |  |  |
| <30 | 177/206 = 85.9% (80.5%, 90.0%) | 103/112 = 92.0% (85.4%, 95.7%) | 74/94 = 78.7% (69.4%, 85.8%) |
| ≥30 | 104/123 = 84.6% (77.1%, 89.9%) | 47/50 = 94.0% (83.8%, 97.9%) | 57/73 = 78.1% (67.3%, 86.0%) |
| COVID-19 severity at enrollment |  |  |  |
| Asymptomatic | 43/65 = 66.2% (54.0%, 76.5%) | 24/28 = 85.7% (68.5%, 94.3%) | 19/37 = 51.4% (35.9%, 66.5%) |
| Symptomatic | 113/132 = 85.6% (78.6%, 90.6%) | 49/55 = 89.1% (78.2%, 94.9%) | 64/77 = 83.1% (73.2%, 89.9%) |
| Hospitalized | 125/132 = 94.7% (89.5%, 97.4%) | 77/79 = 97.5% (91.2%, 99.3%) | 48/53 = 90.6% (79.8%, 95.9%) |
| Hypertension |  |  |  |
| No | 226/254 = 89.0% (84.5%, 92.3%) | 131/138 = 94.9% (89.9%, 97.5%) | 95/116 = 81.9% (73.9%, 87.8%) |
| Yes | 55/75 = 73.3% (62.4%, 82.0%) | 19/24 = 79.2% (59.5%, 90.8%) | 36/51 = 70.6% (57.0%, 81.3%) |
| COPD/emphysema/asthma |  |  |  |
| No | 251/294 = 85.4% (80.9%, 89.0%) | 142/153 = 92.8% (87.6%, 95.9%) | 109/141 = 77.3% (69.7%, 83.4%) |
| Yes | 30/35 = 85.7% (70.6%, 93.7%) | 8/9 = 88.9% (56.5%, 98.0%) | 22/26 = 84.6% (66.5%, 93.8%) |
| Diabetes |  |  |  |
| No | 243/287 = 84.7% (80.0%, 88.4%) | 133/145 = 91.7% (86.1%, 95.2%) | 110/142 = 77.5% (69.9%, 83.6%) |
| Yes | 38/42 = 90.5% (77.9%, 96.2%) | 17/17 = 100.0% (81.6%, 100.0%) | 21/25 = 84.0% (65.3%, 93.6%) |
| Currently smoke cigarettes or marijuana |  |  |  |
| No | 262/302 = 86.8% (82.5%, 90.1%) | 143/155 = 92.3% (87.0%, 95.5%) | 119/147 = 81.0% (73.9%, 86.5%) |
| Yes | 19/27 = 70.4% (51.5%, 84.2%) | 7/7 = 100.0% (64.6%, 100.0%) | 12/20 = 60.0% (38.7%, 78.1%) |
| Ever smoked cigarettes or marijuana |  |  |  |
| No | 167/188 = 88.8% (83.5%, 92.6%) | 106/113 = 93.8% (87.8%, 97.0%) | 61/75 = 81.3% (71.1%, 88.5%) |
| Yes | 114/141 = 80.9% (73.6%, 86.5%) | 44/49 = 89.8% (78.2%, 95.6%) | 70/92 = 76.1% (66.4%, 83.6%) |
| Days since SARS-CoV-2 diagnosis at enrollment |  |  |  |
| <28 | 26/28 = 92.9% (77.3%, 98.0%) | 20/21 = 95.2% (77.3%, 99.2%) | 6/7 = 85.7% (48.7%, 97.4%) |
| 28-41 | 59/70 = 84.3% (74.0%, 91.0%) | 44/46 = 95.7% (85.5%, 98.8%) | 15/24 = 62.5% (42.7%, 78.8%) |
| 42-55 | 71/86 = 82.6% (73.2%, 89.1%) | 44/49 = 89.8% (78.2%, 95.6%) | 27/37 = 73.0% (57.0%, 84.6%) |
| 56+ | 125/145 = 86.2% (79.7%, 90.9%) | 42/46 = 91.3% (79.7%, 96.6%) | 83/99 = 83.8% (75.3%, 89.8%) |
|  | **ID80** | | |
| **Characteristic** | **Total (n=329)** | **Peru (n=162)** | **USA (n=167)** |
| Overall | 250/329 = 76.0% (71.1%, 80.3%) | 141/162 = 87.0% (81.0%, 91.4%) | 109/167 = 65.3% (57.8%, 72.1%) |
| Age |  |  |  |
| 18-55 | 144/201 = 71.6% (65.0%, 77.4%) | 87/104 = 83.7% (75.4%, 89.5%) | 57/97 = 58.8% (48.8%, 68.0%) |
| >55 | 106/128 = 82.8% (75.3%, 88.4%) | 54/58 = 93.1% (83.6%, 97.3%) | 52/70 = 74.3% (63.0%, 83.1%) |
| Sex assigned at birth |  |  |  |
| Female | 119/163 = 73.0% (65.7%, 79.2%) | 62/74 = 83.8% (73.8%, 90.5%) | 57/89 = 64.0% (53.7%, 73.2%) |
| Male | 131/166 = 78.9% (72.1%, 84.4%) | 79/88 = 89.8% (81.7%, 94.5%) | 52/78 = 66.7% (55.6%, 76.1%) |
| Race/ethnicity |  |  |  |
| Hispanic or Latinx | 151/177 = 85.3% (79.3%, 89.8%) | 141/162 = 87.0% (81.0%, 91.4%) | 10/15 = 66.7% (41.7%, 84.8%) |
| Non-Hispanic Black | 21/36 = 58.3% (42.2%, 72.9%) |  | 21/36 = 58.3% (42.2%, 72.9%) |
| Non-Hispanic White | 69/103 = 67.0% (57.4%, 75.3%) |  | 69/103 = 67.0% (57.4%, 75.3%) |
| Other | 9/13 = 69.2% (42.4%, 87.3%) |  | 9/13 = 69.2% (42.4%, 87.3%) |
| BMI |  |  |  |
| <30 | 153/206 = 74.3% (67.9%, 79.8%) | 94/112 = 83.9% (76.0%, 89.6%) | 59/94 = 62.8% (52.7%, 71.9%) |
| ≥30 | 97/123 = 78.9% (70.8%, 85.2%) | 47/50 = 94.0% (83.8%, 97.9%) | 50/73 = 68.5% (57.1%, 78.0%) |
| COVID-19 severity at enrollment |  |  |  |
| Asymptomatic | 30/65 = 46.2% (34.6%, 58.1%) | 18/28 = 64.3% (45.8%, 79.3%) | 12/37 = 32.4% (19.6%, 48.5%) |
| Symptomatic | 98/132 = 74.2% (66.2%, 80.9%) | 46/55 = 83.6% (71.7%, 91.1%) | 52/77 = 67.5% (56.5%, 76.9%) |
| Hospitalized | 122/132 = 92.4% (86.6%, 95.8%) | 77/79 = 97.5% (91.2%, 99.3%) | 45/53 = 84.9% (73.0%, 92.2%) |
| Hypertension |  |  |  |
| No | 198/254 = 78.0% (72.5%, 82.6%) | 122/138 = 88.4% (82.0%, 92.7%) | 76/116 = 65.5% (56.5%, 73.5%) |
| Yes | 52/75 = 69.3% (58.2%, 78.6%) | 19/24 = 79.2% (59.5%, 90.8%) | 33/51 = 64.7% (51.0%, 76.4%) |
| COPD/emphysema/asthma |  |  |  |
| No | 225/294 = 76.5% (71.4%, 81.0%) | 134/153 = 87.6% (81.4%, 91.9%) | 91/141 = 64.5% (56.4%, 72.0%) |
| Yes | 25/35 = 71.4% (54.9%, 83.7%) | 7/9 = 77.8% (45.3%, 93.7%) | 18/26 = 69.2% (50.0%, 83.5%) |
| Diabetes |  |  |  |
| No | 214/287 = 74.6% (69.2%, 79.3%) | 124/145 = 85.5% (78.9%, 90.3%) | 90/142 = 63.4% (55.2%, 70.9%) |
| Yes | 36/42 = 85.7% (72.2%, 93.3%) | 17/17 = 100.0% (81.6%, 100.0%) | 19/25 = 76.0% (56.6%, 88.5%) |
| Currently smoke cigarettes or marijuana |  |  |  |
| No | 232/302 = 76.8% (71.7%, 81.2%) | 135/155 = 87.1% (80.9%, 91.5%) | 97/147 = 66.0% (58.0%, 73.2%) |
| Yes | 18/27 = 66.7% (47.8%, 81.4%) | 6/7 = 85.7% (48.7%, 97.4%) | 12/20 = 60.0% (38.7%, 78.1%) |
| Ever smoked cigarettes or marijuana |  |  |  |
| No | 149/188 = 79.3% (72.9%, 84.4%) | 99/113 = 87.6% (80.3%, 92.5%) | 50/75 = 66.7% (55.4%, 76.3%) |
| Yes | 101/141 = 71.6% (63.7%, 78.4%) | 42/49 = 85.7% (73.3%, 92.9%) | 59/92 = 64.1% (53.9%, 73.2%) |
| Days since SARS-CoV-2 diagnosis at enrollment |  |  |  |
| <28 | 24/28 = 85.7% (68.5%, 94.3%) | 18/21 = 85.7% (65.4%, 95.0%) | 6/7 = 85.7% (48.7%, 97.4%) |
| 28-41 | 52/70 = 74.3% (63.0%, 83.1%) | 42/46 = 91.3% (79.7%, 96.6%) | 10/24 = 41.7% (24.5%, 61.2%) |
| 42-55 | 61/86 = 70.9% (60.6%, 79.5%) | 40/49 = 81.6% (68.6%, 90.0%) | 21/37 = 56.8% (40.9%, 71.3%) |
| 56+ | 113/145 = 77.9% (70.5%, 83.9%) | 41/46 = 89.1% (77.0%, 95.3%) | 72/99 = 72.7% (63.2%, 80.5%) |

| **Table E. Associations of COVID-19 severity by age and sex assigned at birth with nAb ID50/ID80 titer at enrollment after adjusting for participants’ medical history, race/ethnicity, BMI, region, and days since SARS-CoV-2 diagnosis.** | | | | | | | | |
| --- | --- | --- | --- | --- | --- | --- | --- | --- |
|  | **Response Rate** | | | | **Titer** | | | |
| **Comparison** | **OR** | **95% CI** | **p-value** | **q-value** | **GMR** | **95% CI** | **p-value** | **q-value** |
| **ID50** |  |  |  |  |  |  |  |  |
| Age 55+ vs 18-55 in asymptomatic | 1.35 | [0.28, 6.53] | 0.709 | 0.752 | 1.16 | [0.45, 2.97] | 0.758 | 0.758 |
| Age 55+ vs 18-55 in symptomatic outpatient | **5.96** | **[1.51, 23.5]** | **0.011** | **0.05** | **3.94** | **[2.11, 7.34]** | **<0.001** | **<0.001** |
| Age 55+ vs 18-55 in hospitalized | 2.98 | [0.45, 19.95] | 0.26 | 0.33 | 1.72 | [0.94, 3.15] | 0.08 | 0.102 |
| Symptomatic outpatient vs asymptomatic in Age 18-55 | 2.36 | [0.69, 8.11] | 0.172 | 0.251 | **3.47** | **[1.75, 6.86]** | **<0.001** | **0.001** |
| Hospitalized vs symptomatic outpatient in Age 18-55 | **4.72** | **[1.18, 18.96]** | **0.029** | **0.1** | **5.5** | **[3.01, 10.04]** | **<0.001** | **<0.001** |
| Symptomatic outpatient vs asymptomatic in Age 55+ | **10.43** | **[1.77, 61.53]** | **0.01** | **0.05** | **11.78** | **[4.6, 30.17]** | **<0.001** | **<0.001** |
| Hospitalized vs symptomatic outpatient in Age 55+ | 2.36 | [0.35, 16.12] | 0.38 | 0.444 | **2.4** | **[1.24, 4.63]** | **0.009** | **0.013** |
| Male vs female in asymptomatic | 0.26 | [0.06, 1.07] | 0.062 | 0.175 | 0.53 | [0.22, 1.24] | 0.143 | 0.166 |
| Male vs female in symptomatic outpatient | 2.71 | [0.72, 10.25] | 0.142 | 0.249 | 1.53 | [0.82, 2.84] | 0.179 | 0.192 |
| Male vs female in hospitalized | 3.19 | [0.59, 17.35] | 0.179 | 0.251 | **2.41** | **[1.31, 4.45]** | **0.005** | **0.008** |
| Symptomatic outpatient vs asymptomatic in female | 1.27 | [0.29, 5.64] | 0.752 | 0.752 | **3.24** | **[1.48, 7.12]** | **0.003** | **0.006** |
| Hospitalized vs symptomatic outpatient in female | 3.31 | [0.8, 13.69] | 0.098 | 0.228 | **3.16** | **[1.68, 5.93]** | **<0.001** | **0.001** |
| Symptomatic outpatient vs asymptomatic in male | **13.34** | **[3.05, 58.4]** | **0.001** | **0.008** | **9.43** | **[4.32, 20.57]** | **<0.001** | **<0.001** |
| Hospitalized vs symptomatic outpatient in male | 3.9 | [0.69, 22.11] | 0.124 | 0.247 | **4.97** | **[2.63, 9.4]** | **<0.001** | **<0.001** |
| **ID80** |  |  |  |  |  |  |  |  |
| Age 55+ vs 18-55 in asymptomatic | 0.85 | [0.22, 3.33] | 0.815 | 0.815 | 1.01 | [0.5, 2.05] | 0.981 | 0.981 |
| Age 55+ vs 18-55 in symptomatic outpatient | **4.74** | **[1.67, 13.49]** | **0.004** | **0.011** | **2.74** | **[1.71, 4.38]** | **<0.001** | **<0.001** |
| Age 55+ vs 18-55 in hospitalized | **6.32** | **[1.08, 36.97]** | **0.041** | **0.057** | **1.8** | **[1.14, 2.84]** | **0.012** | **0.015** |
| Symptomatic outpatient vs asymptomatic in age 18-55 | **4.62** | **[1.73, 12.29]** | **0.002** | **0.01** | **2.88** | **[1.72, 4.81]** | **<0.001** | **<0.001** |
| Hospitalized vs symptomatic outpatient in age 18-55 | **4.75** | **[1.59, 14.18]** | **0.005** | **0.012** | **4.49** | **[2.85, 7.06]** | **<0.001** | **<0.001** |
| Symptomatic outpatient vs asymptomatic in age 55+ | **25.78** | **[5.36, 123.94]** | **<0.001** | **<0.001** | **7.81** | **[3.84, 15.87]** | **<0.001** | **<0.001** |
| Hospitalized vs symptomatic outpatient in age 55+ | **6.33** | **[1.1, 36.49]** | **0.039** | **0.057** | **2.94** | **[1.79, 4.83]** | **<0.001** | **<0.001** |
| Male vs female in asymptomatic | 0.64 | [0.2, 2.02] | 0.445 | 0.479 | 0.84 | [0.44, 1.61] | 0.6 | 0.646 |
| Male vs female in symptomatic outpatient | 1.76 | [0.64, 4.82] | 0.273 | 0.318 | 1.56 | [0.98, 2.49] | 0.062 | 0.073 |
| Male vs female in hospitalized | 3.36 | [0.8, 14.14] | 0.098 | 0.125 | **2.02** | **[1.27, 3.21]** | **0.003** | **0.004** |
| Symptomatic outpatient vs asymptomatic in female | **5.38** | **[1.71, 16.94]** | **0.004** | **0.011** | **3.1** | **[1.71, 5.61]** | **<0.001** | **<0.001** |
| Hospitalized vs symptomatic outpatient in female | **3.82** | **[1.19, 12.28]** | **0.025** | **0.043** | **3.33** | **[2.07, 5.36]** | **<0.001** | **<0.001** |
| Symptomatic outpatient vs asymptomatic in male | **14.84** | **[4.21, 52.37]** | **<0.001** | **<0.001** | **5.75** | **[3.19, 10.34]** | **<0.001** | **<0.001** |
| Hospitalized vs symptomatic outpatient in male | **7.3** | **[1.69, 31.53]** | **0.008** | **0.015** | **4.32** | **[2.68, 6.98]** | **<0.001** | **<0.001** |
| Bold values are significant. | | | | | | | | |

| **Table F. Associations of COVID-19 severity, medical history, demographics, and days since SARS-CoV-2 diagnosis at enrollment (V1) with nAb ID50/ID80 titer fold-decline from V1 to V2 among participants with data available at both time points (V1 and V2).** | | | | | | | | |
| --- | --- | --- | --- | --- | --- | --- | --- | --- |
| **Comparison** | **ID50** | | | | **ID80** | | | |
|  | **GMR** | **95% CI** | **p-value** | **q-value** | **GMR** | **95% CI** | **p-value** | **q-value** |
| COVID-19 severity (overall test) | - | - | **0.025** |  | **-** | **-** | **0.001** |  |
| Symptomatic outpatient vs asymptomatic | 0.9 | [0.55, 1.46] | 0.657 | 0.906 | 1.32 | [0.86, 2.02] | 0.209 | 0.417 |
| Hospitalized vs symptomatic outpatient | **1.57** | **[1.07, 2.32]** | **0.024** | **0.168** | **1.66** | **[1.18, 2.34]** | **0.004** | **0.028** |
| Hospitalized vs asymptomatic | 1.41 | [0.84, 2.37] | 0.196 | 0.549 | **2.19** | **[1.39, 3.46]** | **0.001** | **0.014** |
| Diabetes | 1.15 | [0.65, 2.06] | 0.627 | 0.906 | 0.93 | [0.56, 1.55] | 0.784 | 0.844 |
| Hypertension | **0.62** | **[0.41, 0.92]** | **0.019** | **0.168** | **0.66** | **[0.46, 0.94]** | **0.022** | **0.062** |
| Age (>55 vs 18-55) | 1.2 | [0.85, 1.7] | 0.304 | 0.709 | **1.45** | **[1.07, 1.97]** | **0.018** | **0.062** |
| Sex assigned at birth (male vs female) | 1.4 | [1, 1.95] | 0.051 | 0.238 | **1.46** | **[1.09, 1.96]** | **0.012** | **0.056** |
| COPD/emphysema/asthma | 1.03 | [0.6, 1.78] | 0.906 | 0.906 | 1.21 | [0.75, 1.96] | 0.428 | 0.499 |
| Current cigarettes/marijuana smoker | 0.93 | [0.51, 1.7] | 0.809 | 0.906 | 0.75 | [0.44, 1.28] | 0.295 | 0.417 |
| Ever cigarettes/marijuana smoker | 1.03 | [0.73, 1.45] | 0.867 | 0.906 | 1.15 | [0.85, 1.55] | 0.356 | 0.453 |
| Non-Hispanic Black vs White | 1.17 | [0.52, 2.64] | 0.703 | 0.906 | 0.62 | [0.3, 1.27] | 0.193 | 0.417 |
| Hispanic/Latino vs Non-His White | 0.85 | [0.4, 1.79] | 0.668 | 0.906 | 0.7 | [0.36, 1.36] | 0.296 | 0.417 |
| Others vs Non-His White | 1.96 | [0.73, 5.26] | 0.181 | 0.549 | 0.63 | [0.26, 1.5] | 0.298 | 0.417 |
| BMI (≥30 vs <30) | 1.03 | [0.74, 1.43] | 0.853 | 0.906 | 1.01 | [0.76, 1.35] | 0.923 | 0.923 |
| Region and days since SARS-CoV-2 diagnosis were adjusted in the model. Bold values are significant. | | | | | | | | |

| **Table G. Associations of COVID-19 severity (asymptomatic, symptomatic, hospitalized no O_2_, hospitalized O_2_, hospitalized intubation/ICU), medical history, and demographics with nAb responses overall and by region (Peru and US).** | | | | | | | | | | | |
| --- | --- | --- | --- | --- | --- | --- | --- | --- | --- | --- | --- |
|  | **ID50** | | | | | **ID80** | | | | | |
|  | **GMR^*^** | **95% CI** | **p-value** | **q-value** | | **GMR^*^** | **95% CI** | **p-value** | | | **q-value** |
| **A. Total (n=329)** |  |  |  |  | |  |  |  | | |  |
| COVID-19 severity (overall test) | **-** | **-** | **<0.001** |  | | **-** | **-** | **<0.001** | | |  |
| Symptomatic outpatient vs asymptomatic | **5.64** | **[3.16, 10.07]** | **<0.001** | **<0.001** | | **4.14** | **[2.68, 6.4]** | **<0.001** | | | **<0.001** |
| Hospitalized, no O_2_ vs symptomatic outpatient | **3.19** | **[1.44, 7.05]** | **0.004** | **0.014** | | **2.95** | **[1.63, 5.34]** | **<0.001** | | | **0.002** |
| Hospitalized, O_2_ vs no O_2_ | 1.04 | [0.45, 2.4] | 0.922 | 0.978 | | 1.19 | [0.64, 2.23] | 0.585 | | | 0.864 |
| Hospitalized, intubation/ICU vs no intubation/ICU | 1.74 | [0.9, 3.39] | 0.103 | 0.22 | | 1.41 | [0.86, 2.32] | 0.178 | | | 0.333 |
| Diabetes | **2.38** | **[1.31, 4.31]** | **0.005** | **0.014** | | **1.8** | **[1.16, 2.82]** | **0.01** | | | **0.03** |
| Hypertension | **0.48** | **[0.29, 0.78]** | **0.003** | **0.014** | | 0.72 | [0.5, 1.04] | 0.083 | | | 0.177 |
| Age (>55 vs 18-55) | **2.49** | **[1.64, 3.78]** | **<0.001** | **<0.001** | | **2.06** | **[1.51, 2.82]** | **<0.001** | | | **<0.001** |
| Sex assigned at birth (male vs female) | 1.48 | [0.99, 2.21] | 0.059 | 0.147 | | **1.52** | **[1.12, 2.06]** | **0.007** | | | **0.026** |
| COPD/emphysema/asthma | 0.9 | [0.48, 1.69] | 0.743 | 0.928 | | 1.06 | [0.66, 1.69] | 0.811 | | | 0.879 |
| Current cigarettes/marijuana smoker | 1.01 | [0.48, 2.13] | 0.978 | 0.978 | | 1.06 | [0.61, 1.86] | 0.829 | | | 0.879 |
| Ever cigarettes/marijuana smoker | 0.8 | [0.52, 1.22] | 0.298 | 0.496 | | 0.94 | [0.68, 1.29] | 0.691 | | | 0.864 |
| Non-Hispanic Black vs White | 0.95 | [0.47, 1.93] | 0.883 | 0.978 | | 1.22 | [0.72, 2.08] | 0.462 | | | 0.769 |
| Hispanic/Latino vs Non-His White | 1.52 | [0.6, 3.89] | 0.381 | 0.572 | | 1.16 | [0.58, 2.35] | 0.674 | | | 0.864 |
| Others vs Non-His White | 1.36 | [0.49, 3.74] | 0.551 | 0.752 | | 1.06 | [0.5, 2.26] | 0.879 | | | 0.879 |
| BMI (≥30 vs <30) | 1.34 | [0.9, 2] | 0.155 | 0.29 | | 1.41 | [1.04, 1.9] | 0.025 | | | 0.063 |
|  |  |  |  | |  |  |  | |  |  | |
| **B. By region (Peru n=162; USA, n=167)** |  |  |  | |  |  |  | |  |  | |
| **Peru** |  |  |  | |  |  |  | |  |  | |
| COVID-19 severity (overall test) | **-** | **-** | **<0.001** | |  | **-** | **-** | | **<0.001** |  | |
| Symptomatic outpatient vs asymptomatic | **4.25** | **[2.01, 8.97]** | **<0.001** | | **0.003** | **3.69** | **[2.04, 6.69]** | | **<0.001** | **<0.001** | |
| Hospitalized, no O_2_ vs symptomatic outpatient | **2.86** | **[1.25, 6.53]** | **0.014** | | **0.055** | **2.81** | **[1.46, 5.41]** | | **0.002** | **0.014** | |
| Hospitalized, O_2_ vs no O_2_ | 1.96 | [0.8, 4.81] | 0.145 | | 0.218 | 1.91 | [0.93, 3.89] | | 0.078 | 0.121 | |
| Hospitalized, intubation/ICU vs no intubation/ICU | 1.18 | [0.51, 2.71] | 0.701 | | 0.841 | 1.1 | [0.57, 2.12] | | 0.788 | 0.788 | |
| Diabetes | 1.98 | [0.88, 4.46] | 0.101 | | 0.195 | 1.78 | [0.94, 3.39] | | 0.081 | 0.121 | |
| Hypertension | **0.35** | **[0.17, 0.69]** | **0.003** | | **0.018** | **0.57** | **[0.33, 0.99]** | | **0.048** | **0.116** | |
| Age (>55 vs 18-55) | **2** | **[1.11, 3.58]** | **0.021** | | **0.06** | **1.89** | **[1.19, 3]** | | **0.008** | **0.032** | |
| Sex assigned at birth (male vs female) | 1.39 | [0.82, 2.35] | 0.226 | | 0.301 | 1.39 | [0.91, 2.11] | | 0.127 | 0.17 | |
| COPD/emphysema/asthma | **0.3** | **[0.1, 0.85]** | **0.025** | | **0.06** | **0.38** | **[0.17, 0.89]** | | **0.026** | **0.079** | |
| Current cigarettes/marijuana smoker | 0.35 | [0.1, 1.27] | 0.114 | | 0.195 | 0.4 | [0.14, 1.1] | | 0.077 | 0.121 | |
| Ever cigarettes/marijuana smoker | 1.06 | [0.6, 1.87] | 0.844 | | 0.844 | 1.18 | [0.75, 1.85] | | 0.485 | 0.583 | |
| BMI (≥30 vs <30) | 1.07 | [0.63, 1.81] | 0.805 | | 0.844 | 1.13 | [0.75, 1.72] | | 0.56 | 0.611 | |
| **USA** |  |  |  | |  |  |  | |  |  | |
| COVID-19 severity (overall test) | **-** | **-** | **<0.001** | |  | **-** | **-** | | **<0.001** |  | |
| Symptomatic outpatient vs asymptomatic | **9.88** | **[3.95, 24.75]** | **<0.001** | | **<0.001** | **5.35** | **[2.76, 10.36]** | | **<0.001** | **<0.001** | |
| Hospitalized, no O_2_ vs symptomatic outpatient | **7.99** | **[1.36, 46.93]** | **0.023** | | **0.085** | **5.41** | **[1.51, 19.35]** | | **0.01** | **0.051** | |
| Hospitalized, O_2_ vs no O_2_ | 0.22 | [0.04, 1.32] | 0.1 | | 0.218 | 0.39 | [0.11, 1.42] | | 0.155 | 0.268 | |
| Hospitalized, intubation/ICU vs no intubation/ICU | 2.74 | [0.93, 8.06] | 0.069 | | 0.207 | 1.91 | [0.88, 4.14] | | 0.105 | 0.225 | |
| Diabetes | **2.85** | **[1.19, 6.83]** | **0.02** | | **0.085** | 1.85 | [0.99, 3.47] | | 0.057 | 0.143 | |
| Hypertension | 0.54 | [0.26, 1.12] | 0.102 | | 0.218 | 0.78 | [0.46, 1.32] | | 0.355 | 0.444 | |
| Age (>55 vs 18-55) | **3.39** | **[1.77, 6.49]** | **<0.001** | | **0.002** | **2.39** | **[1.5, 3.82]** | | **<0.001** | **0.003** | |
| Sex assigned at birth (male vs female) | 1.53 | [0.82, 2.87] | 0.185 | | 0.308 | **1.59** | **[1.01, 2.5]** | | **0.046** | **0.137** | |
| COPD/emphysema/asthma | 1.37 | [0.6, 3.09] | 0.456 | | 0.488 | 1.53 | [0.85, 2.75] | | 0.161 | 0.268 | |
| Current cigarettes/marijuana smoker | 1.53 | [0.59, 4.01] | 0.384 | | 0.48 | 1.47 | [0.74, 2.94] | | 0.273 | 0.372 | |
| Ever cigarettes/marijuana smoker | 0.69 | [0.36, 1.29] | 0.244 | | 0.366 | 0.87 | [0.55, 1.37] | | 0.541 | 0.624 | |
| Non-Hispanic Black vs White | 1.23 | [0.53, 2.86] | 0.624 | | 0.624 | 1.4 | [0.77, 2.57] | | 0.272 | 0.372 | |
| Hispanic/Latino vs Non-His White | 1.6 | [0.58, 4.45] | 0.368 | | 0.48 | 1.16 | [0.56, 2.42] | | 0.693 | 0.693 | |
| Others vs Non-His White | 1.57 | [0.51, 4.8] | 0.431 | | 0.488 | 1.18 | [0.53, 2.64] | | 0.686 | 0.693 | |
| BMI (≥30 vs <30) | 1.51 | [0.83, 2.75] | 0.181 | | 0.308 | **1.58** | **[1.02, 2.43]** | | **0.04** | **0.137** | |
| ^*^: Geometric mean ratio; Region and days since SARS-CoV-2 diagnosis were adjusted in the model. Bold values are significant. | | | | | | | | | | | |

| **Table H. Association of corticosteroid use on nAb responses in symptomatic outpatients and hospitalized individuals after adjusting for age, sex assigned at birth, BMI, diabetes, hypertension, and days since SARS-CoV-2 diagnosis** | | | | | | | |
| --- | --- | --- | --- | --- | --- | --- | --- |
|  |  | **Response Rate** | | | **Titer** | | |
| **Titer** | **Comparison** | **OR** | **95% CI** | **p-value** | **GMR** | **95% CI** | **p-value** |
| ID50 | Yes vs No in Symptomatic | 4.51 | [0.22, 91.41] | 0.326 | 2.65 | [0.89, 7.85] | 0.079 |
|  | Yes vs No in Hospitalized | 1.99 | [0.32, 12.25] | 0.456 | 1.26 | [0.69, 2.33] | 0.451 |
| ID80 | Yes vs No in Symptomatic | 8.38 | [0.42, 168.23] | 0.165 | **2.28** | **[0.99, 5.28]** | **0.054** |
|  | Yes vs No in Hospitalized | 3.53 | [0.6, 20.63] | 0.162 | 1.51 | [0.94, 2.43] | 0.085 |

| **Table I. NAb geometric mean titer (GMT) and 95% CI at the enrollment visit (V1) and 2-month post-enrollment visit (V2) and GMT ratio (V/V2) among participants with data available at both time points (V1 and V2).** | | | | | | | |
| --- | --- | --- | --- | --- | --- | --- | --- |
|  |  |  | **V1** | | **V2** | | **Ratio (V1/V2)** |
| **Titer** | **Characteristic** | **Level** | **N** | **GMT (95% CI)** | **GMT (95% CI)** | **GMT** | |
| **ID50** | Enrolled | Y | 186 | 464.3 (333.1, 647.3) | 132.7 (102.4, 171.9) | 3.5 | |
|  | Age category | 18 - 55 | 113 | 267.3 (174.4, 409.6) | 77.4 (56.5, 106) | 3.5 | |
|  |  | 55+ | 73 | 1091.8 (678, 1758.1) | 305.4 (209.1, 446) | 3.6 | |
|  | Sex assigned at birth | Female | 93 | 276.8 (174.1, 440.1) | 102.5 (72.1, 145.8) | 2.7 | |
|  |  | Male | 93 | 778.9 (491.6, 1234.2) | 171.7 (117.5, 250.8) | 4.5 | |
|  | BMI category | <30 | 116 | 413.7 (272.2, 628.6) | 112.8 (82.5, 154.3) | 3.7 | |
|  |  | 30+ | 70 | 562.3 (322.3, 981.2) | 173.5 (110.1, 273.4) | 3.2 | |
|  | Days from SARS-CoV-2 diagnosis category | <28 | 16 | 577.9 (233.1, 1432.6) | 51.4 (25.3, 104.4) | 11.2 | |
|  |  | 28-41 | 45 | 805.3 (393.6, 1647.6) | 163.1 (95.9, 277.3) | 4.9 | |
|  |  | 42-55 | 50 | 377.1 (181.9, 781.5) | 140.3 (80.9, 243.4) | 2.7 | |
|  |  | 56+ | 75 | 365.9 (223.6, 598.8) | 138.2 (92.3, 207) | 2.6 | |
|  | SARS-CoV-2 severity at enrollment | Asymptomatic | 32 | 145.9 (75.4, 282.3) | 33.5 (21.9, 51.3) | 4.4 | |
|  |  | Symptomatic outpt | 87 | 250.8 (159.3, 395) | 102.8 (71.8, 147.2) | 2.4 | |
|  |  | Hospitalized | 67 | 1795.9 (1084.1, 2974.9) | 356.1 (239.5, 529.4) | 5 | |
|  | Currently smoke cigarettes or marijuana | N | 171 | 493.6 (348.4, 699.3) | 139.8 (106.8, 183.1) | 3.5 | |
|  |  | Y | 15 | 231.6 (72.1, 743.8) | 72.9 (27, 196.8) | 3.2 | |
|  | Ever smoked cigarettes or marijuana | N | 107 | 604.9 (395.2, 926) | 155.3 (111.2, 216.8) | 3.9 | |
|  |  | Y | 79 | 324.5 (191.2, 550.8) | 107.2 (70.9, 161.9) | 3 | |
|  | Hypertension | N | 145 | 475 (329, 686) | 122.2 (92.1, 162.1) | 3.9 | |
|  |  | Y | 41 | 428.4 (193.5, 948.5) | 177.3 (93.9, 334.8) | 2.4 | |
|  | COPD/emphysema/asthma | N | 168 | 475.8 (334, 677.9) | 131.8 (100.3, 173.2) | 3.6 | |
|  |  | Y | 18 | 370 (132.5, 1033.5) | 140.8 (57.2, 346.1) | 2.6 | |
|  | Diabetes | N | 171 | 388.9 (275.5, 548.9) | 116.1 (88.9, 151.6) | 3.3 | |
|  |  | Y | 15 | 3505.1 (1729.6, 7103.3) | 606.8 (296.9, 1240.2) | 5.8 | |
|  | Race/ethnicity | Hispanic - Latino/a | 103 | 797.5 (513.8, 1237.6) | 147.7 (104.8, 208) | 5.4 | |
|  |  | Black - Non-Hispanic | 8 | 286.4 (34.4, 2385) | 118.7 (21.1, 668.5) | 2.4 | |
|  |  | White - Non-Hispanic | 70 | 229 (136.2, 385) | 121.1 (78.3, 187.2) | 1.9 | |
|  |  | Other | 5 | 289.7 (19.3, 4349.3) | 62.5 (6.2, 629.4) | 4.6 | |
| **ID80** | Enrolled | Y | 186 | 95 (72.7, 124.1) | 34.4 (28.2, 41.9) | 2.8 | |
|  | Age category | 18 - 55 | 113 | 58.5 (42.6, 80.4) | 24.6 (19.7, 30.7) | 2.4 | |
|  |  | 55+ | 73 | 200.9 (131.4, 307.1) | 57.7 (40.9, 81.3) | 3.5 | |
|  | Sex assigned at birth | Female | 93 | 59.7 (41.9, 84.9) | 27.8 (21.2, 36.6) | 2.1 | |
|  |  | Male | 93 | 151.1 (102.8, 222.2) | 42.4 (31.9, 56.4) | 3.6 | |
|  | BMI category | <30 | 116 | 82.7 (58.7, 116.4) | 29.3 (23.1, 37.1) | 2.8 | |
|  |  | 30+ | 70 | 119.5 (77.5, 184.3) | 44.8 (31.7, 63.4) | 2.7 | |
|  | Days from SARS-CoV-2 diagnosis category | <28 | 16 | 93 (46.6, 185.7) | 14 (9.7, 20.3) | 6.6 | |
|  |  | 28-41 | 45 | 147.2 (78.5, 275.9) | 43.5 (28.5, 66.4) | 3.4 | |
|  |  | 42-55 | 50 | 82.9 (46.1, 148.9) | 35.8 (23.7, 54) | 2.3 | |
|  |  | 56+ | 75 | 80.3 (55.3, 116.6) | 35.1 (25.8, 47.9) | 2.3 | |
|  | SARS-CoV-2 severity at enrollment | Asymptomatic | 32 | 26.9 (17.3, 41.9) | 12.1 (9.7, 15.1) | 2.2 | |
|  |  | Symptomatic outpt | 87 | 56.3 (40.8, 77.8) | 25 (19.6, 31.9) | 2.3 | |
|  |  | Hospitalized | 67 | 341.7 (222.6, 524.5) | 85.3 (61.2, 119) | 4 | |
|  | Currently smoke cigarettes or marijuana | N | 171 | 99.4 (74.8, 132.1) | 34.8 (28.2, 42.8) | 2.9 | |
|  |  | Y | 15 | 56.5 (27.5, 116.3) | 29.8 (14.8, 60) | 1.9 | |
|  | Ever smoked cigarettes or marijuana | N | 107 | 115.2 (80.4, 165.2) | 39.6 (30.2, 51.9) | 2.9 | |
|  |  | Y | 79 | 73.1 (49, 109) | 28.4 (21.3, 37.8) | 2.6 | |
|  | Hypertension | N | 145 | 92 (68.2, 124.2) | 31.4 (25.2, 39.3) | 2.9 | |
|  |  | Y | 41 | 106 (57.3, 196) | 47.1 (30.5, 72.7) | 2.3 | |
|  | COPD/emphysema/asthma | N | 168 | 97.4 (73.4, 129.3) | 35 (28.4, 43.2) | 2.8 | |
|  |  | Y | 18 | 74.7 (30.4, 183.3) | 28.6 (14.9, 54.9) | 2.6 | |
|  | Diabetes | N | 171 | 82 (62.4, 107.7) | 30.7 (25.2, 37.3) | 2.7 | |
|  |  | Y | 15 | 508.9 (223.7, 1158.1) | 125.7 (56.7, 278.6) | 4 | |
|  | Race/ethnicity | Hispanic - Latino/a | 103 | 155.7 (107.8, 224.9) | 42.6 (32.2, 56.2) | 3.7 | |
|  |  | Black - Non-Hispanic | 8 | 55 (10, 301.4) | 46.7 (11.2, 195.1) | 1.2 | |
|  |  | White - Non-Hispanic | 70 | 52.1 (35.6, 76.5) | 24.6 (18.5, 32.8) | 2.1 | |
|  |  | Other | 5 | 37.8 (7.1, 200.1) | 26.8 (5.1, 142.2) | 1.4 | |

| **Table J. Association of angiotensin-receptor blocker (ARBs) and/or ACE inhibitor Use (n=49 Use and n=26 no Use) and nAb responses, adjusting for COVID-19 severity, age, sex assigned at birth, diabetes, and days since SARS-CoV-2 diagnosis among participants with hypertension.** | | | | | | |
| --- | --- | --- | --- | --- | --- | --- |
|  | **Response Rate** | | |  | **Titer** | |
| **Titer** | **OR** | **95% CI** | **p-value** | **GMR** | **95% CI** | **p-value** |
| ID50 | 0.65 | [0.16, 2.48] | 0.54 | 0.87 | [0.33, 2.3] | 0.777 |
| ID80 | 0.65 | [0.14, 2.67] | 0.554 | 0.84 | [0.41, 1.7] | 0.626 |

| **Table K. Participant characteristics by responder or non-responder and nAb ID50 or ID80 titer at enrollment.** | | | | | |
| --- | --- | --- | --- | --- | --- |
| **Characteristic** | **Level** | **ID50 Non-responder** | **ID50 Responder** | **ID80 Non-responder** | **ID80 Responder** |
| Enrolled | N (%) | 48 (14.5%) | 281 (85.5%) | 79 (23.9%) | 250 (76.1%) |
| Age | N | 48 | 281 | 79 | 250 |
|  | Mean (SD) | 44.2 (14.52) | 48.4 (15.21) | 43.7 (14.21) | 49.1 (15.26) |
|  | Median (25th, 75th %) | 40 (33, 58.2) | 50 (36, 60.8) | 39 (33, 58) | 51 (36, 61) |
|  | Min - Max | 24 - 82 | 18 - 86 | 22 - 82 | 18 - 86 |
| Age category | 18 - 55 | 34 (70.8%) | 167 (59.2%) | 57 (72.2%) | 144 (57.6%) |
|  | 55+ | 14 (29.2%) | 114 (40.6%) | 22 (27.8%) | 106 (42.4%) |
| Sex assigned at birth | Female | 23 (47.9%) | 140 (49.8%) | 44 (55.7%) | 119 (47.6%) |
|  | Male | 25 (52.1%) | 141 (50.2%) | 35 (44.3%) | 131 (52.4%) |
| BMI category | <30 | 29 (60.4%) | 177 (63.0%) | 53 (67.1%) | 153 (61.2%) |
|  | ≥30 | 19 (39.6%) | 104 (37.0%) | 26 (32.9%) | 97 (38.8%) |
| Days from SARS-CoV-2 onset | N | 48 | 281 | 79 | 250 |
|  | Mean (SD) | 52.2 (18.14) | 52.2 (18.72) | 52.5 (18.16) | 52.2 (18.8) |
|  | Median (25th, 75th %) | 50.5 (39.5, 65.2) | 53 (38, 67) | 51 (39, 67) | 53 (38, 66) |
|  | Min - Max | 24 - 115 | 13 - 120 | 13 - 115 | 13 - 120 |
| Days from SARS-CoV-2 diagnosis category | <28 | 2 (4.2%) | 26 (9.3%) | 4 (5.1%) | 24 (9.6%) |
|  | 28-41 | 11 (22.9%) | 59 (21.0%) | 18 (22.8%) | 52 (20.8%) |
|  | 42-55 | 15 (31.2%) | 71 (25.3%) | 25 (31.6%) | 61 (24.4%) |
|  | 56+ | 20 (41.7%) | 125 (44.5%) | 32 (40.5%) | 113 (45.2%) |
| SARS-CoV-2 severity at enrollment | Asymptomatic | 22 (45.8%) | 43 (15.3%) | 35 (44.3%) | 30 (12%) |
|  | Symptomatic outpatient | 19 (39.6%) | 113 (40.2%) | 34 (43%) | 98 (39.2%) |
|  | Hospitalized | 7 (14.6%) | 125 (44.5%) | 10 (12.7%) | 122 (48.8%) |
| Currently smoke cigarettes or marijuana | N (%) | 8 (16.7%) | 19 (6.8%) | 9 (11.4%) | 18 (7.2%) |
| Ever smoked cigarettes or marijuana | N (%) | 27 (56.2%) | 114 (40.6%) | 40 (50.6%) | 101 (40.4%) |
| Hypertension | N (%) | 20 (41.7%) | 55 (19.6%) | 23 (29.1%) | 52 (20.8%) |
| COPD/emphysema/ asthma | N (%) | 5 (10.4%) | 30 (10.7%) | 10 (12.7%) | 25 (10%) |
| Diabetes | N (%) | 4 (8.3%) | 38 (13.5%) | 6 (7.6%) | 36 (14.4%) |

| **Table L. Associations of COVID-19 severity, medical history, demographics and days since SARS-CoV-2 diagnosis with nAb nonresponse at enrollment.** | | | | | | | | |
| --- | --- | --- | --- | --- | --- | --- | --- | --- |
|  | **ID50** | | | | **ID80** | | | |
| **Characteristic** | **OR** | **ID50 95% CI** | **p-value** | **q-value** | **OR** | **ID80 95% CI** | **p-value** | **q-value** |
| COVID-19 severity (overall test) |  |  | **<0.001** |  |  |  | **<0.001** |  |
| Symptomatic outpatient vs asymptomatic | **0.27** | **[0.1, 0.69]** | **0.007** | **0.031** | **0.15** | **[0.06, 0.34]** | **<0.001** | **<0.001** |
| Hospitalized vs symptomatic outpatient | **0.23** | **[0.07, 0.67]** | **0.009** | **0.031** | **0.2** | **[0.08, 0.48]** | **0.001** | **0.003** |
| Diabetes | 0.25 | [0.05, 0.92] | 0.056 | 0.145 | **0.3** | **[0.08, 0.92]** | **0.046** | **0.117** |
| Hypertension | **6.19** | **[2.39, 17.21]** | **<0.001** | **0.003** | **3.09** | **[1.33, 7.39]** | **0.01** | **0.031** |
| Age (>55 vs 18-55) | **0.29** | **[0.11, 0.7]** | **0.009** | **0.031** | **0.32** | **[0.15, 0.66]** | **0.003** | **0.014** |
| Sex assigned at birth (male vs female) | 0.82 | [0.37, 1.77] | 0.61 | 0.802 | 0.67 | [0.34, 1.31] | 0.249 | 0.36 |
| COPD/emphysema/asthma | 0.86 | [0.24, 2.68] | 0.802 | 0.802 | 1.17 | [0.43, 3] | 0.754 | 0.891 |
| Current cigarettes/marijuana smoker | 0.83 | [0.24, 2.63] | 0.754 | 0.802 | 0.45 | [0.14, 1.39] | 0.179 | 0.29 |
| Ever cigarettes/marijuana smoker | 1.74 | [0.77, 3.95] | 0.182 | 0.338 | 1.61 | [0.81, 3.22] | 0.176 | 0.29 |
| Non-Hispanic Black vs White | 1.58 | [0.5, 4.9] | 0.426 | 0.693 | 1.04 | [0.36, 2.96] | 0.94 | 0.988 |
| Hispanic/Latino vs Non-His White | 0.22 | [0.01, 1.38] | 0.178 | 0.338 | 0.69 | [0.17, 2.41] | 0.568 | 0.739 |
| Others vs Non-His White | 0.72 | [0.09, 3.75] | 0.716 | 0.802 | 0.99 | [0.21, 4.27] | 0.988 | 0.988 |
| BMI (≥30 vs <30) | 0.83 | [0.37, 1.79] | 0.638 | 0.802 | 0.51 | [0.25, 1] | 0.054 | 0.117 |
| Region and days since SARS-CoV-2 diagnosis were adjusted in the model. Bold values are significant. | | | | | | | | |
