# Supplementary material for: Neutralizing antibody responses over time in demographically and clinically diverse individuals recovered from SARS-CoV-2 infection in the United States and Peru: A cohort study
Source: PLoS Med. 2021 Dec 6;18(12):e1003868. doi: 10.1371/journal.pmed.1003868 (PMC8687542; doi:10.1371/journal.pmed.1003868)
Supplement: S2 Data — Supporting information figures A-G. Fig A. GMT ID80 at enrollment visit and 95% CI by enrollment group, severity, medical history, and days since COVID-19 onset in Americas. Figure B. Estimated age effect on nAb log-titers stratifying by COVID-19 severity adjusting for COVID-19 severity, medical history, other demographics, and days since SARS-CoV-2 diagnosis using GAM. Fig C. GMT of nAb ID50 (A) and ID80 (B) titer at the enrollment visit in Americas. White asterisks denote groups with fewer than 10 participants. Fig D. Estimated days since SARS-CoV-2 diagnosis (days) effect on nAb log-titers stratifying by COVID-19 severity adjusting for COVID-19 severity, medical history, and demographics using GAM. Fig E. Estimated BMI effect on nAb log-titers stratifying by COVID-19 severity adjusting for COVID-19 severity, medical history, other demographics, and days since SARS-CoV-2 diagnosis using GAM. Fig F. GMT at enrollment visit and 95% CI by enrollment group, severity, medical history, and days since SARS-CoV-2 diagnosis in the US and Peru. A. Peru, ID50. B. Peru, ID80. C. US, ID50. D. US, ID80. Fig G. nAb titers by prolonged viral shedding status and COVID-19 severity. BMI, body mass index; CI, confidence interval; COVID-19, Coronavirus Disease 2019; GAM, generalized additive model; GMT, geometric mean titer; nAb, neutralizing antibody; SARS-CoV-2, Severe Acute Respiratory Syndrome Coronavirus 2. (PDF) [file pmed.1003868.s005.pdf]

S2 Data. Supplemental figures A-G.

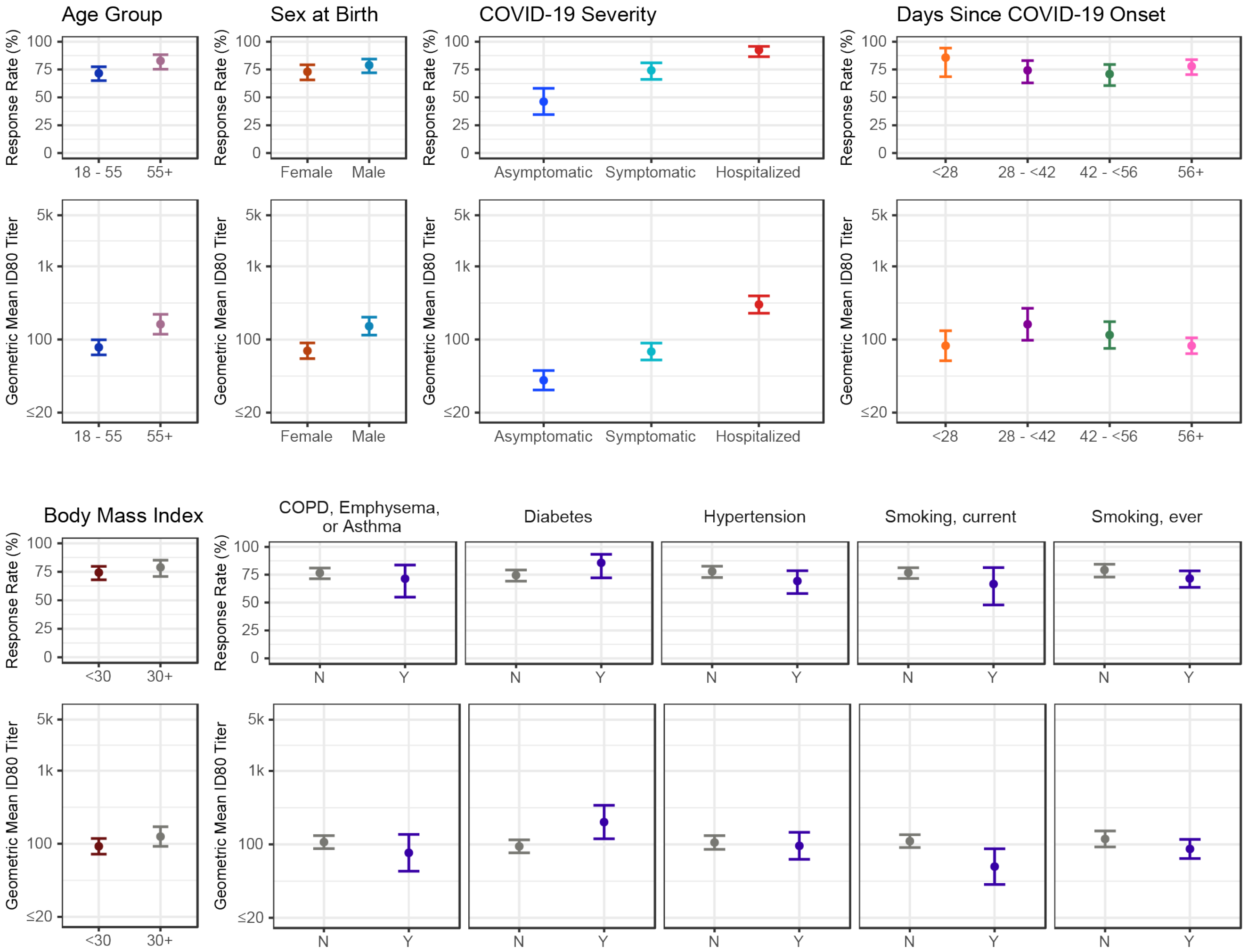

Figure A. Geometric mean titer (GMT) ID80 at enrollment visit and 95% CI by enrollment group, severity, medical history, and days since COVID-19 onset in Americas.

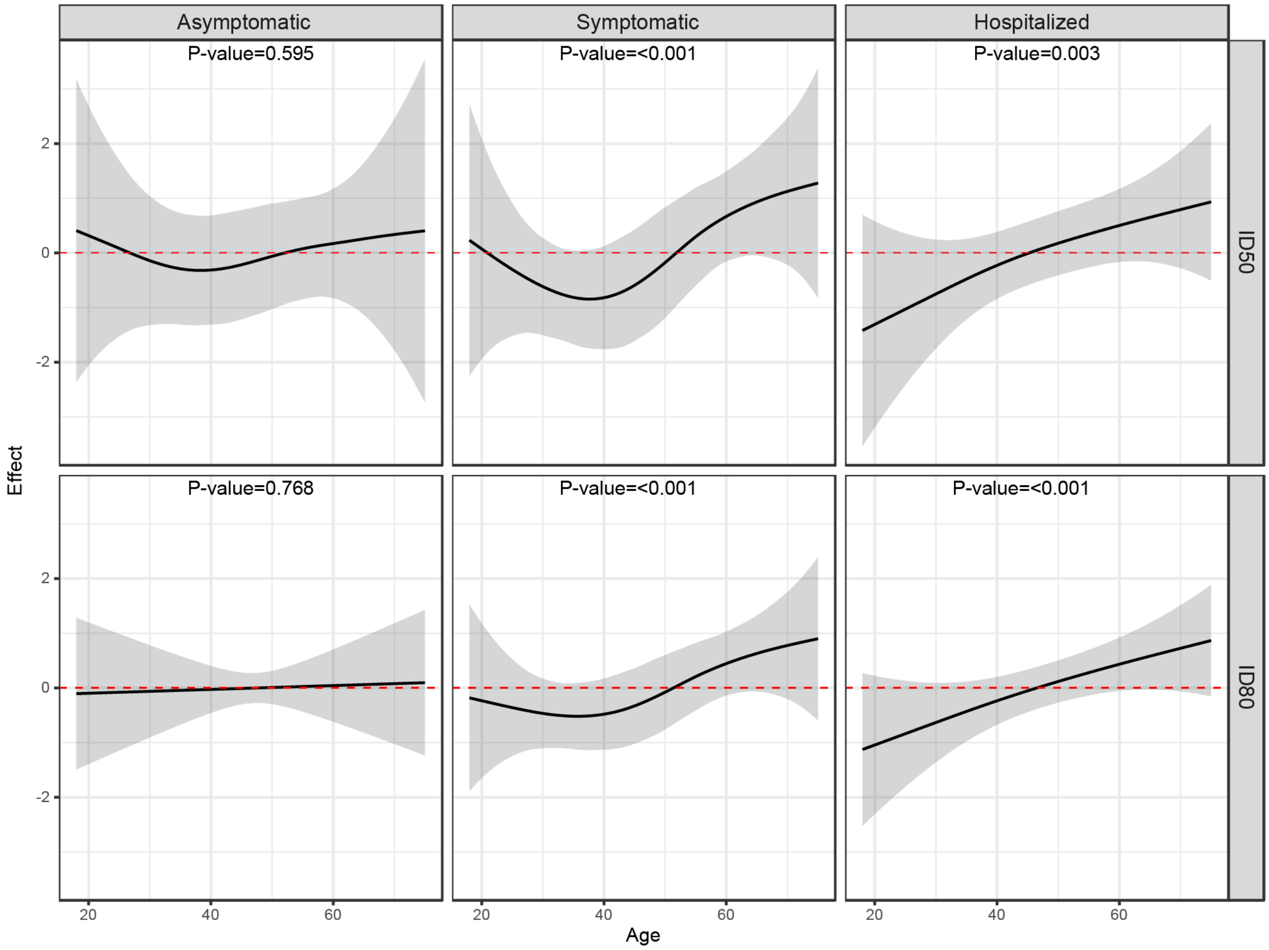

Figure B. Estimated age effect on nAb log-titers stratifying by COVID-19 severity adjusting for COVID-19 severity, medical history, other demographics, and days since SARS-CoV-2 diagnosis using generalized additive model.

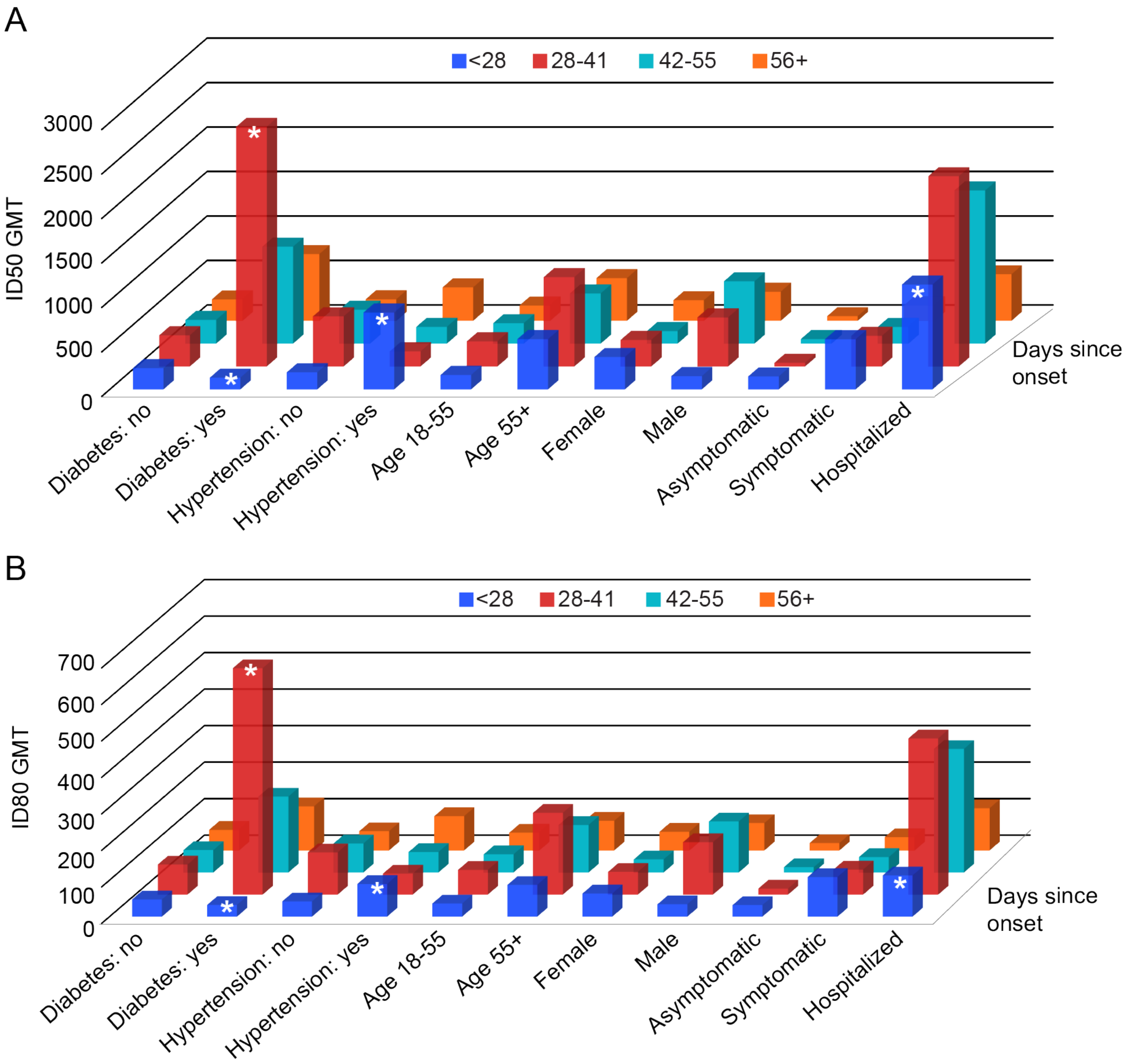

Figure C. Geometric mean titer (GMT) of nAb ID50 (A) and ID80 (B) titer at the enrollment visit in Americas. White asterisks denote groups with fewer than 10 participants.

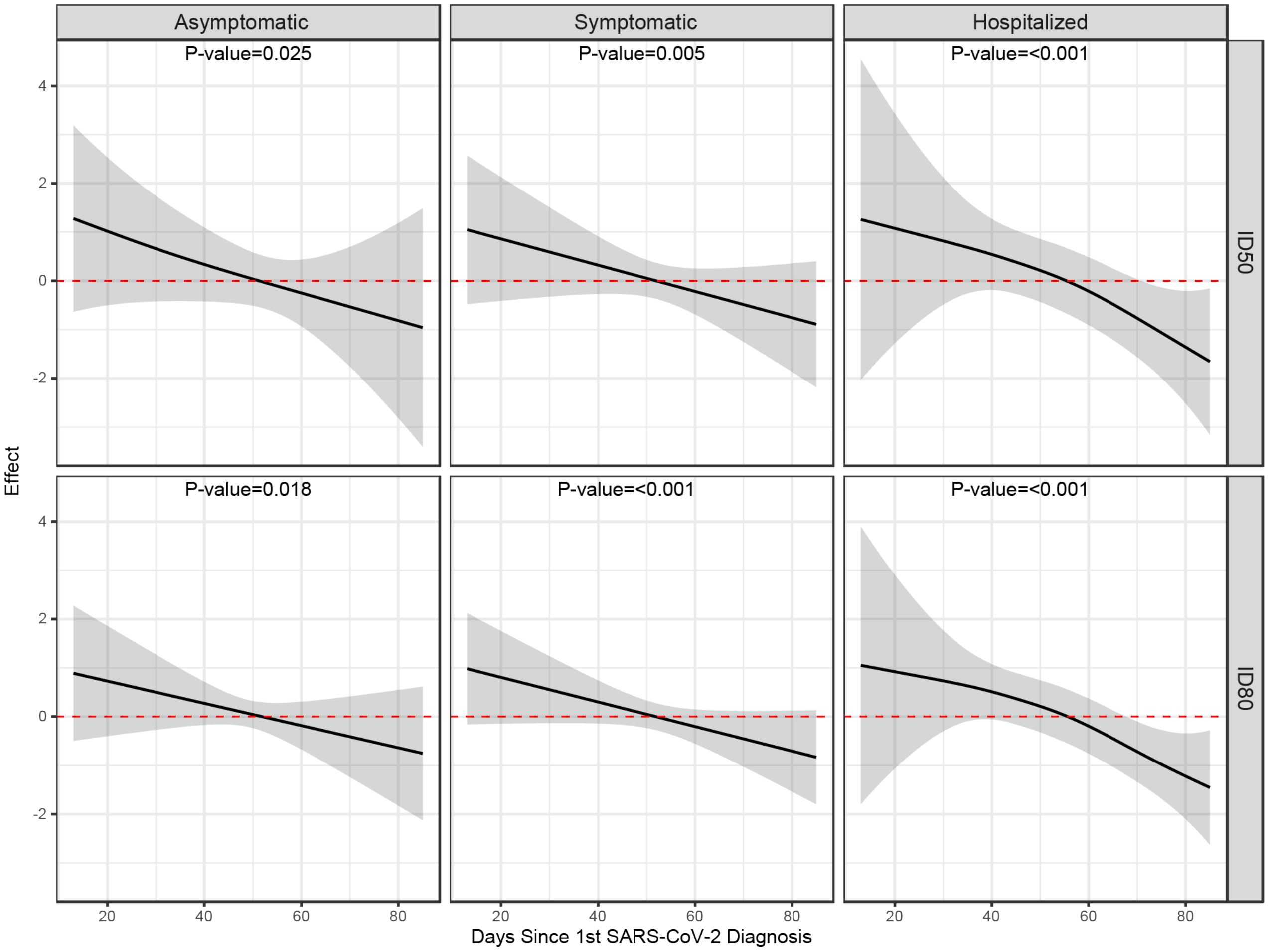

Figure D. Estimated days since SARS-CoV-2 diagnosis (days) effect on nAb log-titers stratifying by COVID-19 severity adjusting for COVID-19 severity, medical history, and demographics using generalized additive model.

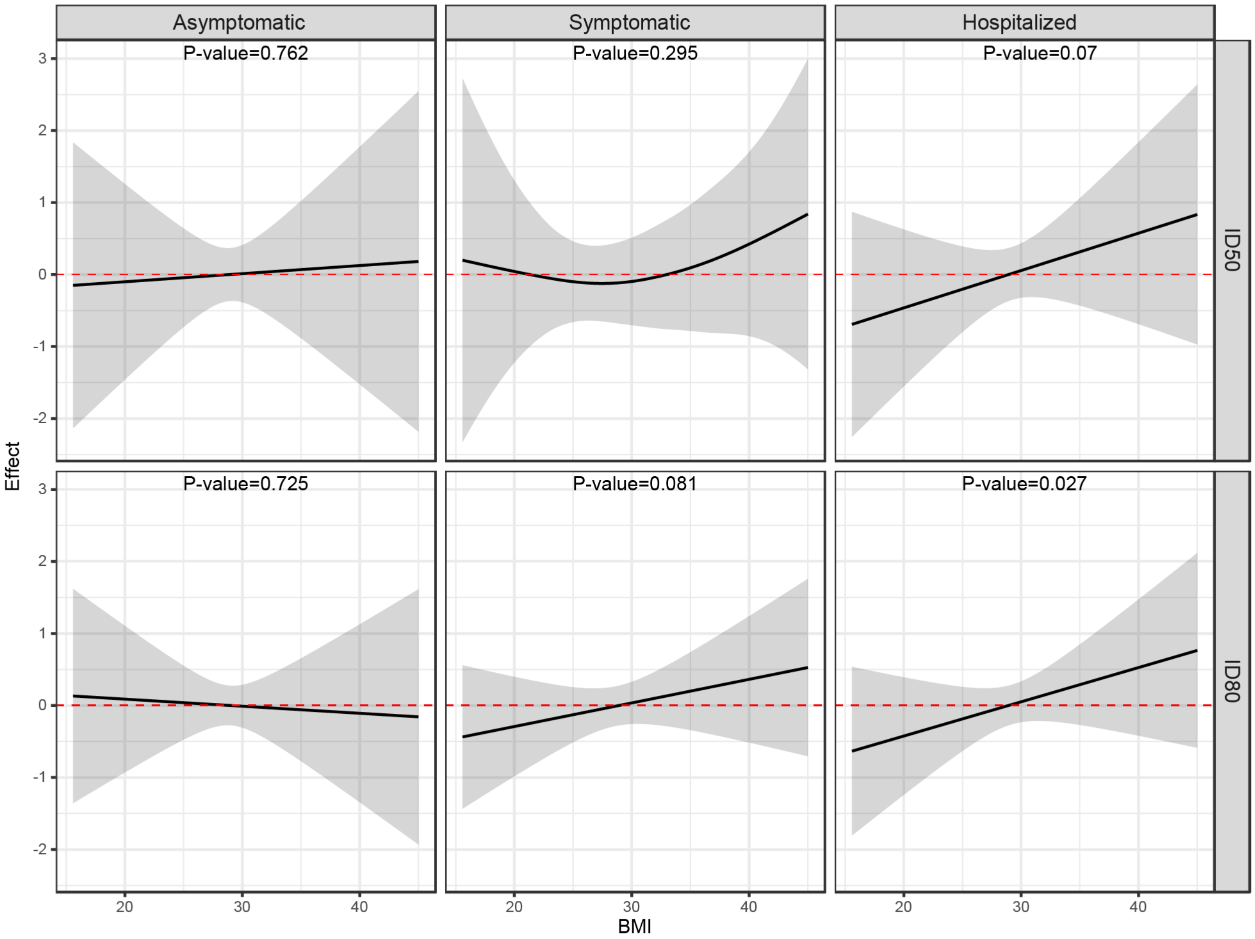

Figure E. Estimated BMI effect on nAb log-titers stratifying by COVID-19 severity adjusting for COVID-19 severity, medical history, other demographics, and days since SARS-CoV-2 diagnosis using generalized additive model.

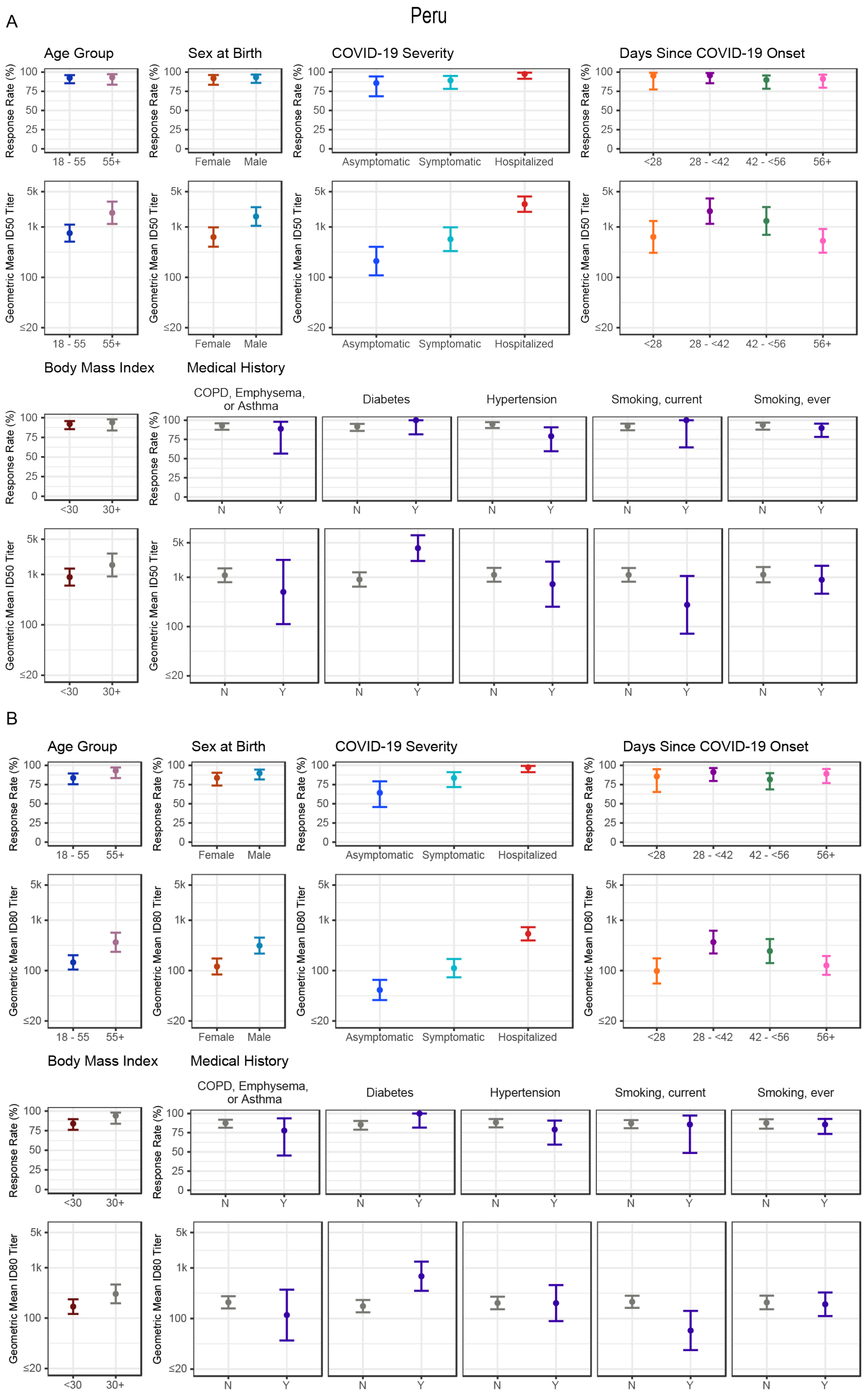

Figure F. Geometric mean titer (GMT) at enrollment visit and 95% CI by enrollment group, severity, medical history, and days since SARS-CoV-2 diagnosis in USA and Peru. A. Peru, ID50. B. Peru, ID80. C. US, ID50. D. US, ID80.

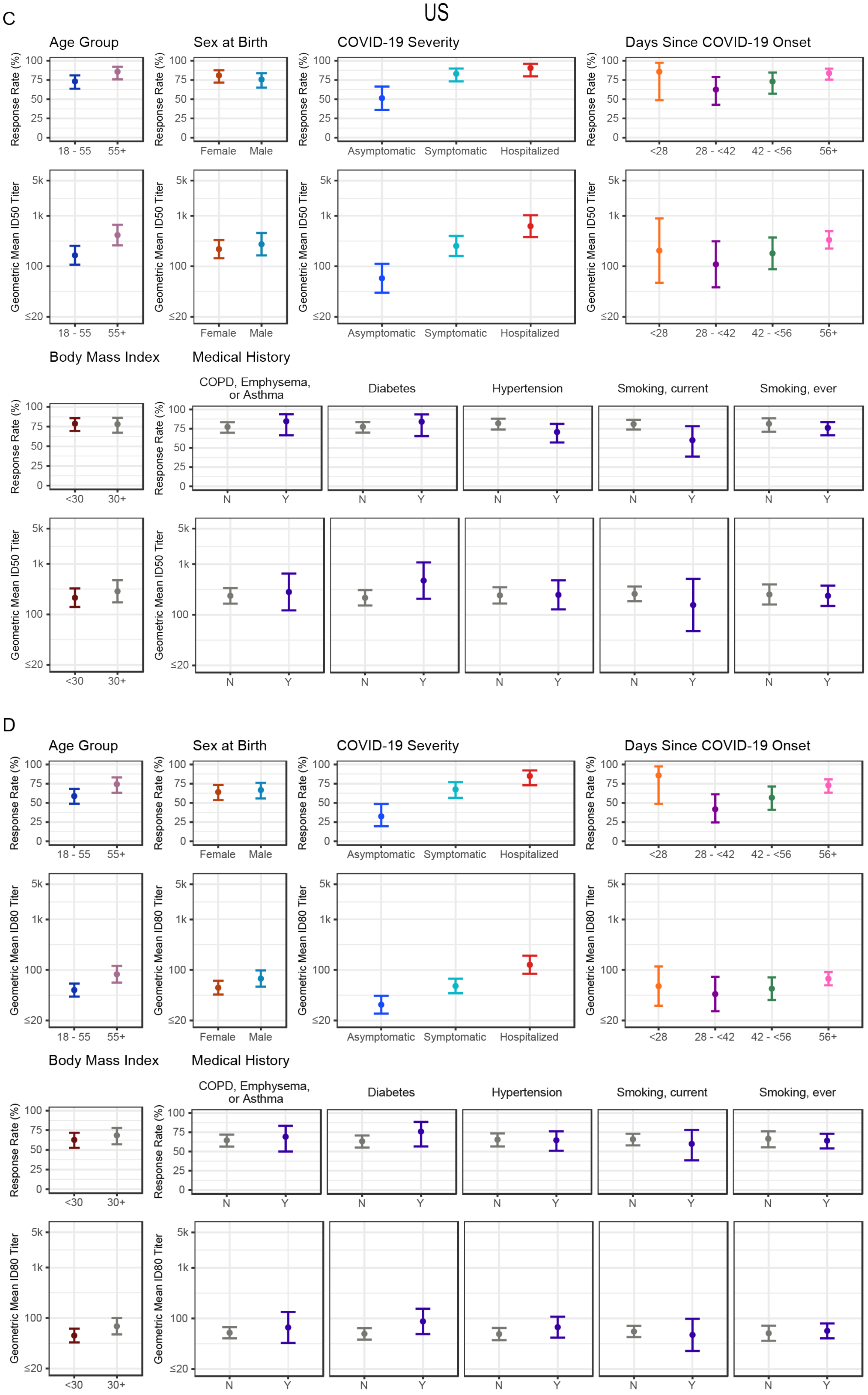
C
US
Age Group
Sex at Birth
COVID-19 Severity
Days Since COVID-19 Onset
Response Rate (%)
Geometric Mean ID50 Titer
18 - 55
55+
Female
Male
Asymptomatic
Symptomatic
Hospitalized
<28
28 - <42
42 - <56
56+
Body Mass Index
Medical History
COPD, Emphysema, or Asthma
Diabetes
Hypertension
Smoking, current
Smoking, ever
<30
30+
N
Y
D
Geometric Mean ID80 Titer

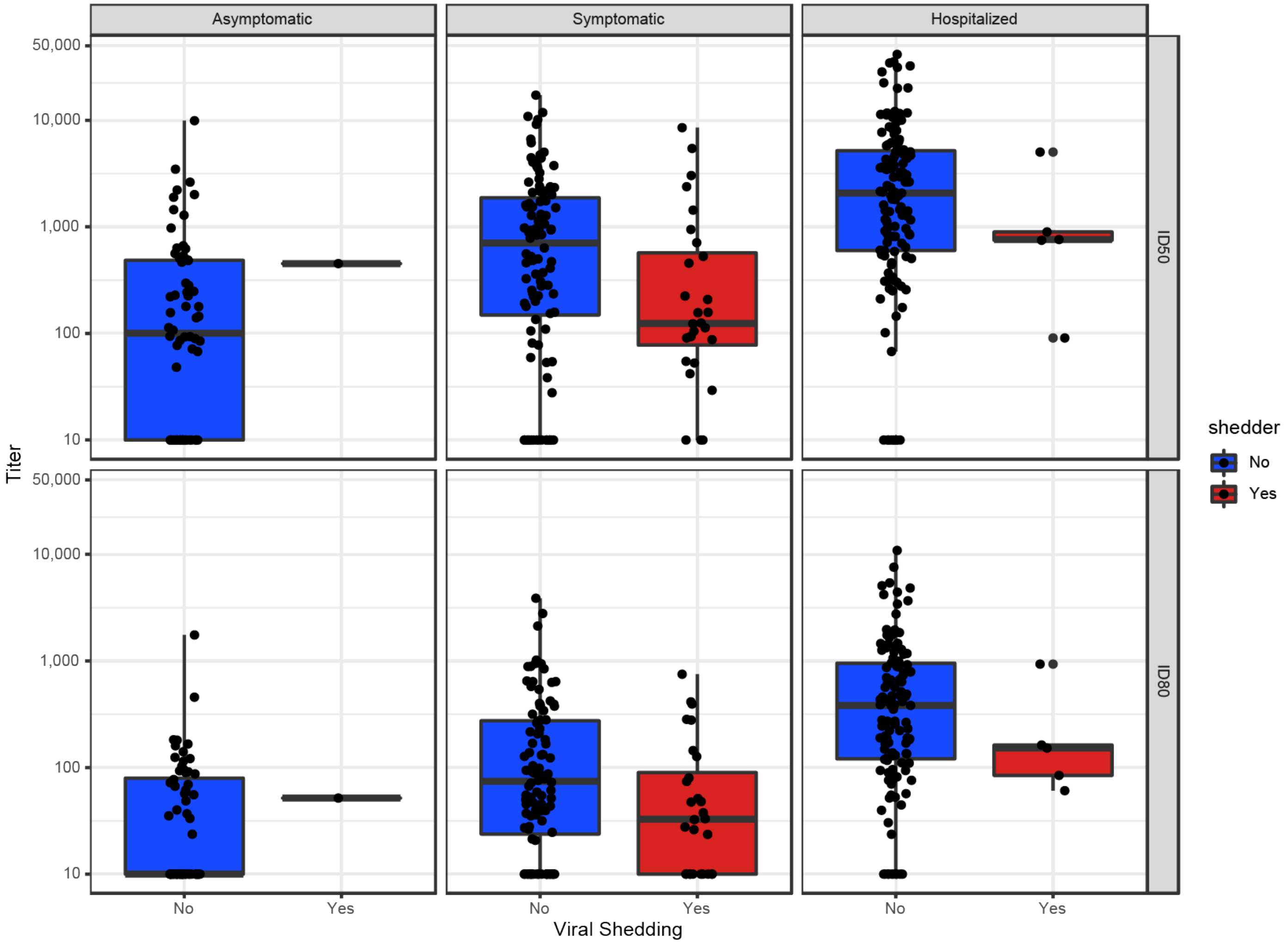

Figure G. NAb titers by prolonged viral shedding status and COVID-19 severity.
